# Nutrient-dependent cross-kingdom interactions in the hyphosphere of an arbuscular mycorrhizal fungus

**DOI:** 10.3389/fmicb.2023.1284648

**Published:** 2024-01-04

**Authors:** Maede Faghihinia, Larry J. Halverson, Hana Hršelová, Petra Bukovská, Martin Rozmoš, Michala Kotianová, Jan Jansa

**Affiliations:** ^1^Laboratory of Fungal Biology, Institute of Microbiology, Czech Academy of Sciences, Prague, Czechia; ^2^Department of Plant Pathology, Entomology, and Microbiology, Iowa State University, Ames, IA, United States

**Keywords:** nutrient mobilization, inorganic and organic, arbuscular mycorrhizal (AM) fungi/al, extraradical hyphae, nutrient cycling, hyphosphere, microbiome, networks

## Abstract

**Introduction:**

The hyphosphere of arbuscular mycorrhizal (AM) fungi is teeming with microbial life. Yet, the influence of nutrient availability or nutrient forms on the hyphosphere microbiomes is still poorly understood.

**Methods:**

Here, we examined how the microbial community (prokaryotic, fungal, protistan) was affected by the presence of the AM fungus *Rhizophagus irregularis* in the rhizosphere and the root-free zone, and how different nitrogen (N) and phosphorus (P) supplements into the root-free compartment influenced the communities.

**Results:**

The presence of AM fungus greatly affected microbial communities both in the rhizosphere and the root-free zone, with prokaryotic communities being affected the most. Protists were the only group of microbes whose richness and diversity were significantly reduced by the presence of the AM fungus. Our results showed that the type of nutrients AM fungi encounter in localized patches modulate the structure of hyphosphere microbial communities. In contrast we did not observe any effects of the AM fungus on (non-mycorrhizal) fungal community composition. Compared to the non-mycorrhizal control, the root-free zone with the AM fungus (i.e., the AM fungal hyphosphere) was enriched with *Alphaproteobacteria*, some micropredatory and copiotroph bacterial taxa (e.g., *Xanthomonadaceae* and *Bacteroidota*), and the poorly characterized and not yet cultured *Acidobacteriota* subgroup GP17, especially when phytate was added. Ammonia-oxidizing *Nitrosomonas* and nitrite-oxidizing *Nitrospira* were significantly suppressed in the presence of the AM fungus in the root-free compartment, especially upon addition of inorganic N. Co-occurrence network analyses revealed that microbial communities in the root-free compartment were complex and interconnected with more keystone species when AM fungus was present, especially when the root-free compartment was amended with phytate.

**Conclusion:**

Our study showed that the form of nutrients is an important driver of prokaryotic and eukaryotic community assembly in the AM fungal hyphosphere, despite the assumed presence of a stable and specific AM fungal hyphoplane microbiome. Predictable responses of specific microbial taxa will open the possibility of using them as co-inoculants with AM fungi, e.g., to improve crop performance.

## Introduction

Arbuscular mycorrhizal (AM) symbiosis is one of the prevailing symbiotic associations between plants and fungi on the planet, involving more than 70% of all vascular plant species (Brundrett and Tedersoo, 2018). The fungi are completely dependent on the supply of photosynthetic carbon (C) provided by their hosts to thrive and reproduce (Smith and Read, 2008). In return, AM fungi provide a wide range of benefits to the plant, including enhanced uptake of mineral nutrients such as phosphorus (P), nitrogen (N) and micronutrients (e.g., Zn and Cu) from soil to plant, by capitalizing on extensive extraradical (external) hyphal networks to explore a larger volume of soil than would be accessible to the roots (van der Heijden et al., 2008; Gao et al., 2021; Hui et al., 2022), facilitating water flow between soil and plant (Kikuchi et al., 2016; Delavaux et al., 2017; Püschel et al., 2021), and increasing resistance to various biotic (e.g., pathogens, grazing) and abiotic (e.g., salinity, drought, heavy metals) stresses (Smith and Smith, 2011; Faghihinia et al., 2020; Gao et al., 2020; Zai et al., 2021). AM fungi can also make connections with neighboring plants, redistributing the resources, benefits and costs, and thus stabilizing plant communities (Selosse et al., 2006; Jakobsen and Hammer, 2015; Faghihinia and Jansa, 2023), which may eventually increase plant community yield (Li et al., 2022).

Interestingly, AM fungi affect not only the host plant but also other soil microorganisms, especially bacteria, by creating an energy-rich microhabitat in the immediate vicinity of their external hyphae. This occurs through the release of hyphal exudates (metabolites and signaling molecules) (Qin et al., 2016; Lanfranco et al., 2017), facilitating microbial movement along the water film-coated hyphae in the soil matrix (Jansa and Hodge, 2021; Jiang et al., 2021), and facilitating contact between prey and predators (Rozmoš et al., 2022). In return, due to their limited exo-enzymatic repertoire (Tisserant et al., 2013), AM fungi are believed to selectively recruit beneficial bacteria (and/or archaea) on the surface of their extraradical hyphae, the “hyphoplane,” or in the soil affected by the hyphae, the “hyphosphere” (Artursson et al., 2006; Bonfante and Anca, 2009; Jansa and Hodge, 2021; Faghihinia et al., 2023). By stimulating metabolic activity of microbes to mineralize organic P and N (Nuccio et al., 2013; Zhang et al., 2014; Gryndler et al., 2018; Wang et al., 2019; Jiang et al., 2021), AM fungi are then able to acquire nutrients for their own use and that by their host plant. AM fungi may also compete with the soil microbes for soil mineral nutrients, e.g., NH_4_^+^, under deficient conditions, thereby altering microbial community structure (Nuccio et al., 2013; Gryndler et al., 2018; Veresoglou et al., 2019; Nuccio et al., 2022). However, whether and how AMF prime/suppress the activity of different microbes in the hyphosphere is clearly not yet fully understood.

Selective mechanisms underlying potentially preferential associations between AM fungi and microbial communities may depend on nutrient availability and the nutrient forms in the hyphosphere (Jansa and Hodge, 2021; Zhang et al., 2022; Faghihinia et al., 2023). Luthfiana et al. (2021) detected 141 different metabolite compounds in the hyphal exudates of *Rhizophagus clarus* and *R. irregularis* that changed differently among AM fungal species depending on the P supply. While primarily correlative, this finding provided insight into how AM fungal excretions and substrate nutrient availability might drive microbial community composition and their metabolic activities in the hyphosphere. Wang et al. (2019) observed significant changes in the bacterial community that harbored alkaline phosphatase within the hyphosphere of *Funneliformis mosseae,* which was attached to the leek root system. Such changes were in response to different forms of P (KH_2_PO_4_ or phytate), with a higher relative abundance of *Pseudomonas* in phytate-treated samples compared to those treated with KH_2_PO_4_ or the control samples (without any P amendment). Therefore, the interactions between AM fungi and soil microbes can be modulated based on the type of nutrient patches they encounter in soil. Yet, our current knowledge of the composition and functioning of the AM fungi-associated microbiomes and the outcome of the interactions on organic/inorganic nutrient utilization, as well as nutrient cycling in the hyphosphere is still very fragmentary.

The AM fungal hyphosphere has been shown to differ from the rhizosphere in terms of microbial community composition and function (Gahan and Schmalenberger, 2015; Zhang et al., 2018; Veresoglou et al., 2019; Zhou et al., 2020). Indeed, a taxonomically conserved and reproducible core microbiome is believed to exist in the hyphoplane/hyphosphere across various AM fungal species and environmental/soil factors (Emmett et al., 2021; Wang et al., 2022). However, until now, there has been a lack of extensive research dedicated to characterizing the AM fungi-associated microbiome, and research has primarily focused on bacteria as the predominant life form within the hyphosphere. Yet, there is evidence of complex interactions between AM fungi and other life forms and their potential role in soil nutrient cycling. For example, it has been demonstrated that the presence of a protist *Polysphondylium* sp. in a hyphosphere supplied with organic N in the form of chitin and inoculated with *Paenibacillus* sp., significantly increased the N uptake by AM fungus (Rozmoš et al., 2022). Protists play a major role in soil food webs by feeding on bacteria and fungi and thus recycling nutrients (Geisen et al., 2018; Guo et al., 2018). Therefore, the complex interactions between AM fungi and microbes need to be addressed at the community level. Moving from a single kingdom scale and bipartite interaction to inter-kingdom scale and multi-sided microbial associations by integrating all influential life forms such as protists and saprotrophic fungi is one further challenge required to comprehend complex interactions in the AM fungal hyphosphere.

Untangling this enigmatic zone of interaction and identifying the influential participants and underlying processes is needed, as the microbial communities of the hyphosphere obviously are critical for the uptake of organic nutrients by AM fungi (Hodge and Storer, 2015; Pii et al., 2015; Jansa et al., 2019), which subsequently could strongly affect plant nutrient status and health (Jeffries et al., 2003). Moreover, the impact of the hyphosphere is sufficient in magnitude to be considered significant in the context of global C, N, and P cycles and the resulting environmental impacts (Read and Perez-Moreno, 2003; Rillig, 2004; Cavagnaro et al., 2012). We hypothesized that soil microbial communities in the root-free soil are structured by presence or absence of AM fungi. In other words, the AM fungal “hyphosphere effect” would alter the community structure of various soil microbes and this would be further modulated by the type of nutrient supplement. Thus, a manipulative, fully factorial, and replicated experiment was carried out to (i) gain insights how the community of prokaryotes (bacteria and archaea), fungi, and protists changed due to the presence (M) or absence (NM) of arbuscular mycorrhizal fungus in a root- (referred to as the rhizosphere) and root-free (referred to as the hyphosphere in the presence of AM fungus) compartments; (ii) investigate whether and how the type of organic or inorganic nutrient supplement in the root-free compartment altered the structure of the microbiome; and to (iii) identify potential interactions between AM fungi and their associated microbes by co-occurrence network analysis to determine whether specific associations were influenced by the type of nutrients available to the hyphae/hyphosphere microbiome.

## Materials and methods

### Experimental design and potting substrate

**Figure 1:**
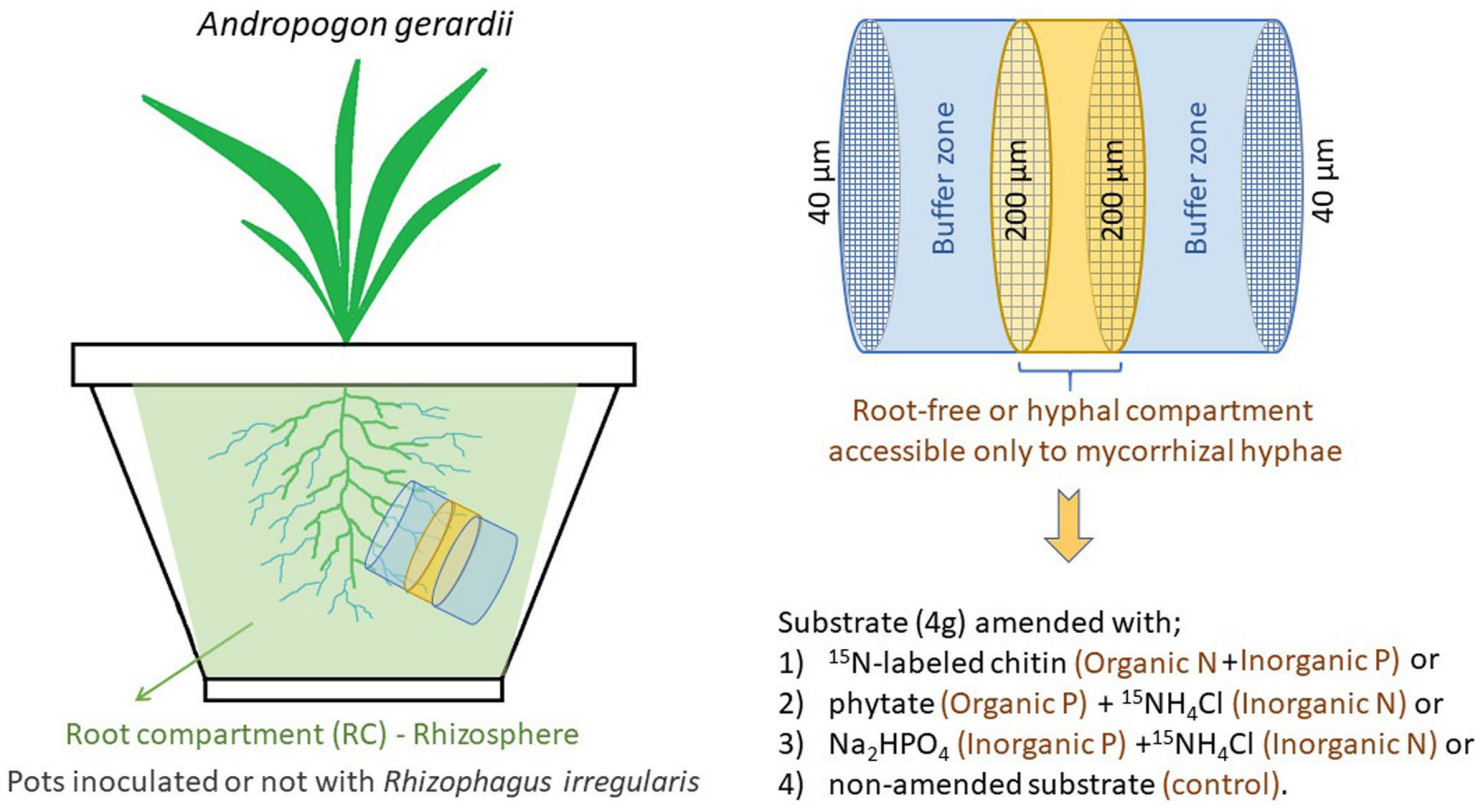
The schematic of the experimental design. The root-free compartment was supplemented with different combinations of organic and inorganic nutrients, with the total amount being the same in all supplemented compartments.

We used a completely randomized experimental design with two factors, inoculation with AM fungus, and the type of P and/or N amendment into the root-free compartment (Figure 1). Root-free compartments were amended with ^15^N-labeled chitin (organic N), phytate (organic P) + ^15^NH_4_Cl (inorganic N), Na_2_HPO_4_ (inorganic P) + ^15^NH_4_Cl (inorganic N), or nothing (control) (Figure 1). Each of the 8 treatment combinations were replicated 4 times, totaling 32 pots. We used 2 L tall pot [20 × 11 × 11 cm (height × width × depth)] filled with a previously described (Bukovská et al., 2016; Gryndler et al., 2018) sterilized soil-sand-zeolite mixture (10:45:45, v:v:v). That potting substrate was coarsely structured, slightly alkaline (pH = 8.9 in a water slurry 1:2.5, w:v) and nutrient-poor. It contained 46.5 mg kg^−1^ total P, of which 2.6 mg kg^−1^ was water-extractable (1:10 w:v, shaken for 20 h, and filtered through 0.2 μm nitrocellulose mixed ester filter), as well as 0.013 and 0.22% total N and organic C, respectively (Jansa et al., 2020).

To facilitate development of substrate microbiomes in the previously sterilized materials and to provide not only microbes adapted to this particular substrate conditions, but also those that might potentially be AM fungal hyphosphere specialists, the potting substrate was inoculated with a complex microbial community obtained from two sources. First a soil-zeolite-sand substrate (identical composition as above) planted in leeks 3.5 years ago but devoid of AM fungi and, second, the same substrate planted in leeks inoculated with *Rhizophagus irregularis* BEG 236 3 years ago. The substrates were mixed with sterile water (1,10, v,v) and the slurry devoid of AM fungi was sieved (500 μm steel mesh) to remove roots but to retain bacteria and fungi (as well as some micro-and meso-fauna), while the slurry containing AM fungus was filtered (5 μm Teflon filter) to remove all fungi and other microbes except prokaryotes. Each pot in the experiment described here received 20 mL of the inoculum obtained from substrate devoid of AM fungi and 10 mL of the inoculum obtained from substrate inoculated with AM fungus. The entire volume of the pot was accessible to the roots, with exception a small root-free compartment made of PVC cylinder (3.6 cm diameter × 3 cm length) filled with 40 g potting substrate that was covered with a 40 μm nylon filter (Figure 1 and Supplementary Figure S9). In the middle of the root-free compartment, 4 g of potting substrate amended with various types of P and/or N sources was sandwiched between 200 μm nylon filters (Figure 1). The root-free compartment was inserted approximately 8 cm below the surface at ~5-degree slant (Figure 1) to prevent accumulation of water in the compartment and to facilitate ingrowth of AM fungal hyphae.

### Arbuscular mycorrhizal fungus inoculation

The AM fungus *Rhizophagus irregularis* LPA9 (=BEG 236) was propagated *in vitro* in association with *Cichorium intybus* Ri-T-DNA transformed roots in compartmented bioreactors filled with MSR liquid medium as described by Řezanka et al. (2022) over a 6 month period. This was the same medium as described previously (i.e., Cranenbrouck et al., 2005; Dudáš et al., 2022), but without Phytagel, and with elevated P (81 μM). The roots were suspended over the liquid medium by a 40 μm nylon mesh, which allowed penetration of the fungal hyphae through the filter but not roots, to the MSR medium. In mycorrhizal (M) treatments, each pot received 50 mg fresh weight hyphae with approximately 20 thousand spores 3 cm below the seeding level. No AM fungal biomass was added to the non-mycorrhizal (NM) treatments.

### Organic and inorganic nutrient amendments to the root-free compartment

The root-free compartments were amended with organic or inorganic N, organic or inorganic P, or left unamended, as illustrated in Figure 1. Fully ^15^N labeled chitin extracted from *Zygorhynchus* sp. cell walls was prepared as described in Bukovská et al. (2018), which yielded an 84 μmol N and 177 μg P input. After adding 20 mg chitin powder to the pre-filled root-free compartment, 400 μL sterile deionized water was added to facilitate its incorporation. The phytate treatment included 1 mg of rice sodium phytate (Sigma), which provided 177 μg P, and 4.58 mg ^15^NH_4_Cl (corresponding to 84 μmol N). The inorganic N and P treatment comprised of 1.32 mg Na_2_HPO_4_ 12 H_2_O + 4.58 mg ^15^NH_4_Cl, providing the same amount of P and N per root-free compartment as the chitin and phytate treatments. In all cases, the amendments were mixed into the pre-filled root-free compartments and wetted with 400 μL sterile deionized water to facilitate its incorporation. The non-amended control was just added with 400 μL sterile deionized water.

### Timeline, environmental conditions, watering, and fertilization of the plants

The experiment was carried out between March and June 2021. All pots were sown with 50 *Andropogon gerardii* seeds supplied by Jelitto Staudensamen GmbH, Schwarmstedt, Germany. The seeds were added to the middle at a 5 mm depth and covered by the potting substrate. During the initial 10 days the pots were incubated in the dark at 23°C and 85% relative humidity to allow seed germination. Thereafter, the pots were moved to the experimental glasshouse at the Institute of Microbiology in Prague. Plants were grown for an additional 35 days under natural lighting supplemented with 500 W metal halide lamps, providing a minimum of 200 μmol photosynthetically active radiation (PAR) m^−2^ s^−1^ throughout the 14 h photoperiod (the peak illumination was reaching 800 μmol PAR m^−2^ s^−1^). Glasshouse temperature fluctuated between 18°C at night and 37°C during the warmest days. Pots were watered daily with deionized water to maintain water saturation of the substrate between 60 and 80% (checked gravimetrically). No fertilizers were provided.

### Sample collection and processing

Upon harvest, shoot and root biomass was harvested from each pot. The roots were washed with deionized water, an aliquot of the roots from each pot was transferred to 50% ethanol and stained with Trypan Blue in lactoglycerol according to the protocol by Koske and Gemma (1989). The extent of root colonization by AM fungal hyphae, arbuscules, and vesicles were assessed by the magnified intersection method described by McGonigle et al. (1990), observing 50 root intersections per sample. The remaining biomass samples were then dried at 65°C for 3 days for determining dry weights prior to pulverizing the samples with a MM200 ball mill (Retsch, Haan, Germany) at 25 Hz for 2 min. Representative samples of the potting substrate were collected from the root compartment (i.e., the rhizosphere, labeled T), buffer compartment (labeled B) and the root-free compartment (labeled N) from all pots. All samples were dried at 65°C for 3 days, milled as above, and further processed for elemental and molecular analyses (see below).

From M pots with chitin or phytate amendments, aliquots of the substrate collected from the root-free compartment were used to isolate culturable bacteria (using selective chitin and phytate media, respectively). To this end, 3 g of the root-free compartment was mixed with 40 mL sterile water and shaken vigorously by hand. The suspension was transferred to a sterile Petri dish and the hyphae or hyphal clumps were transferred with sterile forceps into 1,200 μL sterile water and homogenized with 4 glass beads (4 mm diameter) using the MM200 ball mill at 25 Hz for 45 s, twice. Fifty μL of the suspension was spread either on solid (0.5% phytagel) mineral MSR media devoid of mineral N and containing 0.5% (w:v) pulverized crab-shell chitin or MSR media devoid of mineral P and containing 8.9 mg sodium phytate +5 g glucose L^−1^ to isolate chitinolytic or phytate-mineralizing microbes, respectively, or incubated for 1 week in the same liquid media (without phytagel addition), upon shaking (60 rpm), before being spread on the solid media as above. Bacterial strains were then subcultured on Luria-Bertani broth, DNA extracted from their biomass using DNeasy PowerSoil Pro kit (Qiagen, Hilden, Germany), and 16S rRNA amplicons were generated using primers 27F-1098R, 534F-1521R, or 534F-1098R prior to sequencing both forward and reverse directions using BigDye™ Terminator v3.1 Cycle Sequencing.

### Elemental and isotopic analyses

Shoots and root P concentrations were measured spectrophotometrically using the Malachite Green method (Ohno and Zibilske, 1991) following incineration of 100 mg of dried and milled aliquots at 550°C for 12 h and extraction of the ashes with hot concentrated NHO_3_ as described previously (Püschel et al., 2017). The N and C concentrations, and the stable isotopic composition of N (i.e., the ^15^N/^14^N isotopic ratio) were measured on 2 mg aliquots of dried and milled plant samples or 20 mg aliquots of the potting substrates with a Flash 2000 CN analyzer coupled with a Delta V Advantage isotope ratio mass spectrometer via-ConFlo IV interface (ThermoFisher Scientific, Waltham MA, USA).

### Quantification of microbial guilds

DNA was extracted from 10 mg aliquots of pulverized root samples by the glass milk method (Gryndler et al., 2011), after adding 2 × 10^10^ copies of an internal DNA standard, ISC – a linearized plasmid containing a fragment of cassava mosaic virus DNA as described previously (Thonar et al., 2012) – to each sample before the extraction. The PowerSoil kit (Qiagen, Hilden, Germany) was used to extract DNA from 600 mg of pulverized potting substrate samples, after the ISC addition as above. Final elution was to 50 μL (TE buffer).

We used TaqMan quantitative PCR (qPCR) to quantify the ISC recovery, and *R. irregularis* by targeting its nuclear large ribosomal subunit (LSU) and mitochondrial LSU regions using marker systems (primers with hydrolysis probes) intra and mt5, respectively (Dudáš et al., 2022). Additionally, we also quantified total bacteria, fungi, and protist abundances as well as ammonia oxidizing bacteria (AOB) by using primers Eub, H, V4, and CTO, respectively, as detailed in Dudáš et al. (2022) and Additional File 6. We performed three independent (single-plex) qPCR assays per sample and marker system as described in detail in Dudáš et al. (2022). Results of the different qPCR assays were normalized for the DNA losses upon extraction by using the ISC recovery measured for each individual sample as described by Thonar et al. (2012). Each qPCR assay was first calibrated with the product of endpoint PCR performed with the corresponding primers on the DNA extracted from different substrate samples. The amplicons generated with identical primers were then pooled and purified (QIAquick PCR purification kit; Qiagen). The length of the resulting fragments was assessed was evaluated using electrophoresis on a 0.8% agarose gel, and DNA concentration in the amplicon samples was measured using the Quant-iT PicoGreen double-stranded-DNA (dsDNA) assay (Thermo Fisher Scientific, Waltham MA, USA) on a plate reader (Infinite 200 Pro; Tecan, Männedorf, Switzerland). Subsequently, dilution (5-fold and 10-fold) series were prepared from the amplicons, and the different dilustions were employed as templates for qPCR calibration, following a previously described method (Thonar et al., 2012). The qPCR quantification was conducted in 96-well plates, with a final reaction volume of 20 μL. Depending on whether the primer sets were designed in conjunction with TaqMan (hydrolysis) probes, which would be double labeled with fluorescein as a fluorophore and BHQ1 as a quencher, reaction mixtures were prepared using two master mixes. Specifically, we utilized the Luna universal probe qPCR master mix (M3004) for assays involving a probe and the Luna universal qPCR master mix (M3003) for those without a probe, both purchased from New England Biosciences (Ipswich, MA, USA). Fluorescence data were recorded in the SYBR green/fluorescein color channel. All analyses were carried out using the LightCycler 480 II instrument (Roche, Rotkreuz, Switzerland).

### NGS sequencing

Prokaryotic, fungal, and protistan community profiles were generated from the rhizosphere and root-free compartment samples. Amplicons for 16S rRNA V4 region of prokaryotes were produced using the 515-IL/806-IL primers and the V4 region of protists using the V4-IL primers as described previously (Dudáš et al., 2022) and the Additional File 6. Amplicons of fungi were generated using a semi-nested PCR approach using primers ITSOF (ACTTGGTCATTTAGAGGAAGT) and ITS4-IL (GTCTCGTGGGCTCGGAGATGTGTATAAGAGACAGNNNNNTCCTSCGCTTATTGATATGC) in triplicate in the first step (35 cycles, annealing at 48°C for 45 s) and primers gITS7-IL (TCGTCGGCAGCGTCAGATGTGTATAAGAGACAGNNNNNGTGARTCATCRARTYTTTG) and ITS4-IL in the second step (20 cycles, annealing at 52°C, 30 s). Thereafter, Nextera XT barcodes and sequencing adapters were added to the amplicons and then the library pool was purified by paramagnetic beads prior to sequencing on an Illumina 2×300 platform at the Joint Microbiome Facility (Vienna, Austria) as detailed previously (Dudáš et al., 2022) and in the Additional File 6.

Raw sequences were demultiplexed and adapter-trimmed, primers removed, quality filtered and clustered at 97% similarity levels in the Seed software (Větrovský et al., 2018) as described previously (Dudáš et al., 2022). Most abundant sequences from each cluster were then identified using the SILVA (prokaryotic and fungal) or PR2 (protistan) databases to identify target and non-target (i.e., contaminant) sequences. Contaminants (such as chloroplast and mitochondrial sequences within the prokaryotic dataset, and plant sequences within the protistan dataset) were removed, samples rarefied to equal sequencing depth, chimeras removed, and clustered at 97% similarity level to yield operational taxonomic units (OTUs) and the most abundant sequences per OTU were re-identified again. Relative abundances of the different microbial taxa (clumped at genus or higher, up to phylum, levels instead of 97% similarity levels) per sample were then used for subsequent statistical analyses. Raw sequencing data, quality filtered but otherwise unmodified, were deposited in the Sequence Read Archive operated by NCBI under accession number PRJNA977454.

### Statistical analysis

All data analyses were performed using R version 4.1.3 (R Core Team, 2021) unless otherwise noted. Two-way ANOVA was performed to determine the effects of mycorrhizal inoculum and nutrient amendments on plant variables including shoot, root, and total dry biomass, and P, N, and ^15^N contents in the biomass. The assumptions of ANOVA were verified by testing normality and homogeneity of variance for each variable.

The assumption of normality was assessed by examining the residuals of the ANOVA model and the QQ plot, as well as by conducting the Shapiro–Wilk test for each group level. Homogeneity of variance was evaluated by plotting the residuals against the fitted values and subsequently applying Levene’s test based on Crawley (2012). If there was a significant difference in the variances among the groups, data heteroscedasticity was addressed by employing a White adjustment within the ANOVA function. This adjustment incorporates a heteroscedasticity correction using a coefficient covariance matrix (White and Macdonald, 1980). Post-hoc multiple pairwise comparisons between groups were performed using the estimated marginal means and *p*-values were adjusted using Bonferroni correction. The analysis of variance and pairwise comparisons were conducted using the “rstatix” and “emmeans” packages, respectively. Plots were created using the “ggplot2” and “ggpubr” packages.

Taxa abundance at each taxonomic rank and α-and β-diversity indices were calculated using the “R microeco” package (v0.11.0) (Liu et al., 2020). Treatment effects on richness and Shannon’s diversity indices were tested by two-way ANOVA. Distance matrices were created using Bray–Curtis dissimilarity. Treatment effects on β-diversity were analyzed using non-parametric permutational multivariate analyses of variance (perMANOVA) tests with 1,000 permutations.

Linear discriminant analysis (LDA) effect size (LEfSe) (Segata et al., 2011) was used via the Galaxy/HutLab web application to identify differentially abundant taxa at various taxonomic levels (α-value for Kruskal-Wallis test among classes and for pairwise Wilcoxon test between subclasses = 0.05, threshold on the logarithmic LDA score for discriminative features = 2.0, strategy for multiclass analysis = all-against-all [more strict]).

To understand how microbial communities changed by the presence of the AM fungus under different nutrient treatments, we constructed co-occurrence networks using the SparCC accessed through the “SpiecEasi” package (Kurtz et al., 2015). Optimized correlation thresholds were selected using random matrix theory (RMT)-based method (Deng et al., 2012). Statistically significant (*p* < 0.01) SparCC correlations were included into the network analyses. Analyses were done at the genus level using the trans_network class of “R microeco” package (Liu et al., 2020). Network properties were measured using “R microeco” package. All network data were stored in the network.gexf file, and then visualized in Gephi (Bastian et al., 2009). To identify the potential role of taxa in the network, within-module (Zi) and among-module (Pi) connectivity was determined for each node (Deng et al., 2012). Nodes were categorized into four groups: (i) Module hubs, which are highly connected nodes within their respective modules (Zi > 2.5 and Pi ≤0.62); (ii) Connectors, which are nodes that link different modules (Zi ≤ 2.5 and Pi >0.62); (iii) Network hubs, which serve as both module hubs and connectors (Zi > 2.5 and Pi >0.62); and (iv) Peripherals, which are nodes with only a few connections, typically to other nodes within their own modules (Zi ≤ 2.5 and Pi <0.62).

## Results

### Effects of mycorrhizal inoculum and nutrient supplements on plants

As anticipated, inoculation with AM fungus significantly increased total and root dry biomass, shoot P concentration, root P concentration, total P content, and root N content (Table 1 and Supplementary Figure S1). Nutrient supplementation into the root-free compartment did significantly affect neither shoot or root biomass, nor their N or P contents (Table 1). However, ^15^N content in shoots and roots was significantly affected by mycorrhizal inoculation and the form of nutrient supplementation (Supplementary Figure S1). More ^15^N was transferred to the shoots of NM plants, whereas significantly more ^15^N was accumulated in the roots of M plants (Table 1 and Supplementary Figures S1, S2). Interestingly, in the presence of the AM fungus, more ^15^N originating from the root-free compartment was recovered in the buffer zone and rhizosphere than in the NM treatment (Supplementary Figure S2). Regardless of the mycorrhizal status of the plants, the most and least ^15^N was transferred to shoots when the root-free compartment was supplied with “PO_4_ + NH_4_” and “chitin,” respectively (Supplementary Figures S1, S2).

**Table 1:** Results of two-way ANOVA of the effects of mycorrhizal inoculum and nutrient supplement on plant variables.

	Mycorrhizal inoculum	Nutrient supplement	Mycorrhizal inoculum × Nutrient supplement
Shoot dry biomass	0.131 (0.721)	1.020 (0.401)	0.415 (0.744)
Root dry biomass	31.163 (**0.000**)	1.978 (0.144)	2.500 (0.084)
Total dry biomass	8.639 (**0.007**)	0.544 (0.657)	1.118 (0.361)
Shoot P concentration	817.62 (**0.000**)	2.770 (0.064)	2.580 (0.077)
Root P concentration	205.32 (**0.000**)	0.648 (0.592)	2.099 (0.127)
Total P content	370.05 (**0.000**)	1.252 (0.313)	2.306 (0.102)
Shoot N content	2.809 (0.107)	1.471 (0.247)	0.633 (0.601)
Root N content	21.69 (**0.000**)	0.759 (0.528)	2.073 (0.13)
Total N content	1.868 (0.184)	1.515 (0.236)	1.213 (0.326)
Shoot ^15^N (% transferred)	46.68 (**0.000**)	18.27 (**0.000**)	0.723 (0.499)
Root ^15^N (% transferred)	41.75 (**0.000**)	9.48 (**0.002**)	2.76 (0.09)
Total ^15^N (% transferred)	1.447 (0.245)	20.36 (**0.000**)	1.203 (0.323)

*F* and *p* values are indicated. Significant *p* values (≤0.05) are indicated in bold. Root ^15^N content was not significantly different between root-free compartment supplied with different nutrient supplements in the NM treatments. However, in M plants, the ^15^N content of the roots was significantly lower when the root-free compartment was amended with chitin than with the other nutrient supplements (Supplementary Figure S1).

### AM fungal abundance in rhizosphere and root-free compartments

Microscopic observations showed that the proportion of roots colonized by hyphae, vesicles, and arbuscules in the M treatment was not affected by the type of nutrient supplement (Supplementary Tables S1, S2), whereas the root colonization rates of the NM treatment remained close to zero (for hyphae) or at zero (for arbuscules and vesicles). Similarly, the abundance of AM fungus in the rhizosphere of the M treatment measured by qPCR was not affected by nutrient supply. However, the abundance of AM fungus in the root-free compartment in M treatment indicated a significant influence of the type of nutrients supplied (*p =* 0.034) (Supplementary Table S1), although post-hoc comparisons between multiple pairs showed no significant differences between nutrient amendments.

### Microbial abundance in rhizosphere and root-free compartments

**Figure 2:**
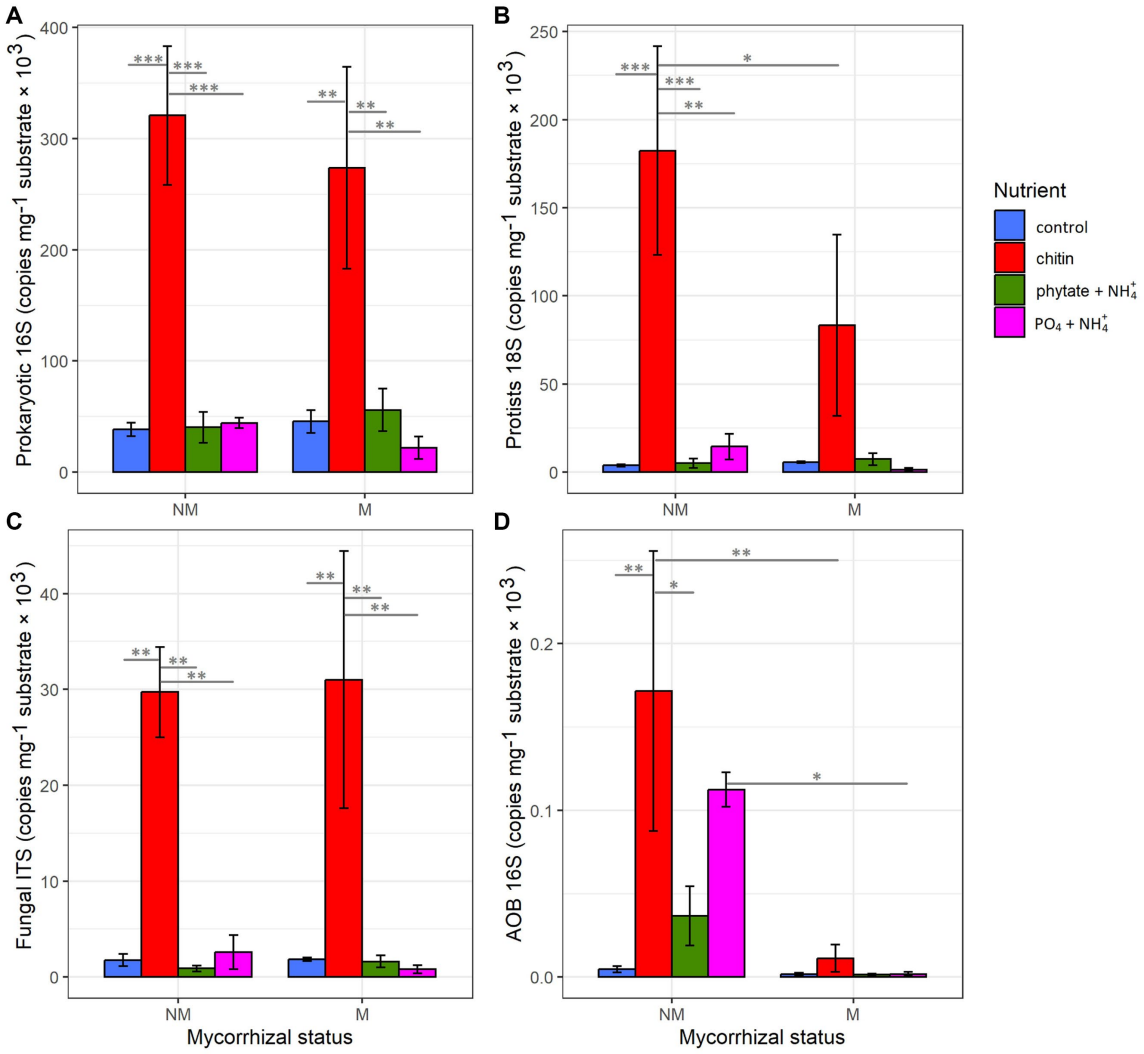
Influence of mycorrhizal inoculation and nutrient supplement in the root-free compartment on abundance of **(A)** prokaryotes, **(B)** protists, **(C)** fungi, and **(D)** AOB as determined by qPCR. Statistically significant differences are indicated by asterisks. Asterisks indicate levels of significance; *p* ≤ 0.05 (*), *p* ≤ 0.01 (**), *p* ≤ 0.001 (***), and *p* ≤ 0.0001 (****).

The presence of the AM fungus significantly affected the abundance of prokaryotes and fungi in the rhizosphere (Supplementary Table S3). Nutrient supplementation in the root-free compartment did not affect abundance of the different microbial guilds in the rhizosphere (Supplementary Table S3). Within the root-free compartment, the abundance of prokaryotes, protists, and fungi was significantly influenced by the type of nutrient supplement (Supplementary Table S3). The highest abundance was observed in the chitin-added compartment for all microbial groups (Figure 2).

Interestingly, mycorrhizal inoculation and the type of nutrient supplement into the root-free compartment significantly affected the abundance of AOB in the rhizosphere and hyphosphere (Supplementary Table S3 and Supplementary Figures S3, S4). Abundance of AOB was significantly suppressed in the root-free compartments in M compared to NM treatments, especially in the compartments supplied with “chitin” and “PO_4_ + NH_4_,” whereas the AOB were significantly enriched in rhizosphere in the presence of the AM fungus in “phytate+NH_4_” and “PO_4_ + NH_4_” treatments (Supplementary Figures S3, S4).

### Microbial diversity and richness in rhizosphere and root-free compartments

Regardless of mycorrhizal status and nutrient amendments, the rhizosphere always had significantly higher bacterial and fungal richness (the number of observed OTUs) and diversity (Shannon) compared to the root-free compartment. In the rhizosphere, nutrient treatment had no effect on the microbial diversity and richness (Supplementary Tables S4, S5). AM fungal inoculation increased bacterial richness, especially in phytate+NH_4_ treatment (*p* = 0.009). In addition, Shannon diversity of protists was significantly affected by the presence of the AM fungus in the rhizosphere, especially in the PO_4_ + NH_4_ treatment (*p* = 0.027) (Supplementary Tables S4, S5).

In the root-free compartment, the richness and diversity of all microbial groups (except fungi) were influenced by nutrient treatment, with “chitin” exhibiting the lowest diversity (Supplementary Tables S4, S5). Shannon diversity of prokaryotes was significantly affected by nutrient additions and the interaction of nutrient treatment and mycorrhizal inoculation in the root-free compartment. Significantly higher diversity in M compared to NM chitin (*p* = 0.001) and PO_4_ + NH_4_ (*p* = 0.039) amended root-free compartments were observed (Supplementary Table S4).

### Rhizosphere and root-free compartment microbial community structure

**Figure 3:**
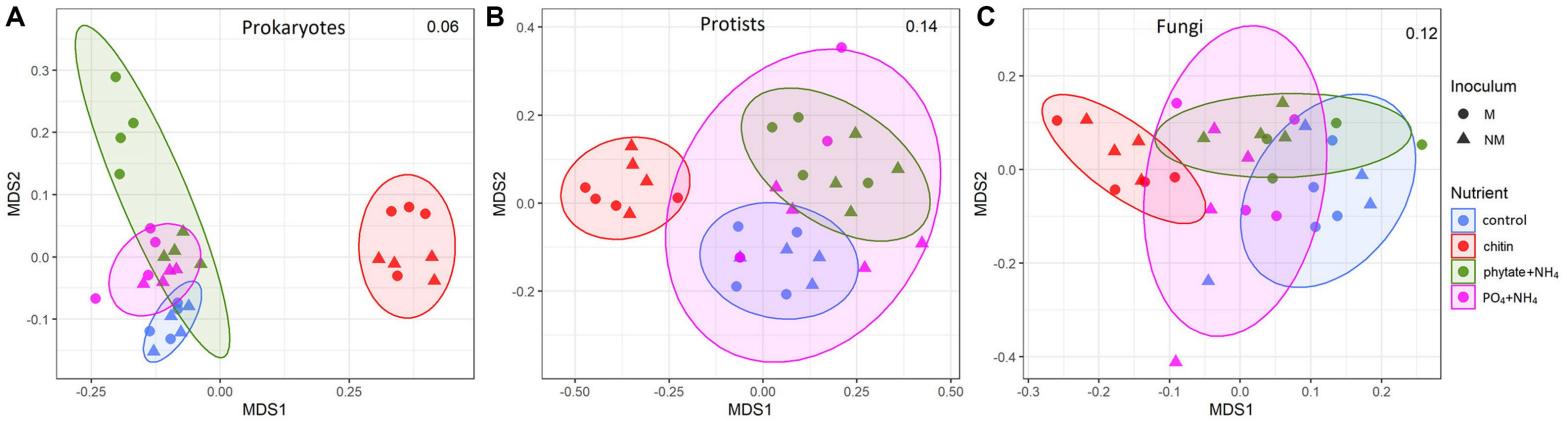
Nonmetric multidimensional scaling (NMDS) ordinations of Bray-Curtis dissimilarities of microbial communities (A) Prokaryotes, (B) Protists, (C) Fungi in the root-free compartment at the operational taxonomic unit level in mycorrhizal (M) and non-mycorrhizal (NM) pots with different nutrient supplements. Ellipses indicate normal distributions of each treatment group. Stress values are indicated in the upper right corner of each plot.

AM fungal inoculation and nutrient supplements into the root-free compartment influenced community structure of the microorganisms in the root-free and rhizosphere zones differently, with some significant interactions (Table 2). In the root-free compartment, the prokaryotic community and to a lesser extent the protistan community were affected by AM fungal inoculation (Table 2 and Figure 3). All microbial groups were affected by the type of nutrient supplement in the root-free compartment, especially in the chitin-amended treatment (Figure 3). The interaction between mycorrhizal inoculum and nutrient supplement was significant for prokaryotes in both the rhizosphere and root-free compartments suggesting that the effect of mycorrhiza on prokaryotic community composition depended on the form of available nutrients.

**Table 2:** Permutational analysis of variance of β-diversity.

	Mycorrhizal inoculum	Nutrient supplement	Mycorrhizal inoculum × Nutrient supplement
Root-free compartment			
Prokaryotes	0.063 (**0.004**)	0.579 (**0.001**)	0.093 (**0.002**)
Protists	0.034 (0.058)	0.526 (**0.001**)	0.06 (0.203)
Fungi	0.029 (0.222)	0.387 (**0.001**)	0.072 (0.330)
Rhizosphere			
Prokaryotes	0.181 (**0.001**)	0.089 (0.198)	0.114 (**0.039**)
Protists	0.185 (**0.001**)	0.081 (0.419)	0.069 (0.656)
Fungi	0.096 (**0.001**)	0.101 (0.206)	0.122 (0.059)

*R*^2^ and *p*-values are indicated. Number of permutations: 999. Significant *p* values (≤0.05) are indicated in bold.

### Microbial community profiling in rhizosphere and root-free compartments

In the root-free compartment, 3,192, 28, 553, and 611 unique bacterial, archaeal, protistan, and fungal OTUs were identified by sequencing the V4 region of the 16S rRNA gene of both bacteria and archaea, the V4 region of the 18S rRNA gene of protists, and ITS2 region of fungi, respectively (Supplementary Figure S5). The bacterial phyla with highest relative abundance were *Actinomycetota* (36%), *Pseudomonadota* (35%) and *Bacillota* (8%) with different characteristics depending on presence of the AM fungus and the nutrient amendment. About 9% of the prokaryotes in the root-free compartment were unknown, i.e., could not be identified to the genus level by using the bioinformatics pipeline (Supplementary Figure S6). Among the protists, *Amoebozoa* (46%) and *Rhizaria* (39%) were the predominant phyla in the root-free compartment (Supplementary Figure S6). Of the total number of OTUs of prokaryotes, protists, and fungi, 86.8, 72.2, and 82% of OTUs were shared between M and NM treatments, respectively.

In the rhizosphere, 3,496, 28, 526, and 749 unique prokaryotic, archaeal, protistan, and fungal OTUs were identified, respectively (Supplementary Figure S7). The relatively most abundant bacterial phyla were *Pseudomonadota* (26%), *Actinomycetota* (24%), *Bacillota* (19%), and *Acidobacteriota* (7%) with about 10% of OTUs not possible to be identified at the genus level (Supplementary Figure S8). In terms of protists, the rhizosphere was dominated by *Amoebozoa* (38%), *Alveolata* (36%), and *Rhizaria* (18%) (Supplementary Figure S8). *Thaumarchaeota* and *Ascomycota* were the most abundant archaeal and fungal phyla in both the rhizosphere (89, 98%) and root-free compartments (85, 93%), respectively.

**Figure 5:**
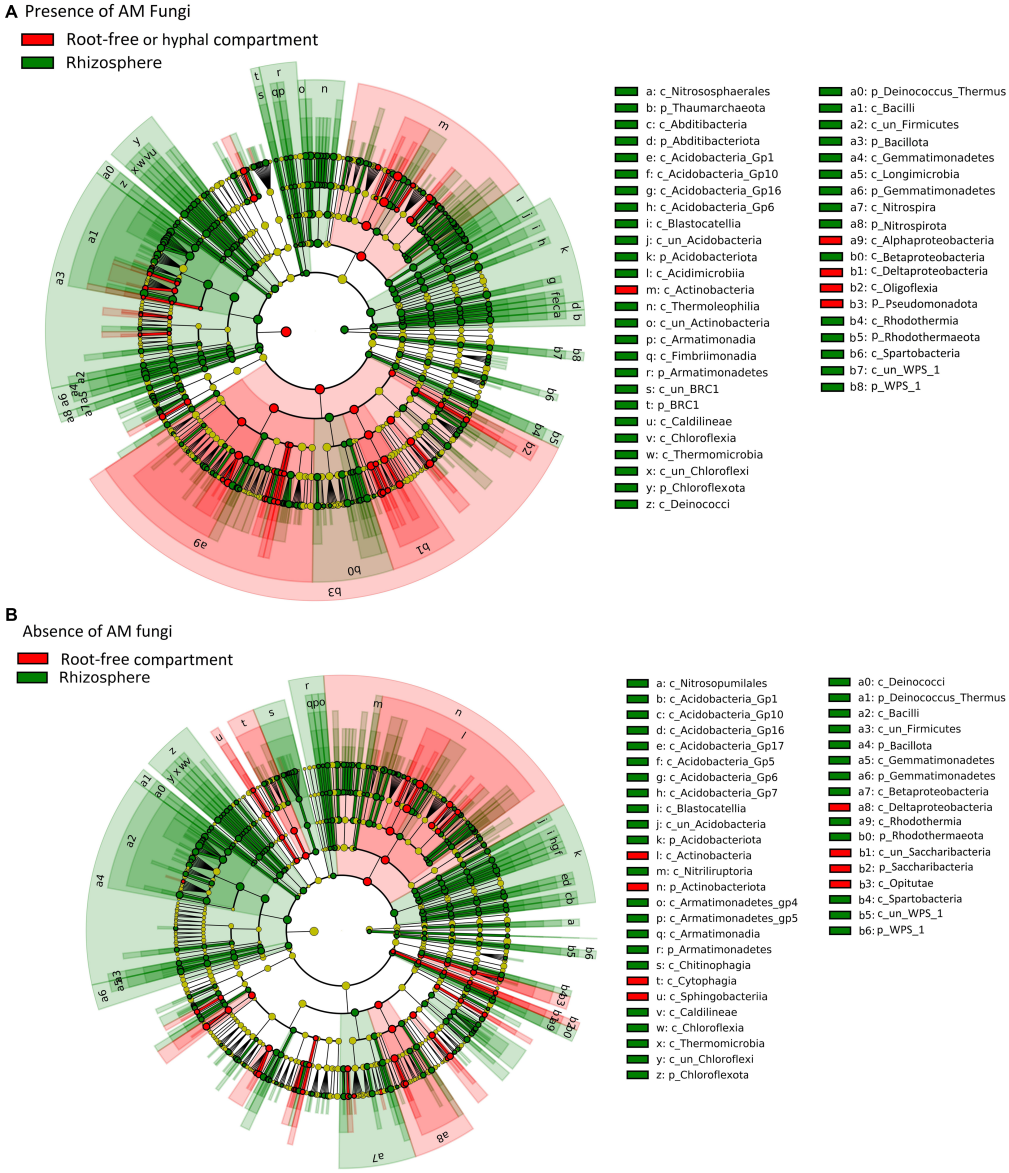
Linear discriminant analysis (LDA) effect size cladograms (LEfSe; Kruskal–Wallis (*p* < 0.05); Pairwise Wilcoxon (*p* < 0.05); logarithmic LDA score > 2.0) highlighting the prokaryotic biomarkers that differ statistically in terms of abundance between the rhizosphere and the root-free compartments in presence (A) or absence (B) of the AM fungus. The nodes radiated inside to outside represent phylogenetic levels from kingdom to genus. Taxonomic rank designations are given before the names of the microbes: “p_; c_; o_; f_; g_” stands for phylum, class, order, family or genus. The letters and numbers in the cladograms refer to the respective prokaryotic names, which can be found in the keys to the right of each cladogram. The biomarkers are represented by colored nodes and shading (red and green). The species that do not show significant differences are colored yellow.

### Differential abundance of microbial taxa in rhizosphere and root-free compartments

**Figure 4:**
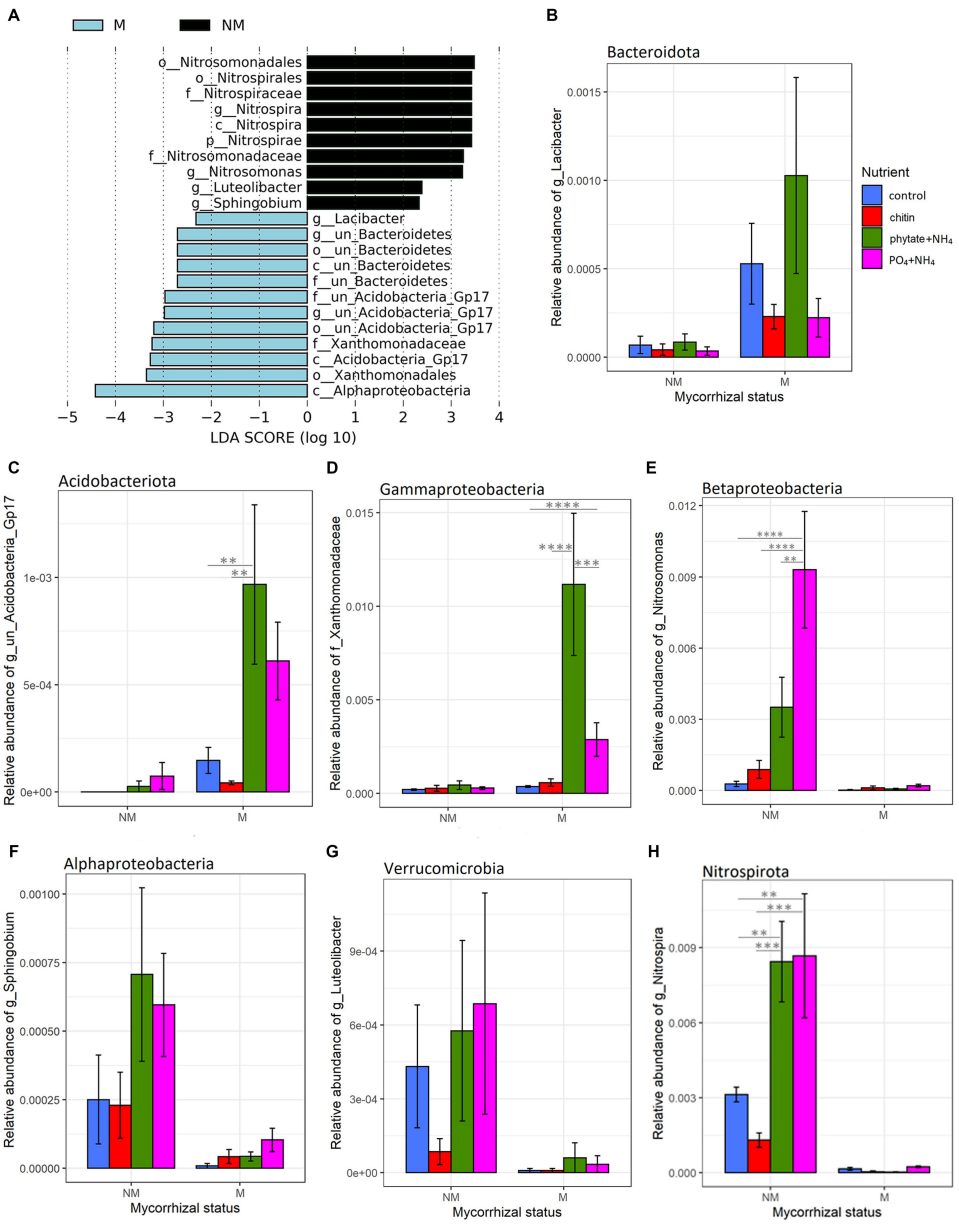
Effect of mycorrhizal inoculation and nutrient amendment into the root-free compartment on bacterial relative abundance. (A) List of differentially abundant bacterial taxa between mycorrhizal (M) and non-mycorrhizal (NM) treatments in the root-free compartment, ranked by effect size. (B–H) Differentially (M vs. NM) abundant bacterial taxa in the root-free compartment under different nutrient amendments (B: Bacteroidota, C: Acidobacteriota, D: Gammaproteobacteria, E: Betaproteobacteria, F: Alphaproteobacteria, G: Verrucomicrobia, H: Nitrospirota). Asterisks indicate levels of significance of comparison between nutrient amendment treatments withing the respective mycorrhizal inoculation treatment; *p* ≤ 0.05 (*), *p* ≤ 0.01 (**), *p* ≤ 0.001 (***) and *p* ≤ 0.0001 (****).

Linear discriminant effect size analyses (LEfSe) revealed that mycorrhizal treatments strongly influenced prokaryotic communities in the root-free compartment. Presence of AM fungus enriched *Bacteroidota, Alphaproteobacteria, Acidobacteriota* subgroup GP17, and *Gammaproteobacteria* (*Xanthomonadaceae*) (Figure 4). In contrast, in the NM treatments, we observed enrichment of *Nitrospirota*, *Nitrosomonas* and *Sphingobium (Pseudomonadota)*, as well as *Luteolibacter* (*Verrucomicrobiota*) (Figure 4). While prokaryotic communities were affected by mycorrhizal inoculation, we did not observe any differentially abundant protists and only one fungal family, *Psathyrellaceae* (*Basidiomycota*) depleted in the root-free compartment due to presence of the AM fungus.

We also examined the differential abundance of taxa in the root-free compartment compared to the rhizosphere in the presence/absence of the AM fungus. While there were substantial similarities in the rhizosphere and root-free microbiomes in pots as depends on the presence or absence of *R. irregularis*, there also were some intriguing differences. For example, compared to the respective rhizosphere samples, there was significant (*p* < 0.05) enrichment of taxa from *Pseudomonadota*, particularly the *Alphaproteobacteria* and *Oligoflexia* in the M compared to the NM root-free compartments (Figure 5). Interestingly, the rhizospheres of M plants were enriched in *Nitrospirota/Nitrospira* and *Thaumarcheota* illustrating the influence of AM fungi, possibly because of their ability to acquire N for the plant, in shaping the rhizosphere nitrifying bacterial community structure (Figure 5). This observation complements results of the qPCR on the AOB in the rhizosphere and the influence of nutrient amendments in the root-free compartment on the rhizosphere of plants with AM fungi (Supplementary Figure S3 and Supplementary Table S3).

### Microbial co-occurrence networks

**Figure 6:**
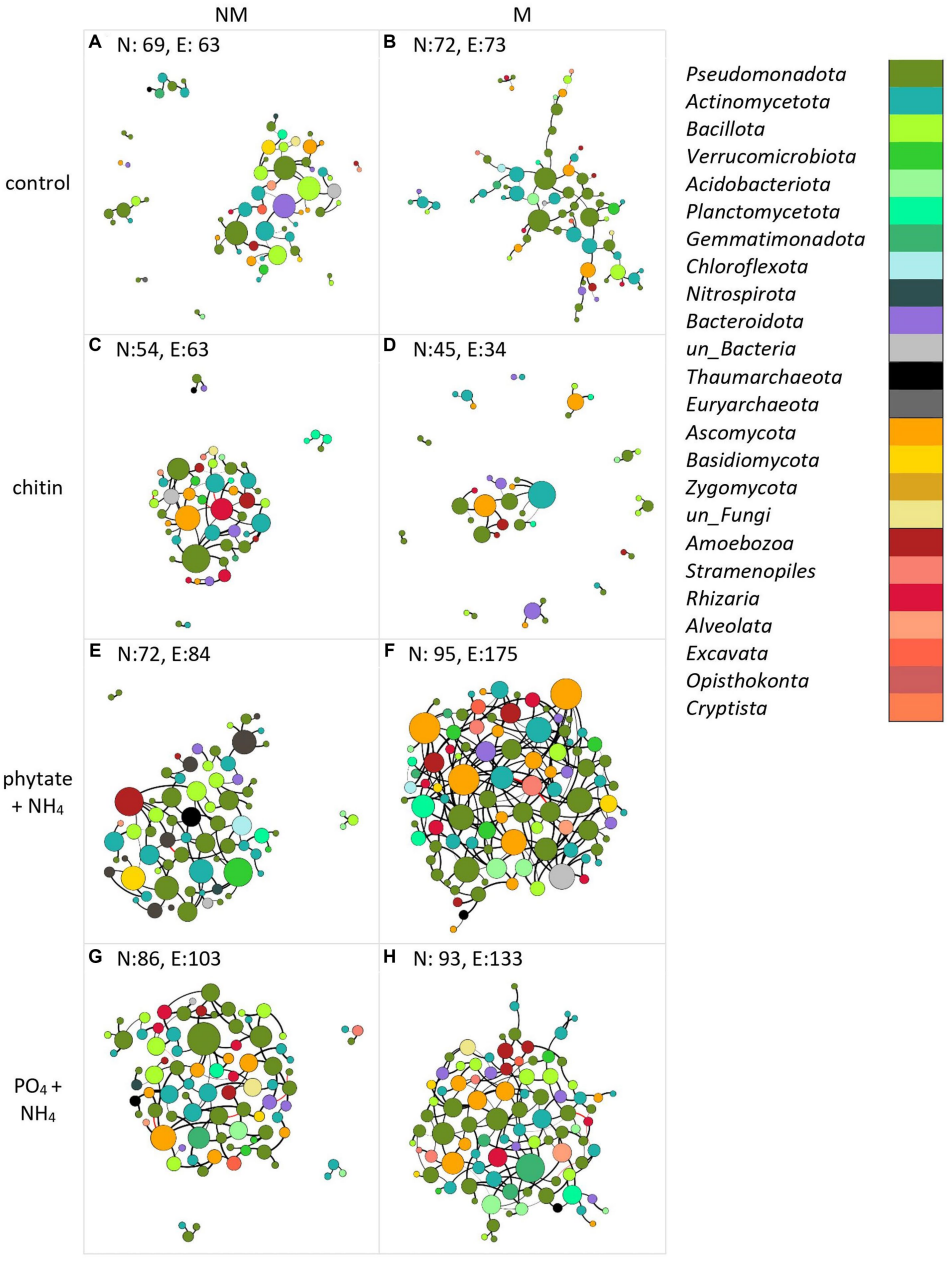
Co-occurrence networks of bacteria (purple-green), archaea (gray-black), protists (different shades of red), and fungi (yellow-brown) in the absence (A,C,E,G) or presence (B,D,F,H) of the AM fungus (*Rhizophagus irregularis*) under different nutrient amendment into the root-free compartment (A,B) unamended control, (C,D) amended with chitin, (E,F) amended with phytate and NH_4_Cl, (G,H) amended with Na_2_HPO_4_ and NH_4_Cl). Each network was constructed using data from four independent pots. The number of nodes (N) and edges (E) are indicated in each panel. The size of the nodes is proportional to the degree of centrality. Edges are colored by interaction type; positive (black) and negative (red) correlations. Edge thickness reflects the strength of the association. Interdependencies among taxa were determined by SparCC (Sparse Correlations for Compositional data).

**Figure 7:**
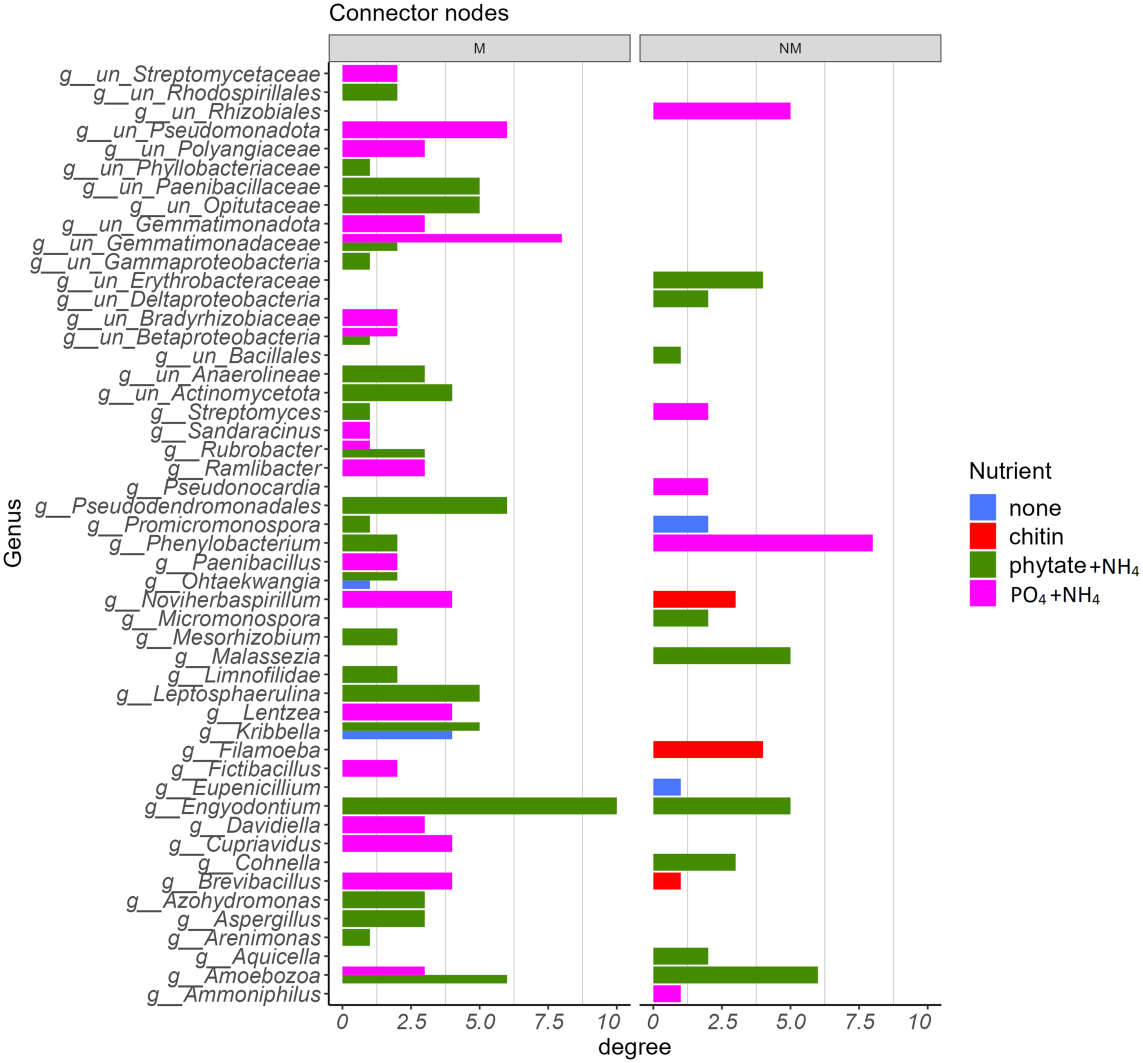
Taxonomic identity of bacterial, protistan, and fungal connector nodes and their associated degree centrality in the absence (NM) or presence (M) of AM fungus *Rhizophagus irregularis* parsed by the type of nutrient amendment in the root-free compartment.

Co-occurrence networks of prokaryotes, protists, and fungi in the root-free compartment were constructed for mycorrhizal and non-mycorrhizal treatments for each type of nutrient amendment. Analysis of the topological properties revealed that regardless of the nutrient amendments into the root-free compartment, except for the chitin amendment, the number of nodes and edges, average degree, average clustering coefficient, and number of keystone species were higher in the M than in the NM treatment (Figure 6 and Supplementary Table S6). In the root-free compartments amended with phytate+NH_4_ or PO_4_ + NH_4_, networks were more interconnected in the presence of AM fungus while not exhibiting an increase in modularity (Figure 6 and Supplementary Table S6). Moreover, the number of connectors and module nodes increased in the presence of the AM fungus, especially when the root-free compartment was amended with “phytate+NH_4_” or “PO_4_ + NH_4_” (Supplementary Table S6). In the presence of the AM fungus, microbial networks were composed of more diverse taxonomic groups (Figure 7), independent of the type of nutrient amendment into the root-free compartment. Consistent with Figure 7, we isolated bacteria from AM fungal mycelium in chitin and phytate compartments, obtaining *Pseudomonas*, *Streptomyces*, *Enterobacter* and *Kribella* spp. Regardless of the type of nutrient-amendment, *Pseudomonadota*, *Actinobacteria*, *Bacillota*, *Amoebozoa*, and *Ascomycota* were identified as to play a major role in the AM fungal hyphosphere microbial networks (Supplementary Table S7).

## Discussion

**Figure 8:**
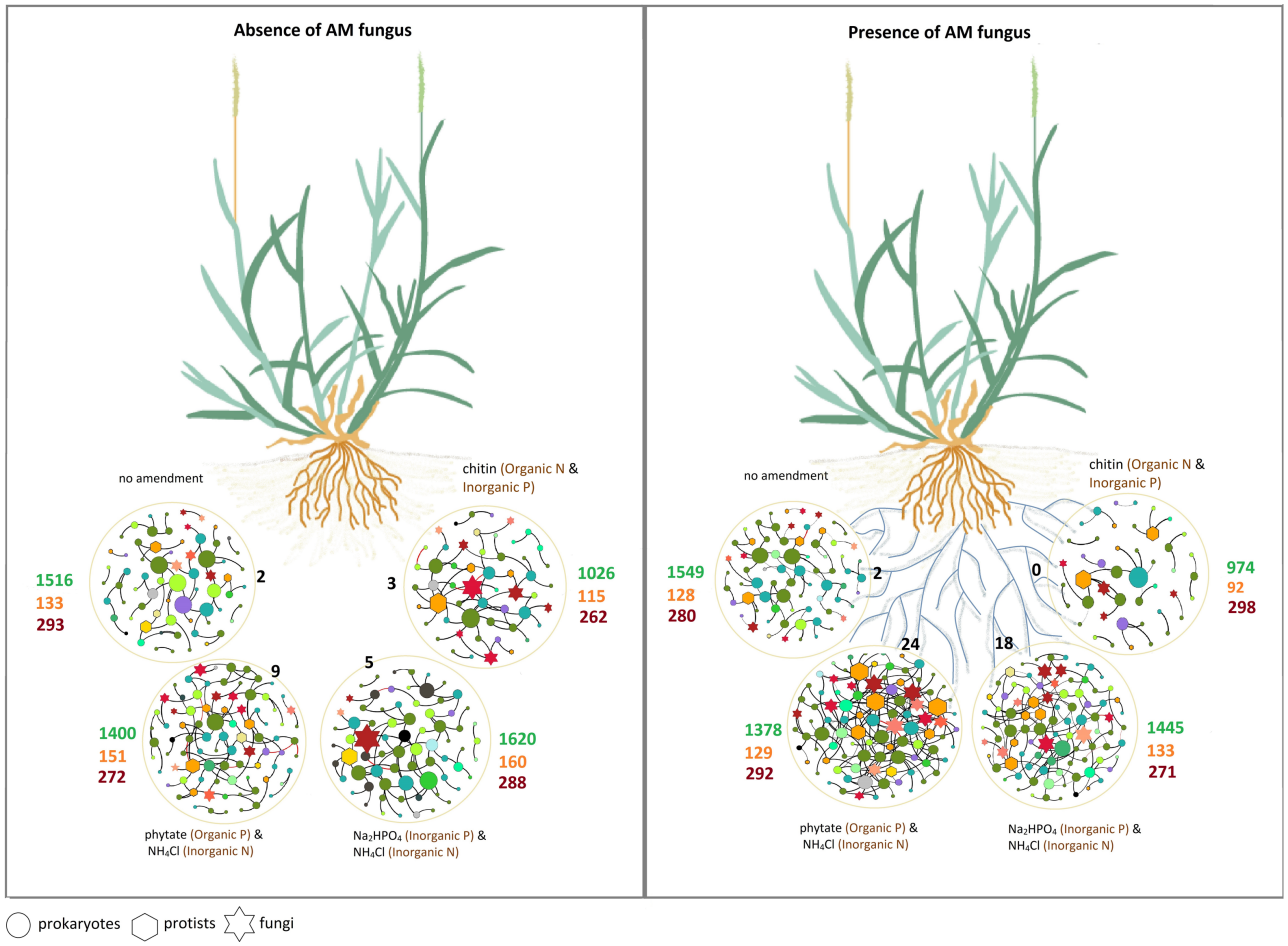
The schematic of the main results of the study illustrating the effects of the hyphosphere of the AM fungus *Rhizophagus irregularis*, modulated by the type of nutrient supplement, on prokaryotic, fungal, and protist communities. Circles represent spatially separated patches that contain different sources of organic and inorganic nutrients. The different microbial groups in the co-occurrence networks are shown with different shapes. The size of the nodes is proportional to the degree of centrality. The color of the nodes represents different microbial groups at phylum level (see Figure 6). Black numbers indicate the number of keystone taxa (connector nodes) in each network (see the list of keystone taxa in Additional File 1, Supplementary Table S7). Green numbers indicate the richness (i.e., number of OTU) of prokaryotes, orange numbers the richness of protists and dark red numbers the richness of fungi (more details in Supplementary Table S5).

The study of associations between microorganisms in the AM fungal hyphosphere can serve as a basis to uncover multitrophic interactions between AM fungi and a variety of other microbial taxa. Here, we investigated the interactions between AM fungus and various soil microbial groups under different nutrient amendments into a compartment that only the AM fungal hyphae could access but not the plant roots. Our findings support our hypothesis that microbial interactions in the AM fungal hyphosphere are further modulated by/dependent on the form of available nutrients (Figure 8).

### AM fungus promoted root growth and N and P accumulation in the root system

Consistent with previous studies with the same plant species (Gryndler et al., 2018; Bukovská et al., 2021), AM fungal-inoculated plants exhibited significantly greater shoot and root biomass, especially when the root-free compartment was amended with organic P. Additionally, root N content was significantly higher in all M plants regardless of the type of nutrient amendment into the root-free compartment. These results, combined with the observations of significantly higher ^15^N content in roots and lower ^15^N content in shoots of M plants (Supplementary Figures S1, S2), suggest that the AM fungus promoted root growth and accumulation of more N and P in the roots (at least partly immobilizing these resources in mycorrhizal fungal structures). Indeed, it is well known that establishment of mycorrhizal symbiosis can promote plant growth and the uptake of P and N (Govindarajulu et al., 2005; Smith and Read, 2008; Hodge and Fitter, 2010). Similarly, Wattenburger et al. (2020) indicated that AM fungal-proficient maize plants had higher NO3-and total N contents compared to AM fungal-deficient plants, suggesting that M plants were better able to take up N from the soil, probably directly through extraradical hyphal absorption and indirectly by promoting plant root growth/architecture. Indeed, the identification of a gene encoding an AM fungus-inducible ammonium transporter, ZmAMT3;1, in maize (*Zea mays*) roots indicate there is a direct pathway for the transfer of N from AM fungi to plants (Hui et al., 2022).

### The prokaryotic, protistan, and fungal communities responded differently to the presence of the AM fungus

Our data show that the presence of AM fungus did not affect the abundance, richness and diversity of prokaryotes in the root-free compartment with different nutrient amendments. However, the bacterial community composition (beta diversity) was significantly affected by the presence of the AM fungus in the root-free compartment. Indeed, AM fungal extraradical hyphae release exudates comprising diverse compounds that can be detected and/or utilized by soil microbes, particularly prokaryotes, and stimulate them to move toward the hyphosphere (Jansa and Hodge, 2021; Jiang et al., 2021). Likewise, through modulating (decreasing) available nutrient concentrations and possibly also through producing certain compounds or attracting microbial antagonists, the AM fungi could also suppress abundance of other microbes in the hyphosphere (see below for more details).

Protists were the only microbial guild in our study whose richness and diversity were significantly reduced in the presence of the AM fungus. These results somewhat contradict findings in other studies suggesting a positive effect of protists on the AM fungal function through the release of N from consumed bacterial biomass in a mechanism termed the “microbial loop” (Koller et al., 2013; Rozmoš et al., 2022). However, the responses of protists in our study may be a consequence of differences in protist dietary niche breadth or changes in prey availability caused by the AM fungal activity. As most protists selectively feed on bacteria (Amacker et al., 2022), changes in the hyphosphere bacterial community composition mediated by AM fungi is likely to also alter protist communities. Theoretically, protists with distinct prey preferences and narrower dietary niche width could dominate in the presence of AM fungi to feed on the hyphosphere bacterial community, leading to more deterministic shifts in the protist community. In contrast, in the absence of AM fungi, protists with few prey preferences could feed on a broader breadth of bacterial species. We were unable to identify many of the protists at lower taxonomic levels, but our results showed that two protist species from *Amoebozoa* and *Rhizaria* were more prevalent in the NM treatment. Similarly, to our observations in the root-free compartment, AM fungal inoculation also reduced protistan diversity in the rhizosphere. A potential explanation for this observation is that AM fungi can reduce rhizosphere carboxylate exudation, thus altering the availability of substrates for bacteria and, hence, their consumers (Ryan et al., 2012). Clearly, protists play an important, but still rather poorly understood role in shaping microbial communities and soil processes that warrant more research. Surprisingly, we did not observe a similar response of mycorrhizal inoculation on the overall fungal (non-mycorrhizal) community composition, possibly because prokaryotes responded more rapidly to hyphal exudates than fungi or because fungi had a greater spatial extent due to their hyphal morphology, thus integrating larger soil zones than just the hyphosphere.

### Type of soil nutrients affect composition of microbes in the root-free zone

The type of nutrient amendments significantly affected the abundance of prokaryotes, fungi, and protists in the root-free compartment (Supplementary Table S3 and Figure 2). Consistent with previous work (Bukovská et al., 2018), chitin-amendment resulted in higher abundances of bacteria, fungi, and protists (Figure 2) compared to other nutrient treatments. In contrast, chitin caused significantly lower richness and diversity of all microbial groups compared to the other nutrient treatments (Supplementary Tables S4, S5). This could be explained by the strong enrichment of specialists in chitin-amended compartments (Mariadassou et al., 2015; Xu et al., 2020), which have a local advantage over generalists. Previous studies have shown that micro-habitats are consistently dominated by specialists, resulting in a robust and positive correlation between abundance and specificity (Mariadassou et al., 2015). Accordingly, our data show that chitin-amended compartments are highly enriched in the bacteria *Archangium* (*Myxococcales*) and *Phenylobacterium* (*Caulobacterales*), especially in the absence of AM fungus; these genera have the potential to be involved in chitin degradation (Sharma and Subramanian, 2017; Yurgel et al., 2022).

We observed that protist abundance, richness, Shannon diversity index, and community composition were all affected by the form of nutrient supplied. Protistan communities are known to be predominantly structured by abiotic factors (Krashevska et al., 2014; Guo et al., 2018; Zhao et al., 2019). For example, moderate amounts of N and P amendments alter the biomass and community structure of testate amoebae, the most important and abundant protists in acidic forest ecosystems and montane rainforests (Krashevska et al., 2014). The responses by testate amoebae to these nutrients were complex since they benefited from the addition of N but the presence of P had adverse effects on them, with the addition of P negating the positive effect of N (Krashevska et al., 2014).

In the chitin-amended zones, in both the M and NM treatments, several protist species were significantly enriched, including *Cercomonas* (*Rhizaria*), *Vermamoeba* and *Filamoeba* (*Amoebozoa*). *Cercomonas* and amoeboid protists are common predators of bacteria in soil, and their enrichment in the chitin-amended root-free compartments may be a response to increased prey availability (Amacker et al., 2022). Interestingly, we found *Filamoeba* as a key microbial taxon in the microbial network upon absence of the AM fungus (Figure 7). The (non-mycorrhizal) fungal community in the root-free compartments was also influenced by the type of nutrient supplement, which was anticipated since fungi are drivers of soil organic matter decomposition and the availability of inorganic and organic nutrients influence these processes (López-Mondéjar et al., 2018).

### Microbial taxa affected by presence of the AM fungus

Differential abundance analyses revealed little influence of the AM fungus presence on protist and fungal communities, but a strong influence on prokaryotic community structure in the root-free compartment. The samples obtained from the M treatment were enriched in *Acidobacteriota* (sub group GP17), *Alphaproteobacteria, Gammaproteobacteria* (primarily *Xanthomonadaceae*) and *Bacteroidota,* particularly when phytate was provided as a source of organic P (Figure 4). These findings are consistent with earlier results, where Emmett et al. (2021) observed greater enrichment of *Alpha-and Gamma-proteobacteria* in the bacterial communities associated with extraradical mycelium of *R. irregularis* in different soils under greenhouse conditions. Wang et al. (2022) also found significant enrichment of *Alpha-and Gamma-proteobacteria* in the AM fungal hyphosphere across contrasting climatic zones, from humid to arid, in a field experiment. Interestingly, there was strong enrichment of unclassified members of the *Bacteroidota* as well as the genus *Lacibacter* in the M compared to NM treatments (Figure 4). It is conceivable that *Bacteroidota* are enriched in the AM fungal hyphosphere due to the decomposition of complex soil organic matter provided by AM fungal exudates (Li et al., 2017). It is also plausible that the energy-rich hyphal exudates of AM fungi support copiotrophic bacterial taxa with relatively rapid growth rates with high N demand such as *Bacteroidota* (Lueders et al., 2006). Lastly, the AM hyphal exudates may stimulate bacterial growth that, in turn, stimulates growth of predatory *Bacteroidota* capable of actively lyse living bacterial prey, which is crucial for the turnover of biomass carbon and other easily degradable organic material in soil ecosystems (Cederlund et al., 2014).

We found significant enrichment of the *Xanthomonadaceae* in the presence of the AM fungus, especially in root-free compartments amended with “phytate+NH_4_.” Like the *Bacteroidota*, many *Xanthomonadaceae* (*Gammaproteobacteria*) have been classified as micropredatory, copiotrophic bacteria (Lueders et al., 2006; Fierer et al., 2012). Interestingly, some unclassified members of the *Xanthomonadaceae* increased significantly with long-term addition of mixed organic–inorganic fertilizers as compared to just inorganic fertilizers (Li et al., 2017). In addition, Wang et al. (2022) found a positive association between *Xanthomonadales* and phosphatase activity in the hyphosphere of AM fungi under *in situ* and greenhouse conditions, suggesting a possible role for members of this taxa in promoting the utilization of organic P by AM fungi. However, in contrast to our results, other studies have reported that *Xanthomonadaceae* were prevalent in AM fungi-suppressing soils (Svenningsen et al., 2018; Cruz-Paredes et al., 2019). Since there are many species within the family *Xanthomonadaceae* and we have not identified differentially abundant taxa at species or generic levels, it was not possible to infer their functional traits in the AM fungal hyphosphere in this study.

We also observed a significant enrichment of *Acidobacteriota* subgroup GP17 in the M compared to the NM treatments, especially upon the “phytate+NH_4_” or “PO_4_ + NH_4_” amendments. While the metabolic functions of *Acidobacteria* subgroups are not well established, there is evidence for a positive correlation between the abundance of the *Acidobacteria* subgroup Gp17 and soil availability of nutrients such as N and P (Navarrete et al., 2015; de Chaves et al., 2019; Chen et al., 2021). Interestingly, *Acidobacteria* subgroup Gp17 likely lack genes for the breakdown of cellulose, hemicellulose, starch, pectin, and chitin or participation in the glyoxalate cycle, suggesting that interactions with other microorganisms are necessary for obtaining the C sources required for their growth (de Chaves et al., 2019).

Taken together, consistent enrichment of some bacteria in the AM fungal hyphosphere could indicate the existence of a core AM fungal microbiome. The energy-rich resources provided by AM fungal hyphae could facilitate recruitment and maintenance of specific bacterial species in the hyphosphere. In return, bacteria could provide beneficial services to the AM fungus, such as degrading complex organic matter and releasing P and N in the vicinity of hyphae, strengthening the defense mechanisms of AM fungi, or inhibiting their pathogens (Faghihinia et al., 2022). In addition, AM fungal hyphae may also facilitate contact between prey and predators, which could explain the enrichment of predatory taxa in the AM fungal hyphosphere.

### The AM fungus significantly suppressed abundance of nitrifying bacteria in its hyphosphere

A surprising feature of the M treatment was the significant suppression of AOB *Nitrosomonas* sp. and nitrite-oxidizing bacterium *Nitrospira* sp. in the hyphosphere, based on two lines of evidence, our differential abundance (Figure 4) and qPCR (Figure 2) analyses. Suppression of AOB in the presence of AM fungi has recently been reported (Gryndler et al., 2018; Dudáš et al., 2022) in some, but not all, studies. For example, in a pot experiment consisting of spatially discrete patches with various organic N forms and *Andropogon gerardii* as host plant, Bukovská et al. (2018) showed significant suppression of microbial abundances, particularly AOB, in the presence of *R. irregularis* hyphae networks. Considering that AM fungi have a high N demand, that ammonium is probably the preferred N source for the AM fungi (Johansen et al., 1993; Lanfranco et al., 2011; Ngwene et al., 2013), the most likely mechanism is competition for available NH_4_^+^ (Nuccio et al., 2013; Veresoglou et al., 2019). Because heterotrophic microbes are relatively stronger competitors for NH_4_^+^ compared to ammonia oxidizers (Verhagen and Laanbroek, 1991), AM fungi might be able to outcompete ammonia oxidizers for the available NH_4_^+^ pool and therefore have an inhibitory effect on their growth (Wattenburger et al., 2020). This competition for ammonium ions could also explain observations that AM fungi suppress N_2_O losses from soil (Bowles et al., 2018; Storer et al., 2018; Qiu et al., 2022). Moreover, this could explain suppression of nitrite-oxidizing bacteria since nitrite production by ammonia-oxidizers is hindered. While this is a plausible explanation, there also are many reports indicating that AM fungi have positive or neutral effects on the abundance or metabolic activity of ammonia oxidizers (Chen et al., 2013; Teutscherova et al., 2019; Dudáš et al., 2022). These discrepancies could be a consequence of differing experimental contexts influencing ammonia oxidizer growth (Avrahami et al., 2003; Nicol et al., 2008; Veresoglou et al., 2012; Stempfhuber et al., 2015; Thion et al., 2016) and that mycorrhizal symbiosis and nitrification are typically studied separately (Chai and Schachtman, 2022). Unlike in the AM fungal hyphosphere, we observed enrichment of *Nitrosomonas*, nitrite-oxidizing *Nitrospirota*, and ammonia oxidizing *Thaumarchaeota* taxa in the rhizosphere (Figure 5), suggesting that ammonia production is higher in the rhizosphere than in the AM fungal hyphosphere and thereby permitting better growth of nitrifiers. Indeed, uncovering relationships between AM fungi and nitrifiers is of great importance to better understand nitrification and the soil N cycle and how to better manage agroecosystems to reduce N leaching and nitrous oxide production (Beeckman et al., 2018).

### Network complexity increases in the presence of AM fungus

Inclusion of a P source together with ammonium ions into the root-free compartment increased microbial network complexity, particularly when AM fungus was present. In contrast, inclusion of chitin as an N-source in the root-free compartment resulted in a more modular, less interconnected network upon presence of the AM fungus. The simplicity of the network in the chitin-amended compartments is consistent with the notion that microbial communities with more specialists (e.g., chitin utilizers) would exhibit simpler co-occurrence patterns, with fewer links in the network, than those with more generalists, such as those that might be present in unamended compartments (Liu et al., 2022). The potential stabilization of commensal interactions between these microbes and mycorrhizal fungi in the presence of organic N is plausible, but specific hypotheses need still to be formulated and tested, for example using *in vitro* experimental setups and synthetic microbial communities in the future.

Consistent with how nutrient amendments into the root-free compartment influenced network structure (Figure 6), there was little similarity in the species identity of network connector nodes between modules (Figure 7). Most connector nodes were represented by members of the *Pseudomonadota*, *Actinobacteria*, *Bacillota*, and *Ascomycota*. The presence of *Ascomycota* as connecting nodes in our networks, especially in the “phytate+NH_4_” treatment, is not surprising, as this group of fungi is a central component of the soil food webs and extremely important for the degradation/turnover of soil organic matter (Challacombe et al., 2019).

## Conclusion

Our results provide evidence that the type of nutrients available in the “local” hyphosphere environment strongly shapes the composition of microbial communities associated with AM fungi. Thus, the microbiota colonizing the AM fungal hyphosphere are not stochastically assembled from the surrounding soil, but their composition is also strongly affected by the form of nutrients present in their microenvironment, such as those they encounter in patchily distributed nutrients in soil. The structure of the *R. irregularis* hyphosphere microbial community is thus strongly influenced by the type of nutrient it encounters, which influences associations with other microbes in the community as well as potential ecosystem processes (e.g., nitrification). Overall, our study showed that the form of (locally) available nutrient is one of the main drivers for microbial abundance and community composition in AM fungal hyphosphere suggesting that the AM fungal core microbiome is likely dynamically responding to environmental conditions. Whether this has important implications for knowledge-based future agricultural practices needs to be further explored.

## Data availability statement

The datasets presented in this study can be found in online repositories. The names of the repository/repositories and accession number(s) can be found in the article/Supplementary material.

## Author contributions

MF: Data curation, Formal analysis, Investigation, Validation, Visualization, Writing – original draft, Writing – review & editing. LH: Writing – review & editing. HH: Data curation, Methodology, Writing – review & editing. PB: Data curation, Formal analysis, Methodology, Writing – review & editing. MR: Data curation, Methodology, Writing – review & editing. MK: Data curation, Methodology, Writing – review & editing. JJ: Conceptualization, Funding acquisition, Investigation, Methodology, Project administration, Resources, Supervision, Writing – review & editing.
