# Supplementary material for: Nutrient-dependent cross-kingdom interactions in the hyphosphere of an arbuscular mycorrhizal fungus
Source: Front Microbiol. 2024 Jan 4;14:1284648. doi: 10.3389/fmicb.2023.1284648 (PMC10794670; doi:10.3389/fmicb.2023.1284648)
Supplement: Supplementary file 1 [file Data_Sheet_1.zip › Supplementary Material 3.DOCX]

# Bacteria in Hyphal compartment

#same codes were used for protists and fungi
library(ggplot2)
library(microeco)
library(devtools)

library (SpiecEasi)
library(ggpubr)
library(magrittr)
library(file2meco)
library(RJSONIO)
library(WGCNA)

library(MASS)

library(GUniFrac)

library(randomForest)

library(ggdendro)
library(ggrepel)
library(agricolae)

library(gridExtra)

library(picante)

library(pheatmap)
library(tidytree)

library(igraph)

library(readr)
library("rgexf")
library("ggraph")
library(igraph)
library(igraphdata)
library(dplyr)

setdiff, setequal, union

library("GGally")

library("ggnetwork")
library("statnet")

setwd ("A:\\ ")


otu_table_Bacteria_N<-read.table("otu_table_Bacteria_Nsoil.txt", sep = '\t' )

taxonomy_table_Bacteria_N<-read.table("taxonomy_table_Bacteria.txt", sep = '\t' )

sample_info_Bacteria_N<-read.table("sample_info_Nsoil.txt", header = T, sep = '\t' )


View(otu_table_Bacteria_N)
View(taxonomy_table_Bacteria_N)
View(sample_info_Bacteria_N)


rownames(otu_table_Bacteria_N) <- paste0("OTU", 1:nrow(otu_table_Bacteria_N))
colnames(otu_table_Bacteria_N)<- sample_info_Bacteria_N$SampleID
View(otu_table_Bacteria_N)


rownames(taxonomy_table_Bacteria_N) <- rownames(otu_table_Bacteria_N)
colnames(taxonomy_table_Bacteria_N) <- c("Kingdom", "Phylum", "Class", "Order", "Family", "Genus", "Species")
View(taxonomy_table_Bacteria_N)


rownames(sample_info_Bacteria_N)<- colnames(otu_table_Bacteria_N)
View(sample_info_Bacteria_N)


#Make sure that the data types are all data.frame

class(otu_table_Bacteria_N)

## [1] "data.frame"

class(taxonomy_table_Bacteria_N)

## [1] "data.frame"

class(sample_info_Bacteria_N)

## [1] "data.frame"

#Take a look at data

otu_table_Bacteria_N[1:4, 1:5]

## N1 N2 N3 N4 N5
## OTU1 592176 479391 650473 626473 916029
## OTU2 72634 120547 68318 57833 866159
## OTU3 26667 16895 20392 11443 1922
## OTU4 1245 933 932 1443 1281

taxonomy_table_Bacteria_N[1:5, 1:6]

## Kingdom Phylum Class Order
## OTU1 Bacteria Actinobacteria Actinobacteria Propionibacteriales
## OTU2 Bacteria Proteobacteria Deltaproteobacteria Myxococcales
## OTU3 Bacteria Proteobacteria Alphaproteobacteria Sphingomonadales
## OTU4 Bacteria Proteobacteria Betaproteobacteria Burkholderiales
## OTU5 Bacteria un_Bacteria un_Bacteria un_Bacteria
## Family Genus
## OTU1 Kribbellaceae Kribbella
## OTU2 Cystobacteraceae Archangium
## OTU3 Sphingomonadaceae Sphingomonas
## OTU4 Oxalobacteraceae Noviherbaspirillum
## OTU5 un_Bacteria un_Bacteria

sample_info_Bacteria_N[1:5, ]

## SampleID Compartment Inoculum Nutrient
## N1 N1 Nsoil NM none
## N2 N2 Nsoil NM none
## N3 N3 Nsoil NM none
## N4 N4 Nsoil NM none
## N5 N5 Nsoil NM chitin

#Generally, users' taxonomic table may have some chaotic information,
#such as NA, unidentified and unknown. These information can potentially
#influence the following taxonomic abundance calculation and other
#taxonomy-based analysis. So it is usually necessary to clean this data using
#the tidy_taxonomy function. Another very important result of this operation is
#to unify the taxonomic prefix automatically, e.g. transforming D_1__ to p__
#for phylum level or adding p__ to phylum directly.

# make the taxonomic information unified, very important

taxonomy_table_Bacteria_N %<>% tidy_taxonomy

# Let's create a microtable object with more information
dataset_Bacteria_N <- microtable$new(sample_table = sample_info_Bacteria_N, otu_table = otu_table_Bacteria_N, tax_table = taxonomy_table_Bacteria_N)

## 371 taxa are removed from the otu_table, as the abundance is 0 ...

dataset_Bacteria_N

## microtable-class object:
## sample_table have 32 rows and 4 columns
## otu_table have 3222 rows and 32 columns
## tax_table have 3593 rows and 7 columns

class(dataset_Bacteria_N)

## [1] "microtable" "R6"

#we remove OTUs which are not assigned in the Kingdom "k__Archaea" or "k__Bacteria".

dataset_Bacteria_N$tax_table %<>% base::subset(Kingdom == "k__Archaea" | Kingdom == "k__Bacteria")
dataset_Bacteria_N

## microtable-class object:
## sample_table have 32 rows and 4 columns
## otu_table have 3222 rows and 32 columns
## tax_table have 3591 rows and 7 columns

#We also remove OTUs with the taxonomic assignments "mitochondria" or "chloroplast."
dataset_Bacteria_N$filter_pollution(taxa = c("mitochondria", "chloroplast"))

## Total 0 taxa are removed from tax_table ...

print(dataset_Bacteria_N)

## microtable-class object:
## sample_table have 32 rows and 4 columns
## otu_table have 3222 rows and 32 columns
## tax_table have 3591 rows and 7 columns

#To make the OTU and sample information consistent across all files in the dataset object,
#we use function tidy_dataset to trim the dataset.

dataset_Bacteria_N$tidy_dataset()
print(dataset_Bacteria_N)

## microtable-class object:
## sample_table have 32 rows and 4 columns
## otu_table have 3220 rows and 32 columns
## tax_table have 3220 rows and 7 columns

#Then let's use sample_sums() to check the sequence numbers in each sample.

dataset_Bacteria_N$sample_sums() %>% range

## [1] 2999508 3000350

#Let's calculate the taxa abundance at each taxonomic rank

dataset_Bacteria_N$cal_abund()

## The result is stored in object$taxa_abund ...

class(dataset_Bacteria_N$taxa_abund)

## [1] "list"

# show part of the relative abundance at Phylum level
dataset_Bacteria_N$taxa_abund$Phylum[1:5, 1:5]

## N1 N2 N3 N4
## k__Bacteria|p__Actinobacteria 0.33970933 0.31983983 0.38811398 0.38574930
## k__Bacteria|p__Proteobacteria 0.30748727 0.27741634 0.27565902 0.28786794
## k__Bacteria|p__un_Bacteria 0.13063810 0.15883609 0.12432191 0.10288244
## k__Bacteria|p__Firmicutes 0.06994206 0.09547119 0.07412573 0.08597538
## k__Bacteria|p__Bacteroidetes 0.04153840 0.04488279 0.04588868 0.04295486
## N5
## k__Bacteria|p__Actinobacteria 0.40901167
## k__Bacteria|p__Proteobacteria 0.48790924
## k__Bacteria|p__un_Bacteria 0.02350045
## k__Bacteria|p__Firmicutes 0.03411056
## k__Bacteria|p__Bacteroidetes 0.01616469

#The function save_abund() can be used to save the taxa abundance file to a local place easily.

dataset_Bacteria_N$save_abund(dirpath = "taxa_abundance_bacteria_Nsoil")

#Then, let's calculate the alpha diversity.

# If you want to add Faith's phylogenetic diversity, use PD = TRUE, this will be a little slow
dataset_Bacteria_N$cal_alphadiv(PD = FALSE)

## The result is stored in object$alpha_diversity ...

# return dataset$alpha_diversity
class(dataset_Bacteria_N$alpha_diversity)

## [1] "data.frame"

# save dataset$alpha_diversity to a directory
dataset_Bacteria_N$save_alphadiv(dirpath = "alpha_diversity_bacteria_Nsoil")


#Let's go on to beta diversity with function cal_betadiv().
#We provide four most frequently used indexes: Bray-curtis, Jaccard, weighted Unifrac and unweighted unifrac.


# If you do not want to calculate unifrac metrics, use unifrac = FALSE
# require GUniFrac package installed
dataset_Bacteria_N$cal_betadiv(unifrac = FALSE)

## The result is stored in object$beta_diversity ...

# return dataset$beta_diversity
class(dataset_Bacteria_N$beta_diversity)

## [1] "list"

# save dataset$beta_diversity to a directory
dataset_Bacteria_N$save_betadiv(dirpath = "beta_diversity_bacteria_Nsoil")


#composition information of communities

# create trans_abund object
# use ? Phyla with the highest abundance in the dataset.

#Alpha diversity can be transformed and plotted using trans_alpha class.
#Creating the object of trans_alpha class can invoke the alpha_diversity data stored in the microtable object.

#t1 <- trans_alpha$new(dataset = dataset_Bacteria_N, group = "Nutrient")

#t1$alpha_stat[1:5, ]

#Then, we test the differences among groups

#t1$cal_diff(method ="anova")

# return t1$res_alpha_diff
#t1$res_alpha_diff[1:5, ]

# we first create an trans_beta object
# measure parameter can invoke the distance matrix of bray in dataset$beta_diversity

t1 <- trans_beta$new(dataset = dataset_Bacteria_N, group = "Nutrient", measure = "bray")


# use PCoA as an example, PCA or NMDS is also available
t1$cal_ordination(ordination = "NMDS")

## Run 0 stress 0.06398063
## Run 1 stress 0.06398067
## ... Procrustes: rmse 5.324565e-05 max resid 0.0001816856
## ... Similar to previous best
## Run 2 stress 0.06398063
## ... Procrustes: rmse 7.67489e-06 max resid 2.242645e-05
## ... Similar to previous best
## Run 3 stress 0.06398063
## ... Procrustes: rmse 9.668279e-06 max resid 3.21595e-05
## ... Similar to previous best
## Run 4 stress 0.07023465
## Run 5 stress 0.06398063
## ... Procrustes: rmse 1.394727e-05 max resid 4.737171e-05
## ... Similar to previous best
## Run 6 stress 0.06398063
## ... Procrustes: rmse 8.085358e-06 max resid 2.6108e-05
## ... Similar to previous best
## Run 7 stress 0.06398063
## ... Procrustes: rmse 1.830047e-05 max resid 6.227048e-05
## ... Similar to previous best
## Run 8 stress 0.06398063
## ... Procrustes: rmse 1.167221e-05 max resid 3.957526e-05
## ... Similar to previous best
## Run 9 stress 0.06398065
## ... Procrustes: rmse 4.567961e-05 max resid 0.0001553014
## ... Similar to previous best
## Run 10 stress 0.06398063
## ... Procrustes: rmse 1.012097e-05 max resid 3.12105e-05
## ... Similar to previous best
## Run 11 stress 0.06398065
## ... Procrustes: rmse 3.49666e-05 max resid 0.0001174768
## ... Similar to previous best
## Run 12 stress 0.06398063
## ... Procrustes: rmse 8.430623e-06 max resid 2.473139e-05
## ... Similar to previous best
## Run 13 stress 0.06398063
## ... Procrustes: rmse 1.097399e-05 max resid 3.629014e-05
## ... Similar to previous best
## Run 14 stress 0.06398063
## ... Procrustes: rmse 2.190971e-05 max resid 7.454829e-05
## ... Similar to previous best
## Run 15 stress 0.06398063
## ... Procrustes: rmse 6.656745e-06 max resid 2.051253e-05
## ... Similar to previous best
## Run 16 stress 0.06398063
## ... Procrustes: rmse 1.746177e-05 max resid 5.940552e-05
## ... Similar to previous best
## Run 17 stress 0.06398063
## ... Procrustes: rmse 9.935593e-06 max resid 3.350833e-05
## ... Similar to previous best
## Run 18 stress 0.06398063
## ... Procrustes: rmse 6.794441e-06 max resid 2.076183e-05
## ... Similar to previous best
## Run 19 stress 0.06398063
## ... New best solution
## ... Procrustes: rmse 6.00271e-06 max resid 1.90268e-05
## ... Similar to previous best
## Run 20 stress 0.06398063
## ... Procrustes: rmse 3.362599e-06 max resid 9.78692e-06
## ... Similar to previous best
## *** Best solution repeated 2 times

## The ordination result is stored in object$res_ordination ...

# t1$res_ordination is the ordination result list
class(t1$res_ordination)

## [1] "list"

# plot the PCoA result

tiff("Bacteria.Nsoil.nmds.tiff", width = 6, height = 4, units = 'in', res = 300)


t1$plot_ordination(plot_color = "Nutrient", plot_shape = "Inoculum",
 plot_type = c("point", "ellipse"),
 plot_group_order=c ("none","chitin","phytate_NH4Cl","mineral_NP"),
 color_values =c ("royalblue1","red","chartreuse4","magenta1")
)

dev.off()

## png
## 2

#Clustering plot is also a frequently used method.

#t1$plot_clustering(group = "Nutrient", replace_name =c("Nutrient", "Inoculum"))


#perMANOVA(Anderson 2001) is often used in the differential test of distances among groups.


# manova for specified group set: here "Group + Type"
t1$cal_manova(cal_manova_set = "Nutrient * Inoculum")

## The result is stored in object$res_manova ...

t1$res_manova$aov.tab

## NULL

#PERMDISP(Anderson et al. 2011) is also implemented to check multivariate
#homogeneity of groups dispersions (variances).

# PERMDISP for the whole comparison and for each paired groups
t1$cal_betadisper()

## The result is stored in object$res_betadisper ...

t1$res_betadisper

##
## Permutation test for homogeneity of multivariate dispersions
## Permutation: free
## Number of permutations: 999
##
## Response: Distances
## Df Sum Sq Mean Sq F N.Perm Pr(>F)
## Groups 3 0.039782 0.0132607 5.7678 999 0.006 **
## Residuals 28 0.064375 0.0022991
## ---
## Signif. codes: 0 '***' 0.001 '**' 0.01 '*' 0.05 '.' 0.1 ' ' 1
##
## Pairwise comparisons:
## (Observed p-value below diagonal, permuted p-value above diagonal)
## chitin mineral_NP none phytate_NH4Cl
## chitin 4.0300e-01 5.7400e-01 0.009
## mineral_NP 3.9182e-01 9.6000e-02 0.034
## none 5.7268e-01 9.3120e-02 0.001
## phytate_NH4Cl 8.3973e-03 4.1449e-02 8.1721e-05

#For the explanation of statistical methods in microbial ecology,
#please read http://mb3is.megx.net/gustame

# #ANOVA

options(knitr.duplicate.label = "allow")


library(wesanderson)
library(tidyverse)

library(ggpubr)
library(rstatix)

library(emmeans)
library(ggplot2)
library(Rmisc)

library(plyr)
library(dplyr)
library(magrittr)

setwd ("A:\\ ")

P60.data<-read.table("P60.txt", header = T, sep = '\t' )

names(P60.data)

## [1] "No" "Nutrient" "Inoculum"
## [4] "Shoot.DW" "Root.DW" "Total.DW"
## [7] "Shoot.P" "Root.P" "Plant.total.P"
## [10] "Shoot.N" "Root.N" "Plant.total.N"
## [13] "Shoot.15N.transfer" "Root.15N.transfer" "Plant.15N.transfer"
## [16] "Root.qPCR.nLSU" "Root.qPCR.Mt5" "Tsoil.qPCR.nLSU"
## [19] "Tsoil.qPCR.Mt5" "Bsoil.qPCR.nLSU" "Bsoil.qPCR.Mt5"
## [22] "Nsoil.qPCR.nLSU" "Nsoil.qPCR.Mt5" "Nsoil.V4.qPCR"
## [25] "Nsoil.CTO.qPCR" "Nsoil.Eub.qPCR" "Nsoil.H.qPCR"
## [28] "Tsoil.V4.qPCR" "Tsoil.CTO.qPCR" "Tsoil.Eub.qPCR"
## [31] "Tsoil.H.qPCR" "colonization.H" "colonization.A"
## [34] "colonization.V"

str(P60.data)

## 'data.frame': 32 obs. of 34 variables:
## $ No : int 1 2 3 4 5 6 7 8 9 10 ...
## $ Nutrient : chr "none" "none" "none" "none" ...
## $ Inoculum : chr "NM" "NM" "NM" "NM" ...
## $ Shoot.DW : num 1.91 2.54 2.65 2.66 2.6 ...
## $ Root.DW : num 1.21 2.84 3.07 1.96 3.23 ...
## $ Total.DW : num 3.12 5.38 5.72 4.62 5.83 ...
## $ Shoot.P : num 0.544 0.511 0.435 0.441 0.445 ...
## $ Root.P : num 0.518 0.465 0.633 0.527 0.521 ...
## $ Plant.total.P : num 1.67 2.62 3.09 2.21 2.84 ...
## $ Shoot.N : num 980 765 1030 1294 836 ...
## $ Root.N : num 425 617 945 607 522 ...
## $ Plant.total.N : num 1405 1382 1975 1900 1358 ...
## $ Shoot.15N.transfer: num -0.01209 0.01396 -0.00427 -0.00228 33.04913 ...
## $ Root.15N.transfer : num 0.00522 0.02145 -0.02296 -0.01382 11.93898 ...
## $ Plant.15N.transfer: num -0.00686 0.03541 -0.02723 -0.0161 44.98811 ...
## $ Root.qPCR.nLSU : num 0 0 0 0 0 0 0 0 0 0 ...
## $ Root.qPCR.Mt5 : num 0 0 0 0 0 0 0 0 0 0 ...
## $ Tsoil.qPCR.nLSU : num 0 192.6 0 744.6 17.4 ...
## $ Tsoil.qPCR.Mt5 : num 394.7 286.5 495.8 966 20.4 ...
## $ Bsoil.qPCR.nLSU : num 0 0 0 0 0 0 0 0 0 0 ...
## $ Bsoil.qPCR.Mt5 : num 0 8.43 0 0 0 ...
## $ Nsoil.qPCR.nLSU : num 0 34.3 56.5 0 0 ...
## $ Nsoil.qPCR.Mt5 : num 57 96.7 112.1 63.1 32.6 ...
## $ Nsoil.V4.qPCR : num 4563 3216 2291 5188 43372 ...
## $ Nsoil.CTO.qPCR : num 2.41 10.26 1.92 3.83 53.51 ...
## $ Nsoil.Eub.qPCR : num 55025 39696 32473 27037 191023 ...
## $ Nsoil.H.qPCR : num 3592 1528 1241 686 17959 ...
## $ Tsoil.V4.qPCR : num 70583 5139 2566 35164 3016 ...
## $ Tsoil.CTO.qPCR : num 130.83 45.83 98.46 48.41 5.95 ...
## $ Tsoil.Eub.qPCR : num 137828 104078 106260 51199 19152 ...
## $ Tsoil.H.qPCR : num 2641 9512 4044 2435 350 ...
## $ colonization.H : int 12 4 8 2 2 0 8 0 8 10 ...
## $ colonization.A : int 0 0 0 0 0 0 0 0 0 0 ...
## $ colonization.V : int 0 0 0 0 0 0 0 0 0 0 ...

view(P60.data)
attach(P60.data)

P60.data$Inoculum <- factor(P60.data$Inoculum , levels=c("NM", "M.LPA9"))
P60.data$Nutrient <- factor(P60.data$Nutrient , levels=c("none", "chitin","phytate.mineral.N","mineral.NP"))


*#*Shoot.DW

*#same codes were used for other factors.*
P60.data %>%
 group_by(Inoculum, Nutrient) %>%
 get_summary_stats(Shoot.DW, type = "mean_sd")

## # A tibble: 8 x 6
## Nutrient Inoculum variable n mean sd
## <fct> <fct> <chr> <dbl> <dbl> <dbl>
## 1 none NM Shoot.DW 4 2.44 0.356
## 2 chitin NM Shoot.DW 4 2.47 0.464
## 3 phytate.mineral.N NM Shoot.DW 4 2.56 0.048
## 4 mineral.NP NM Shoot.DW 4 2.59 0.116
## 5 none M.LPA9 Shoot.DW 4 2.53 0.212
## 6 chitin M.LPA9 Shoot.DW 4 2.27 0.227
## 7 phytate.mineral.N M.LPA9 Shoot.DW 4 2.49 0.265
## 8 mineral.NP M.LPA9 Shoot.DW 4 2.63 0.307

*#Check ANOVA assumptions*
*#Identify outliers by groups:*

P60.data %>%
 group_by(Inoculum, Nutrient) %>%
 identify_outliers(Shoot.DW)

## # A tibble: 1 x 36
## Nutrient Inoculum No Shoot.DW Root.DW Total.DW Shoot.P Root.P Plant.total.P
## <fct> <fct> <int> <dbl> <dbl> <dbl> <dbl> <dbl> <dbl>
## 1 none NM 1 1.91 1.21 3.12 0.544 0.518 1.67
## # ... with 27 more variables: Shoot.N <dbl>, Root.N <dbl>, Plant.total.N <dbl>,
## # Shoot.15N.transfer <dbl>, Root.15N.transfer <dbl>,
## # Plant.15N.transfer <dbl>, Root.qPCR.nLSU <dbl>, Root.qPCR.Mt5 <dbl>,
## # Tsoil.qPCR.nLSU <dbl>, Tsoil.qPCR.Mt5 <dbl>, Bsoil.qPCR.nLSU <dbl>,
## # Bsoil.qPCR.Mt5 <dbl>, Nsoil.qPCR.nLSU <dbl>, Nsoil.qPCR.Mt5 <dbl>,
## # Nsoil.V4.qPCR <dbl>, Nsoil.CTO.qPCR <dbl>, Nsoil.Eub.qPCR <dbl>,
## # Nsoil.H.qPCR <dbl>, Tsoil.V4.qPCR <dbl>, Tsoil.CTO.qPCR <dbl>, ...

*#Normality assumption*
*#by analyzing the model residuals. QQ plot and Shapiro-Wilk test of normality are used.*

*# Build the linear model*

model <- lm(Shoot.DW ~ Inoculum*Nutrient,
 data = P60.data)

*# Create a QQ plot of residuals*
ggqqplot(residuals(model))


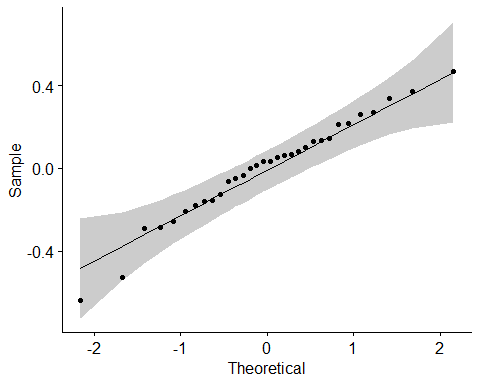


*# Compute Shapiro-Wilk test of normality*
shapiro_test(residuals(model))

## # A tibble: 1 x 3
## variable statistic p.value
## <chr> <dbl> <dbl>
## 1 residuals(model) 0.975 0.641

*#Check normality assumption by groups*
P60.data %>%
 group_by(Inoculum, Nutrient) %>%
 shapiro_test(Shoot.DW)

## # A tibble: 8 x 5
## Nutrient Inoculum variable statistic p
## <fct> <fct> <chr> <dbl> <dbl>
## 1 none NM Shoot.DW 0.742 0.0326
## 2 chitin NM Shoot.DW 0.936 0.628
## 3 phytate.mineral.N NM Shoot.DW 0.962 0.791
## 4 mineral.NP NM Shoot.DW 0.987 0.944
## 5 none M.LPA9 Shoot.DW 0.892 0.392
## 6 chitin M.LPA9 Shoot.DW 0.992 0.969
## 7 phytate.mineral.N M.LPA9 Shoot.DW 0.950 0.715
## 8 mineral.NP M.LPA9 Shoot.DW 0.928 0.581

*#Create QQ plots for each cell of design*
ggqqplot(P60.data, "Shoot.DW", ggtheme = theme_bw()) +
 facet_grid(Inoculum ~ Nutrient)


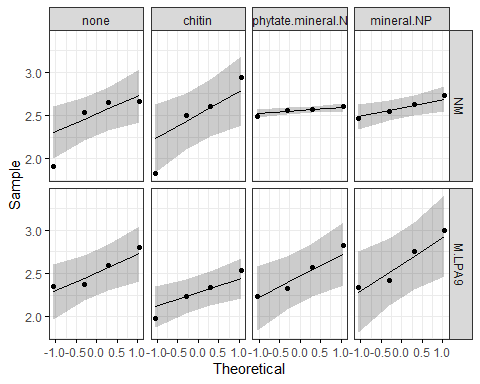


*#Homogneity of variance assumption*
P60.data%>% levene_test(Shoot.DW ~ Inoculum*Nutrient)

## # A tibble: 1 x 4
## df1 df2 statistic p
## <int> <int> <dbl> <dbl>
## 1 7 24 0.924 0.506

*#Computation*
res.aov <- P60.data %>% anova_test(Shoot.DW ~ Inoculum*Nutrient)

## Coefficient covariances computed by hccm()

res.aov

## ANOVA Table (type II tests)
##
## Effect DFn DFd F p p<.05 ges
## 1 Inoculum 1 24 0.131 0.721 0.005
## 2 Nutrient 3 24 1.020 0.401 0.113
## 3 Inoculum:Nutrient 3 24 0.415 0.744 0.049

*#Post-hoc tests*
*#Compute pairwise comparisons*

*#Compare the different treatments by Sp and Inoculum variables:*
pwc1 <- P60.data %>%
 group_by(Nutrient) %>%
 emmeans_test(Shoot.DW ~Inoculum , p.adjust.method = "bonferroni")
pwc1

## # A tibble: 4 x 10
## Nutrient term .y. group1 group2 df statistic p p.adj p.adj.signif
## * <chr> <chr> <chr> <chr> <chr> <dbl> <dbl> <dbl> <dbl> <chr>
## 1 chitin Inocu~ Shoo~ NM M.LPA9 24 1.01 0.325 0.325 ns
## 2 mineral.NP Inocu~ Shoo~ NM M.LPA9 24 -0.178 0.860 0.860 ns
## 3 none Inocu~ Shoo~ NM M.LPA9 24 -0.456 0.652 0.652 ns
## 4 phytate.m~ Inocu~ Shoo~ NM M.LPA9 24 0.352 0.728 0.728 ns

pwc2 <- P60.data %>%
 group_by(Inoculum) %>%
 emmeans_test(Shoot.DW ~Nutrient , p.adjust.method = "bonferroni")
pwc2

## # A tibble: 12 x 10
## Inoculum term .y. group1 group2 df statistic p p.adj p.adj.signif
## * <chr> <chr> <chr> <chr> <chr> <dbl> <dbl> <dbl> <dbl> <chr>
## 1 M.LPA9 Nutri~ Shoo~ none chitin 24 1.31 0.202 1 ns
## 2 M.LPA9 Nutri~ Shoo~ none phyta~ 24 0.215 0.832 1 ns
## 3 M.LPA9 Nutri~ Shoo~ none miner~ 24 -0.497 0.624 1 ns
## 4 M.LPA9 Nutri~ Shoo~ chitin phyta~ 24 -1.10 0.283 1 ns
## 5 M.LPA9 Nutri~ Shoo~ chitin miner~ 24 -1.81 0.0828 0.497 ns
## 6 M.LPA9 Nutri~ Shoo~ phyta~ miner~ 24 -0.712 0.483 1 ns
## 7 NM Nutri~ Shoo~ none chitin 24 -0.149 0.883 1 ns
## 8 NM Nutri~ Shoo~ none phyta~ 24 -0.594 0.558 1 ns
## 9 NM Nutri~ Shoo~ none miner~ 24 -0.775 0.446 1 ns
## 10 NM Nutri~ Shoo~ chitin phyta~ 24 -0.445 0.660 1 ns
## 11 NM Nutri~ Shoo~ chitin miner~ 24 -0.627 0.537 1 ns
## 12 NM Nutri~ Shoo~ phyta~ miner~ 24 -0.182 0.857 1 ns

*#bargraph26*

P60.data2 <- summarySE(P60.data, measurevar="Shoot.DW",
 groupvars= c("Inoculum", "Nutrient"), na.rm = TRUE)


tiff("Shoot.DW.tiff", width = 6, height = 5, units = 'in', res = 300)


bar<- ggplot(P60.data2, aes(x=Nutrient, Shoot.DW, fill=Inoculum)) +
 scale_fill_manual(values=c("M.LPA9" = "aquamarine4", "NM" = "antiquewhite1"))+
 geom_bar(stat="identity", position=position_dodge(width=0.8), colour="darkslategray",width=0.8) +
 labs( y="Shoot dry biomass (g)", x="Nutrient treatments")+
 theme(axis.title = element_text(size=16, face = "bold"),
 legend.text=element_text(size=rel(1)),
 axis.text.x = element_text(color = "black", size = 12),
 axis.text.y = element_text(color = "black", size = 12)) +
 theme_bw()


bar

bar + geom_errorbar(data=P60.data2, aes(ymin= Shoot.DW-se, ymax= Shoot.DW+se),
 size=0.5, colour="darkslategray",
 width=.20, stat = "identity",
 position = position_dodge(width = 0.9), group = P60.data2$Nutrient)


dev.off()

## png
## 2

library("ggplot2")
library("microeco")
library("devtools")

library("usethis")
library("SpiecEasi")
library("ggpubr")
library("rhdf5")
library("file2meco")
library("RJSONIO")
library("WGCNA")

library("dynamicTreeCut")
library("fastcluster")
library("MASS")

library("GUniFrac")

library("randomForest")

library("ggdendro")
library("ggrepel")
library("agricolae")

library("gridExtra")

library("picante")

library("lattice")
library("nlme")
library("pheatmap")
library("tidytree")

library("igraph")

library("readr")
library("rgexf")
library("usethis")
library("dplyr")

library("magrittr")
library("writexl")
library("ggraph")
library("networkD3")
library("chorddiag")
library("htmlwidgets")

library("webshot")
library("NetCoMi")

library("devtools")
library("BiocManager")

setwd ("A:\\ ")

# #Network Analysis

# Hyphal compartment _ control (The same codes were used for constructing the networks of other treatments

otu_table<-read.table("otu_Nsoil_M_none.txt", sep = '\t' )

taxonomy_table<-read.table("taxonomy_table.txt", sep = '\t' )

sample_info<-read.table("sample_info_Nsoil_M_none.txt", header = T, sep = '\t' )

View(otu_table)
View(taxonomy_table)
View(sample_info)


rownames(otu_table) <- paste0("OTU", 1:nrow(otu_table))
colnames(otu_table)<- sample_info$SampleID
View(otu_table)


rownames(taxonomy_table) <- rownames(otu_table)
colnames(taxonomy_table) <- c("Kingdom", "Phylum", "Class", "Order", "Family", "Genus", "Species")
View(taxonomy_table)


rownames(sample_info)<- colnames(otu_table)
View(sample_info)


#Make sure that the data types are all data.frame

class(otu_table)

## [1] "data.frame"

class(taxonomy_table)

## [1] "data.frame"

class(sample_info)

## [1] "data.frame"

#Generally, users' taxonomic table may have some chaotic information,
#such as NA, unidentified and unknown. These information can potentially
#influence the following taxonomic abundance calculation and other
#taxonomy-based analysis. So it is usually necessary to clean this data using
#the tidy_taxonomy function. Another very important result of this operation is
#to unify the taxonomic prefix automatically, e.g. transforming D_1__ to p__
#for phylum level or adding p__ to phylum directly.

# make the taxonomic information unified, very important

#taxonomy_table %<>% tidy_taxonomy


# Let's create a microtable object with more information
dataset<- microtable$new(sample_table = sample_info, otu_table = otu_table, tax_table = taxonomy_table)

## 2044 taxa are removed from the otu_table, as the abundance is 0 ...

dataset

## microtable-class object:
## sample_table have 4 rows and 5 columns
## otu_table have 3091 rows and 4 columns
## tax_table have 5135 rows and 7 columns

class(dataset)

## [1] "microtable" "R6"

#Then let's use sample_sums() to check the sequence numbers in each sample.

dataset$sample_sums() %>% range

## [1] 5099969 5100014

#Let's calculate the taxa abundance at each taxonomic rank

dataset$cal_abund()

## The row number of tax_table is not equal to that of otu_table ...

## Automatically applying tidy_dataset() function to trim the data ...

## microtable-class object:
## sample_table have 4 rows and 5 columns
## otu_table have 3091 rows and 4 columns
## tax_table have 3091 rows and 7 columns

## The result is stored in object$taxa_abund ...

class(dataset$taxa_abund)

## [1] "list"

#The function save_abund() can be used to save the taxa abundance file to a local place easily.

dataset$save_abund(dirpath = "taxa_abund_Nsoil_M_none")

#Network is a frequently used approach to study the co-occurrence patterns
#in microbial ecology (Deng et al. 2012; Faust and Raes 2012; Coyte, Schluter, and Foster 2015).

#The network construction approaches can be classified into two types:
#correlation-based and non correlation-based.

# SparCC method, require SpiecEasi package,
#see https://chiliubio.github.io/microeco_tutorial/intro.html#spieceasi for the installation
# SparCC is very slow, so consider filtering more species with low abundance

# SparCC method, from NetCoMi package; https://github.com/stefpeschel/NetCoMi
#t1 <- trans_network$new(dataset = dataset, cor_method = "sparcc",taxa_level ="Genus", use_sparcc_method = "NetCoMi", filter_thres = 0.001)


# SparCC method, from SpiecEasi package, see https://github.com/zdk123/SpiecEasi for the installation
t1 <- trans_network$new(dataset = dataset, cor_method = "sparcc", taxa_level ="Genus", use_sparcc_method = "SpiecEasi", filter_thres = 0.001)

## After filtering, 95 features are remained ...

## The correlation result list is stored in object$res_cor_p ...

#The parameter COR_cut can be used to select the correlation threshold.
#Furthermore, COR_optimization = TRUE represent using RMT theory to find the
#optimized correlation threshold instead of the COR_cut(Deng et al. 2012).

# construct network; require igraph package
t1$cal_network(p_thres = 0.01, COR_optimization = TRUE, add_taxa_name = "Phylum",usename_rawtaxa_when_taxalevel_notOTU= TRUE)

## ---------------- 2023-07-14 10:24:37 : Start ----------------

## Perform p value adjustment with fdr method ...

## Start COR optimizing ...

## [1] 0.05
## [1] 0.1
## [1] 0.2
## [1] 0.25
## [1] 0.3
## [1] 0.35
## [1] 0.4
## [1] 0.45
## [1] 0.5
## [1] 0.6
## [1] 0.65
## [1] 0.7
## [1] 0.75
## [1] 0.8

## The optimized COR threshold: 0.7...

## ---------------- 2023-07-14 10:24:38 : Finish ----------------

## The result network is stored in object$res_network ...

# return t1$res_network


# add modules in the network
t1$cal_module()

## Totally, 13 modules are idenfified ...

## Modules are assigned in network with attribute name -- module ...

# calculate network attributes
t1$cal_network_attr()

## Result is stored in object$res_network_attr ...

t1$res_network_attr

##
## Vertex 69.00000000
## Edge 67.00000000
## Average_degree 1.94202899
## Average_path_length 5.10123436
## Network_diameter 12.00000000
## Clustering_coefficient 0.02500000
## Density 0.02855925
## Heterogeneity 0.66603970
## Centralization 0.05967604

# get node properties
t1$get_node_table(node_roles = TRUE)

## The nodes (6) with NaN in z will be filtered ...

## Result is stored in object$res_node_table ...

t1$res_node_table

## name degree betweenness Abundance module z p
## OTU4339 OTU4339 4 438 17.2949191 M1 1.6583124 0.3750000
## OTU1 OTU1 2 196 11.4537804 M1 0.0000000 0.0000000
## OTU5 OTU5 1 0 6.9419203 M4 -0.7144958 0.0000000
## OTU3597 OTU3597 1 0 6.2808168 M10 -0.5773503 0.0000000
## OTU4338 OTU4338 1 0 5.0527172 M3 -0.6822210 0.0000000
## OTU32 OTU32 6 230 1.8160512 M4 2.0640989 0.0000000
## OTU13 OTU13 3 53 1.6667580 M7 1.4142136 0.0000000
## OTU8 OTU8 2 102 1.6464802 M5 0.0000000 0.5000000
## OTU29 OTU29 2 52 1.6316656 M1 0.0000000 0.0000000
## OTU15 OTU15 1 0 1.5760837 M2 -0.8400269 0.3750000
## OTU2 OTU2 1 0 1.5540445 M13 NA NA
## OTU47 OTU47 3 152 1.2979560 M1 0.8291562 0.0000000
## OTU21 OTU21 4 251 1.2829195 M2 1.1760376 0.0000000
## OTU4340 OTU4340 1 0 1.2378986 M9 -0.5773503 0.0000000
## OTU22 OTU22 3 86 1.1155438 M4 0.3969421 0.4444444
## OTU34 OTU34 1 0 1.0104485 M5 -1.2247449 0.0000000
## OTU78 OTU78 2 52 0.9659660 M6 -0.1587768 0.5000000
## OTU3600 OTU3600 3 371 0.8522266 M1 0.8291562 0.0000000
## OTU50 OTU50 1 0 0.8418544 M1 -0.8291562 0.6111111
## OTU3 OTU3 2 78 0.7373475 M7 0.0000000 0.0000000
## OTU173 OTU173 6 811 0.7137094 M3 2.0466631 0.0000000
## OTU9 OTU9 2 1 0.5810647 M10 1.1547005 0.5000000
## OTU12 OTU12 1 0 0.5735499 M12 NA NA
## OTU4348 OTU4348 1 0 0.5717899 M12 NA NA
## OTU102 OTU102 1 0 0.5491386 M1 -0.8291562 0.0000000
## OTU85 OTU85 2 22 0.4468300 M4 -0.1587768 0.0000000
## OTU53 OTU53 1 0 0.4035583 M13 NA NA
## OTU55 OTU55 1 0 0.3990074 M4 -0.7144958 0.0000000
## OTU3631 OTU3631 1 0 0.3956182 M6 -0.7144958 0.4444444
## OTU238 OTU238 4 436 0.3680488 M1 1.6583124 0.0000000
## OTU36 OTU36 1 0 0.3680107 M11 NA NA
## OTU354 OTU354 1 0 0.3403074 M2 -0.8400269 0.5000000
## OTU272 OTU272 3 192 0.3354406 M5 1.2247449 0.0000000
## OTU172 OTU172 2 1 0.3224738 M9 1.1547005 0.0000000
## OTU30 OTU30 2 125 0.3124068 M7 0.0000000 0.0000000
## OTU7 OTU7 6 406 0.3044881 M6 2.0640989 0.0000000
## OTU57 OTU57 2 1 0.2938982 M8 1.1547005 0.0000000
## OTU293 OTU293 1 0 0.2803056 M8 -0.5773503 0.5000000
## OTU11 OTU11 1 0 0.2786801 M1 -0.8291562 0.0000000
## OTU490 OTU490 4 585 0.2769794 M2 1.1760376 0.0000000
## OTU150 OTU150 2 52 0.2765788 M3 -0.1364442 0.0000000
## OTU124 OTU124 1 0 0.2673594 M1 -0.8291562 0.0000000
## OTU81 OTU81 4 170 0.2587572 M2 1.1760376 0.0000000
## OTU3604 OTU3604 1 0 0.2565837 M1 -0.8291562 0.0000000
## OTU116 OTU116 2 420 0.2494303 M3 -0.1364442 0.0000000
## OTU4463 OTU4463 1 0 0.2468091 M5 -1.2247449 0.0000000
## OTU3595 OTU3595 1 0 0.2457993 M9 -0.5773503 0.0000000
## OTU286 OTU286 1 0 0.2427501 M10 -0.5773503 0.0000000
## OTU3606 OTU3606 1 0 0.2223483 M3 -0.6822210 0.0000000
## OTU152 OTU152 4 372 0.1957469 M3 0.9551094 0.0000000
## OTU303 OTU303 2 0 0.1887634 M6 -0.1587768 0.0000000
## OTU3616 OTU3616 1 0 0.1860689 M7 -1.4142136 0.0000000
## OTU472 OTU472 1 0 0.1789958 M3 -0.6822210 0.0000000
## OTU206 OTU206 1 0 0.1756388 M3 -0.6822210 0.3750000
## OTU710 OTU710 1 0 0.1587495 M2 -0.8400269 0.0000000
## OTU130 OTU130 2 192 0.1582763 M5 0.0000000 0.7500000
## OTU67 OTU67 3 272 0.1525747 M5 1.2247449 0.0000000
## OTU268 OTU268 1 0 0.1486701 M1 -0.8291562 0.0000000
## OTU189 OTU189 1 0 0.1429874 M6 -0.7144958 0.0000000
## OTU193 OTU193 2 52 0.1401739 M5 0.0000000 0.0000000
## OTU615 OTU615 1 0 0.1375357 M11 NA NA
## OTU181 OTU181 3 102 0.1286448 M6 0.3969421 0.0000000
## OTU401 OTU401 2 52 0.1277248 M2 -0.1680054 0.0000000
## OTU271 OTU271 1 0 0.1247906 M4 -0.7144958 0.0000000
## OTU27 OTU27 1 0 0.1239490 M2 -0.8400269 0.3750000
## OTU406 OTU406 1 0 0.1222888 M6 -0.7144958 0.0000000
## OTU52 OTU52 1 0 0.1169774 M8 -0.5773503 0.0000000
## OTU4381 OTU4381 2 22 0.1166766 M7 0.0000000 0.5000000
## OTU554 OTU554 2 52 0.1103496 M4 -0.1587768 0.0000000
## taxa_roles Kingdom Phylum Class
## OTU4339 Peripheral nodes Fungi Ascomycota Eurotiomycetes
## OTU1 Peripheral nodes Bacteria Actinobacteria Actinobacteria
## OTU5 Peripheral nodes Bacteria un_Bacteria un_Bacteria
## OTU3597 Peripheral nodes Protists Amoebozoa Amoebozoa_XX
## OTU4338 Peripheral nodes Fungi Ascomycota Sordariomycetes
## OTU32 Peripheral nodes Bacteria Proteobacteria Betaproteobacteria
## OTU13 Peripheral nodes Bacteria Proteobacteria Gammaproteobacteria
## OTU8 Peripheral nodes Bacteria Proteobacteria Alphaproteobacteria
## OTU29 Peripheral nodes Bacteria Verrucomicrobia Verrucomicrobia_Subdiv3
## OTU15 Peripheral nodes Bacteria Actinobacteria Actinobacteria
## OTU2 <NA> Bacteria Proteobacteria Deltaproteobacteria
## OTU47 Peripheral nodes Bacteria Proteobacteria Alphaproteobacteria
## OTU21 Peripheral nodes Bacteria Actinobacteria Thermoleophilia
## OTU4340 Peripheral nodes Fungi Ascomycota Dothideomycetes
## OTU22 Peripheral nodes Bacteria Actinobacteria Actinobacteria
## OTU34 Peripheral nodes Bacteria Acidobacteria Acidobacteria_Gp3
## OTU78 Peripheral nodes Bacteria Proteobacteria Alphaproteobacteria
## OTU3600 Peripheral nodes Protists Amoebozoa Variosea
## OTU50 Peripheral nodes Bacteria Proteobacteria Alphaproteobacteria
## OTU3 Peripheral nodes Bacteria Proteobacteria Alphaproteobacteria
## OTU173 Peripheral nodes Bacteria Proteobacteria Betaproteobacteria
## OTU9 Peripheral nodes Bacteria Actinobacteria Actinobacteria
## OTU12 <NA> Bacteria Firmicutes Bacilli
## OTU4348 <NA> Fungi Ascomycota Eurotiomycetes
## OTU102 Peripheral nodes Bacteria Bacteroidetes Cytophagia
## OTU85 Peripheral nodes Bacteria Proteobacteria Betaproteobacteria
## OTU53 <NA> Bacteria Proteobacteria Alphaproteobacteria
## OTU55 Peripheral nodes Bacteria Planctomycetes Planctomycetacia
## OTU3631 Peripheral nodes Protists Rhizaria Sarcomonadea
## OTU238 Peripheral nodes Bacteria Actinobacteria Thermoleophilia
## OTU36 <NA> Bacteria Acidobacteria Acidobacteria_Gp16
## OTU354 Peripheral nodes Bacteria Actinobacteria Actinobacteria
## OTU272 Peripheral nodes Bacteria Proteobacteria Betaproteobacteria
## OTU172 Peripheral nodes Bacteria Firmicutes Bacilli
## OTU30 Peripheral nodes Bacteria Proteobacteria Deltaproteobacteria
## OTU7 Peripheral nodes Bacteria Firmicutes Bacilli
## OTU57 Peripheral nodes Bacteria Actinobacteria Actinobacteria
## OTU293 Peripheral nodes Bacteria Actinobacteria Actinobacteria
## OTU11 Peripheral nodes Bacteria Proteobacteria Alphaproteobacteria
## OTU490 Peripheral nodes Bacteria Acidobacteria Acidobacteria_Gp6
## OTU150 Peripheral nodes Bacteria Proteobacteria Alphaproteobacteria
## OTU124 Peripheral nodes Bacteria Actinobacteria Acidimicrobiia
## OTU81 Peripheral nodes Bacteria Actinobacteria Actinobacteria
## OTU3604 Peripheral nodes Protists Excavata Jakobida
## OTU116 Peripheral nodes Bacteria Proteobacteria Deltaproteobacteria
## OTU4463 Peripheral nodes Fungi un_Fungi un_Fungi
## OTU3595 Peripheral nodes Protists Alveolata Colpodea
## OTU286 Peripheral nodes Bacteria Actinobacteria Thermoleophilia
## OTU3606 Peripheral nodes Protists Rhizaria Rhizaria_XX
## OTU152 Peripheral nodes Bacteria Proteobacteria Oligoflexia
## OTU303 Peripheral nodes Bacteria Actinobacteria un_Actinobacteria
## OTU3616 Peripheral nodes Protists Amoebozoa Variosea
## OTU472 Peripheral nodes Bacteria Proteobacteria Deltaproteobacteria
## OTU206 Peripheral nodes Bacteria Proteobacteria Alphaproteobacteria
## OTU710 Peripheral nodes Bacteria Proteobacteria Deltaproteobacteria
## OTU130 Connectors Bacteria Actinobacteria Acidimicrobiia
## OTU67 Peripheral nodes Bacteria Bacteroidetes Sphingobacteriia
## OTU268 Peripheral nodes Bacteria Proteobacteria Alphaproteobacteria
## OTU189 Peripheral nodes Bacteria Proteobacteria Alphaproteobacteria
## OTU193 Peripheral nodes Bacteria Proteobacteria Alphaproteobacteria
## OTU615 <NA> Bacteria Proteobacteria Gammaproteobacteria
## OTU181 Peripheral nodes Bacteria Actinobacteria Thermoleophilia
## OTU401 Peripheral nodes Bacteria Gemmatimonadetes Gemmatimonadetes
## OTU271 Peripheral nodes Bacteria Proteobacteria Alphaproteobacteria
## OTU27 Peripheral nodes Bacteria Chloroflexi un_Chloroflexi
## OTU406 Peripheral nodes Bacteria Proteobacteria Alphaproteobacteria
## OTU52 Peripheral nodes Bacteria Firmicutes Bacilli
## OTU4381 Peripheral nodes Fungi Ascomycota Dothideomycetes
## OTU554 Peripheral nodes Bacteria Proteobacteria Oligoflexia
## Order Family
## OTU4339 Eurotiales Trichocomaceae
## OTU1 Propionibacteriales Kribbellaceae
## OTU5 un_Bacteria un_Bacteria
## OTU3597 Amoebozoa_XXX Amoebozoa_XXXX
## OTU4338 Hypocreales Nectriaceae
## OTU32 Burkholderiales Comamonadaceae
## OTU13 Legionellales Legionellaceae
## OTU8 Caulobacterales Caulobacteraceae
## OTU29 un_Verrucomicrobia_Subdiv3 un_Verrucomicrobia_Subdiv3
## OTU15 Propionibacteriales Nocardioidaceae
## OTU2 Myxococcales Cystobacteraceae
## OTU47 Rhizobiales Methylobacteriaceae
## OTU21 Solirubrobacterales un_Solirubrobacterales
## OTU4340 Pleosporales Pleosporales_Incertae sedis
## OTU22 Streptomycetales Streptomycetaceae
## OTU34 un_Acidobacteria_Gp3 un_Acidobacteria_Gp3
## OTU78 Rhizobiales un_Rhizobiales
## OTU3600 ATCC50593-Flamella-WIM80-lineage Flamella-lineage
## OTU50 Sphingomonadales Sphingosinicellaceae
## OTU3 Sphingomonadales Sphingomonadaceae
## OTU173 Burkholderiales Oxalobacteraceae
## OTU9 Micrococcales Promicromonosporaceae
## OTU12 Bacillales un_Bacillales
## OTU4348 Eurotiales Trichocomaceae
## OTU102 Cytophagales Fulvivirgaceae
## OTU85 Burkholderiales Comamonadaceae
## OTU53 un_Alphaproteobacteria un_Alphaproteobacteria
## OTU55 Pirellulales un_Pirellulales
## OTU3631 Glissomonadida Allapsidae
## OTU238 un_Thermoleophilia un_Thermoleophilia
## OTU36 un_Acidobacteria_Gp16 un_Acidobacteria_Gp16
## OTU354 Pseudonocardiales Pseudonocardiaceae
## OTU272 un_Betaproteobacteria un_Betaproteobacteria
## OTU172 Bacillales Paenibacillaceae
## OTU30 Myxococcales Polyangiaceae
## OTU7 Bacillales Bacillaceae
## OTU57 Mycobacteriales Nocardiaceae
## OTU293 Micromonosporales Micromonosporaceae
## OTU11 Rhizobiales Rhizobiaceae
## OTU490 un_Acidobacteria_Gp6 un_Acidobacteria_Gp6
## OTU150 Sphingomonadales Sphingomonadaceae
## OTU124 Acidimicrobiales un_Acidimicrobiales
## OTU81 Streptomycetales Streptomycetaceae
## OTU3604 Jakobida_X Jakobidae
## OTU116 un_Deltaproteobacteria un_Deltaproteobacteria
## OTU4463 un_Fungi un_Fungi
## OTU3595 Colpodea_X Colpodida
## OTU286 Gaiellales Gaiellaceae
## OTU3606 Rhizaria_XXX Rhizaria_XXXX
## OTU152 Oligoflexales Oligoflexaceae
## OTU303 un_Actinobacteria un_Actinobacteria
## OTU3616 Variosea_X Filamoebidae
## OTU472 Myxococcales Cystobacteraceae
## OTU206 Caulobacterales Caulobacteraceae
## OTU710 Myxococcales un_Myxococcales
## OTU130 Acidimicrobiales Iamiaceae
## OTU67 Sphingobacteriales Sphingobacteriaceae
## OTU268 Rhizobiales Devosiaceae
## OTU189 Rhizobiales Hyphomicrobiaceae
## OTU193 Rhodospirillales Rhodospirillaceae
## OTU615 un_Gammaproteobacteria un_Gammaproteobacteria
## OTU181 Solirubrobacterales Baekduiaceae
## OTU401 Gemmatimonadales Gemmatimonadaceae
## OTU271 Sphingomonadales Erythrobacteraceae
## OTU27 un_Chloroflexi un_Chloroflexi
## OTU406 Rhizobiales Bradyrhizobiaceae
## OTU52 Bacillales Bacillaceae
## OTU4381 Pleosporales Pleosporaceae
## OTU554 Bacteriovoracales Bacteriovoracaceae
## Genus
## OTU4339 Aspergillus
## OTU1 Kribbella
## OTU5 un_Bacteria
## OTU3597 Amoebozoa_XXXXX
## OTU4338 Haematonectria
## OTU32 un_Comamonadaceae
## OTU13 Legionella
## OTU8 Phenylobacterium
## OTU29 un_Verrucomicrobia_Subdiv3
## OTU15 Nocardioides
## OTU2 Archangium
## OTU47 Microvirga
## OTU21 un_Solirubrobacterales
## OTU4340 Phoma
## OTU22 un_Streptomycetaceae
## OTU34 un_Acidobacteria_Gp3
## OTU78 un_Rhizobiales
## OTU3600 Flamella
## OTU50 Sphingoaurantiacus
## OTU3 Sphingomonas
## OTU173 un_Oxalobacteraceae
## OTU9 Promicromonospora
## OTU12 un_Bacillales
## OTU4348 Eupenicillium
## OTU102 Ohtaekwangia
## OTU85 Ramlibacter
## OTU53 un_Alphaproteobacteria
## OTU55 un_Pirellulales
## OTU3631 Allapsidae_X
## OTU238 un_Thermoleophilia
## OTU36 un_Acidobacteria_Gp16
## OTU354 Pseudonocardia
## OTU272 un_Betaproteobacteria
## OTU172 Brevibacillus
## OTU30 un_Polyangiaceae
## OTU7 Mesobacillus
## OTU57 Nocardia
## OTU293 Micromonospora
## OTU11 un_Rhizobiaceae
## OTU490 un_Acidobacteria_Gp6
## OTU150 un_Sphingomonadaceae
## OTU124 un_Acidimicrobiales
## OTU81 Streptomyces
## OTU3604 Andalucia
## OTU116 un_Deltaproteobacteria
## OTU4463 un_Fungi
## OTU3595 Colpoda
## OTU286 Gaiella
## OTU3606 Rhizaria_XXXXX
## OTU152 Oligoflexus
## OTU303 un_Actinobacteria
## OTU3616 Filamoeba
## OTU472 un_Cystobacteraceae
## OTU206 Brevundimonas
## OTU710 un_Myxococcales
## OTU130 un_Iamiaceae
## OTU67 un_Sphingobacteriaceae
## OTU268 Devosia
## OTU189 Hyphomicrobium
## OTU193 Dongia
## OTU615 un_Gammaproteobacteria
## OTU181 Baekduia
## OTU401 un_Gemmatimonadaceae
## OTU271 un_Erythrobacteraceae
## OTU27 un_Chloroflexi
## OTU406 un_Bradyrhizobiaceae
## OTU52 Fictibacillus
## OTU4381 Alternaria
## OTU554 Peredibacter

# get edge properties
t1$get_edge_table()

## Result is stored in object$res_edge_table ...

t1$get_edge_table

## function ()
## {
## private$check_igraph()
## private$check_network()
## network <- self$res_network
## edges <- t(sapply(1:ecount(network), function(x) ends(network,
## x)))
## edge_label <- E(network)$label
## if (!is.null(E(network)$weight)) {
## edge_weight <- E(network)$weight
## }
## else {
## edge_weight <- rep(NA, times = length(edge_label))
## }
## res_edge_table <- data.frame(edges, edge_label, edge_weight)
## colnames(res_edge_table) <- c("node1", "node2", "label",
## "weight")
## self$res_edge_table <- res_edge_table
## message("Result is stored in object$res_edge_table ...")
## }
## <environment: 0x0000021f506e73a8>

# return t1$res_edge_table


t1$get_adjacency_matrix()

## Result is stored in object$res_adjacency_matrix ...

# return t1$res_adjacency_matrix
t1$get_adjacency_matrix

## function (...)
## {
## private$check_igraph()
## private$check_network()
## network <- self$res_network
## self$res_adjacency_matrix <- as_adjacency_matrix(network,
## ...) %>% as.matrix
## message("Result is stored in object$res_adjacency_matrix ...")
## }
## <environment: 0x0000021f506e73a8>

#Now, we show the eigengene analysis of modules.
#The eigengene of a module, i.e. the first principal component of PCA,
#represents the main variance of the abundance in the species of the module.
t1$cal_eigen()

## Result is stored in object$res_eigen and object$res_eigen_expla ...

t1$cal_eigen

## function ()
## {
## private$check_igraph()
## private$check_network()
## use_abund <- self$data_abund
## if (is.null(self$res_node_table)) {
## message("Run get_node_table function to get the node property table ...")
## self$get_node_table()
## }
## node_table <- self$res_node_table
## res_eigen <- list()
## res_eigen_expla <- c()
## for (i in unique(as.character(node_table$module))) {
## tax_names <- rownames(node_table[as.character(node_table$module) ==
## i, ])
## if (length(tax_names) < 3) {
## next
## }
## if (self$taxa_level != "OTU") {
## network <- self$res_network
## replace_table <- data.frame(V(network)$name, V(network)$taxa,
## stringsAsFactors = FALSE) %>% `row.names<-`(.[,
## 1])
## tax_names <- replace_table[tax_names, 2]
## }
## sel_abund <- use_abund[, tax_names]
## pca_model <- rda(sel_abund)
## sel_scores <- scores(pca_model, choices = 1)$sites %>%
## as.data.frame
## colnames(sel_scores)[1] <- i
## res_eigen[[i]] <- sel_scores
## expla <- paste0(round(pca_model$CA$eig/pca_model$CA$tot.chi *
## 100, 1)[1], "%")
## names(expla) <- i
## res_eigen_expla <- c(res_eigen_expla, expla)
## }
## res_eigen <- do.call(cbind, res_eigen)
## self$res_eigen <- res_eigen
## self$res_eigen_expla <- res_eigen_expla
## message("Result is stored in object$res_eigen and object$res_eigen_expla ...")
## }
## <environment: 0x0000021f506e73a8>
