# Supplementary material for: Nutrient-dependent cross-kingdom interactions in the hyphosphere of an arbuscular mycorrhizal fungus
Source: Front Microbiol. 2024 Jan 4;14:1284648. doi: 10.3389/fmicb.2023.1284648 (PMC10794670; doi:10.3389/fmicb.2023.1284648)
Supplement: Supplementary file 1 [file Data_Sheet_1.zip › Supplementary Material 5.DOCX]

| Target | Application | Name | Name: Sequence 5’ → 3’ (Forward, Reverse and TaqMan probe, if applicable) | Authority |
| --- | --- | --- | --- | --- |
| 16S rRNA gene of ammonia oxidizing bacteria | qPCR | CTO | CTOF: equimolar mixture of CTO189f (A), CTO189f (B), CTO189f (C)  GGAGAAAAGCAGGGGATCG (A)  GGAGGAAAGCAGGGGATCG (B)  GGAGGAAAGTAGGGGATCG (C)  CTOR: CTAGCYTTGTAGTTTCAAACGC (CTO654r) | (2) |
| 16S rRNA gene of bacteria | qPCR | Eub | eub 338: ACTCCTACGGGAGGCAGCAG,  eub518: ATTACCGCGGCTGCTGG | (3,4) |
| Internal transcribed spacer 1 region within the rRNA operon of fungi | qPCR | H | ITS0F: ACTTGGTCATTTAGAGGAAGT,  5.8S: CGCTGCGTTCTTCATCG | (5,6) |
| 28S rRNA gene of *Rhizophagus irregularis* | qPCR | Intra | intraF: TTCGGGTAATCAGCCTTTCG,  intraR: TCAGAGATCAGACAGGTAGCC,  intraProbe: FAM-TTAACCAACCACACGGGCAAGTACA- BHQ1 | (7) |
| Mitochondrial large ribosomal subunit of *Rhizophagus irregularis* | qPCR | mt5 | mt5F: TTTTAGCGATAGCGTAACAGC,  mt5R: TACATCTAGGACAGGGTTTCG,  mt5Probe: FAM-AAACTGCCACTCCCTCCATATCCAA-BHQ1 | (8) |
| V4 region of 18 rRNA gene of protists | qPCR, sequencing | V4 | V4F-IL: TCGTCGGCAGCGTCAGATGTGTATAAGAGACAGNNNNNCCAGCASCYGCGGTAATTCC  V4R-ILGTCTCGTGGGCTCGGAGATGTGTATAAGAGACAGNNNNNACTTTCGTTCTTGATYRA | (9) modified |
| Internal DNA standard | qPCR | ISC | ISCF: CGAACCTGGACTGTTATGATG,  ISCR: AATAAACAATCCCCTGTATTTCAC,  ISCProbe: FAM-CACCAGGCACCAACAACGACCATT-BHQ1 | (8) |
| V4 region of the 16S rRNA gene of prokaryotes | sequencing | Prokaryotic 515-806 | 515-IL: TCGTCGGCAGCGTCAGATGTGTATAAGAGACAGNNNNNGTGYCAGCMGCCGCGGTAA,  806-IL: GTCTCGTGGGCTCGGAGATGTGTATAAGAGACAGNNNNNGGACTACNVGGGTWTCTAAT | (10) |
| Illumina i5 | sequencing |  | AATGATACGGCGACCACCGAGATCTACAC**mmmmmmmm**TCGTCGGCAGCGTC | https://support-docs.illumina.com/SHARE/AdapterSeq/Content/SHARE/AdapterSeq/Nextera/DNAIndexesNXT.htm |
| Illumina i7 | sequencing |  | CAAGCAGAAGACGGCATACGAGAT**nnnnnnnn**GTCTCGTGGGCTCGG |  |

Sequences and authorities for the quantitative real-time PCR (qPCR) and end-point PCR primers and hydrolysis (TaqMan) probes used in the research reported here. Nucleotide nomenclature follows the IUPAC code (1)

**References**

1. Johnson AD. 2010. An extended IUPAC nomenclature code for polymorphic nucleic acids. Bioinformatics 26:1386–1389.
2. Kowalchuk GA, Stephen JR, Boer WD, Prosser JI, Embley TM, Woldendorp JW. 1997. Analysis of ammonia-oxidizing bacteria of the beta subdivision of the class Proteobacteria in coastal sand dunes by denaturing gradient gel electrophoresis and sequencing of PCR-amplified 16S ribosomal DNA fragments. Appl Environ Microbiol 63:1489–1497.
3. Lane DJ. 1991. 16S/23S rRNA sequencing, p. 115–147. In Nucleic Acids Techniques in Bacterial Systematics. John Wiley & Sons, Chichester.
4. Muyzer G, de Waal EC, Uitterlinden AG. 1993. Profiling of complex microbial populations by denaturing gradient gel electrophoresis analysis of polymerase chain reaction-amplified genes coding for 16S rRNA. Applied and Environmental Microbiology 59:695–700.
5. Tedersoo L, Jairus T, Horton BM, Abarenkov K, Suvi T, Saar I, Kõljalg U. 2008. Strong host preference of ectomycorrhizal fungi in a Tasmanian wet sclerophyll forest as revealed by DNA barcoding and taxon-specific primers. New Phytologist 180:479–490.
6. Vilgalys R, Hester M. 1990. Rapid genetic identification and mapping of enzymatically amplified ribosomal DNA from several Cryptococcus species. J Bacteriol 172:4238–4246.
7. Thonar C, Erb A, Jansa J. 2012. Real-time PCR to quantify composition of arbuscular mycorrhizal fungal communities—marker design, verification, calibration and field validation. Molecular Ecology Resources 12:219–232.
8. Couillerot O, Ramírez-Trujillo A, Walker V, Felten A von, Jansa J, Maurhofer M, Défago G, Prigent-Combaret C, Comte G, Caballero-Mellado J, Moënne-Loccoz Y. 2013. Comparison of prominent Azospirillum strains in Azospirillum – Pseudomonas – Glomus consortia for promotion of maize growth. Appl Microbiol Biotechnol 97:4639–4649.
9. Stoeck T, Bass D, Nebel M, Christen R, Jones MDM, Breiner H-W, Richards TA. 2010. Multiple marker parallel tag environmental DNA sequencing reveals a highly complex eukaryotic community in marine anoxic water. Mol Ecol 19 Suppl 1:21–31.
10. Caporaso JG, Lauber CL, Walters WA, Berg-Lyons D, Lozupone CA, Turnbaugh PJ, Fierer N, Knight R. 2011. Global patterns of 16S rRNA diversity at a depth of millions of sequences per sample. Proc Natl Acad Sci U S A 108:4516–4522.
