# Supplementary material for: Nutrient-dependent cross-kingdom interactions in the hyphosphere of an arbuscular mycorrhizal fungus
Source: Front Microbiol. 2024 Jan 4;14:1284648. doi: 10.3389/fmicb.2023.1284648 (PMC10794670; doi:10.3389/fmicb.2023.1284648)
Supplement: Supplementary file 1 [file Data_Sheet_1.zip › Supplementary Tables and Figures 1.docx]

Supplementary information

| a) Shoot dry biomass (g) b) Root dry biomass (g) | |
| --- | --- |
| 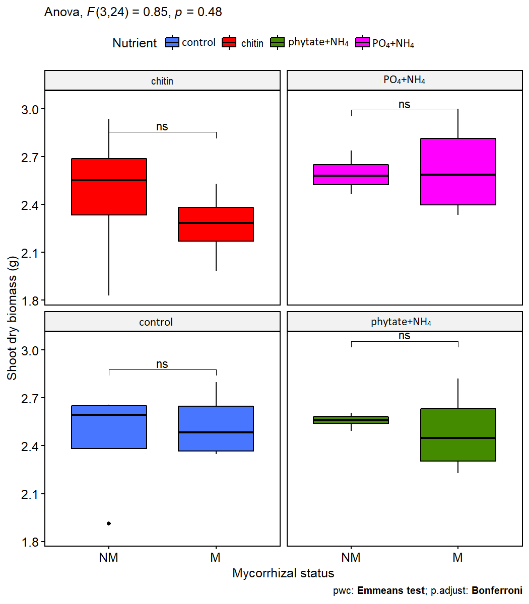 | **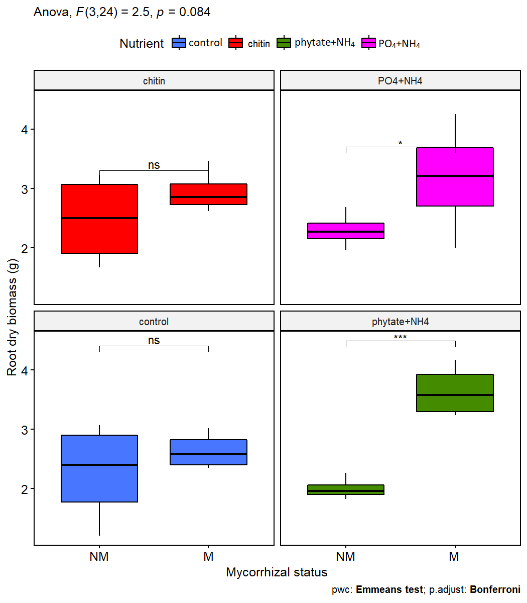** |
| c) Total dry biomass (g) | **d) Shoot P concentration (mg/g)** |
| 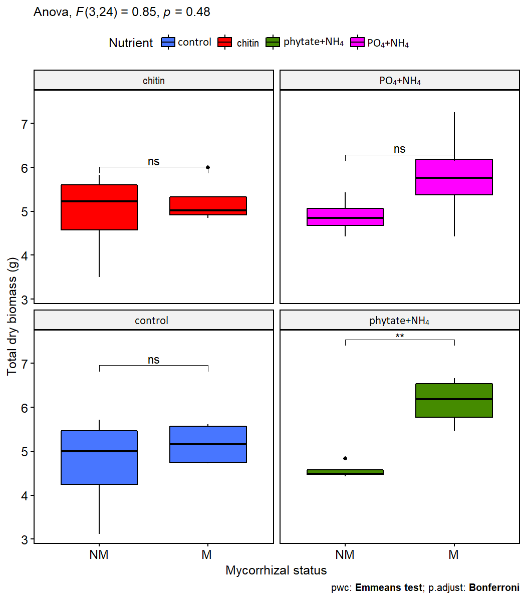 | **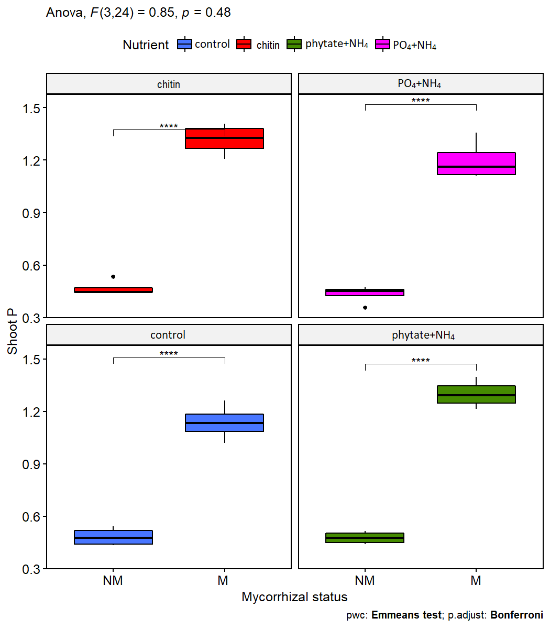** |
| e) Root P concentration (mg/g) | **f) Total P content (mg/pot)** |
| 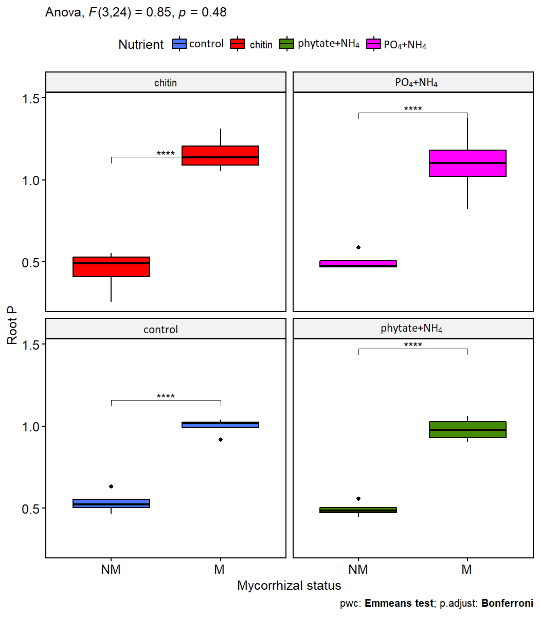 | **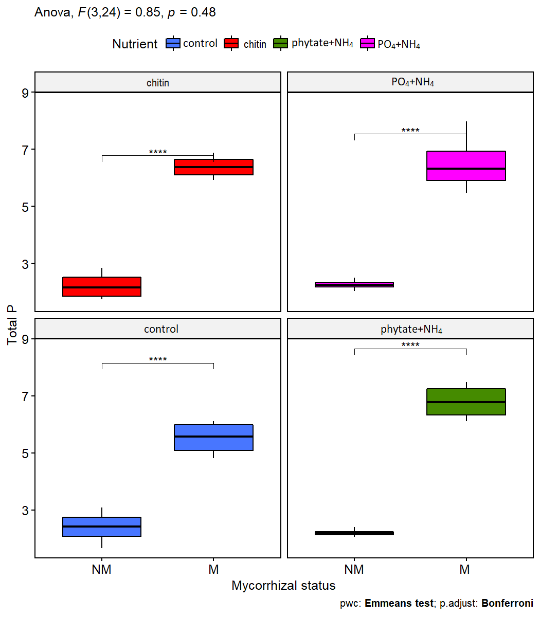** |
| g) Shoot N content (µmol) h) Root N content (µmol) | |
| 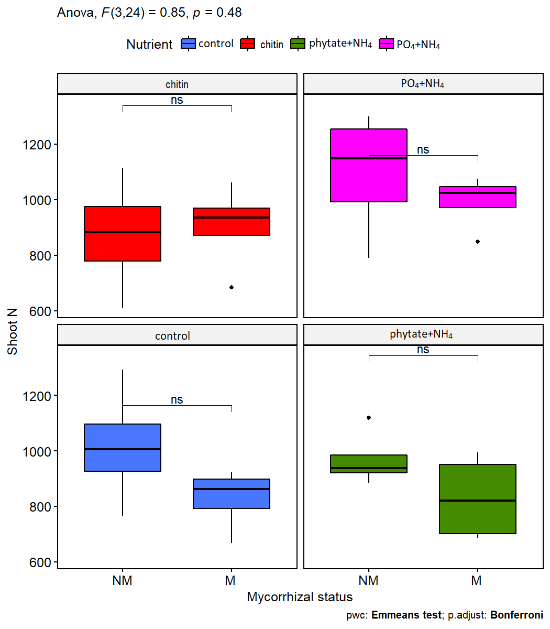 | **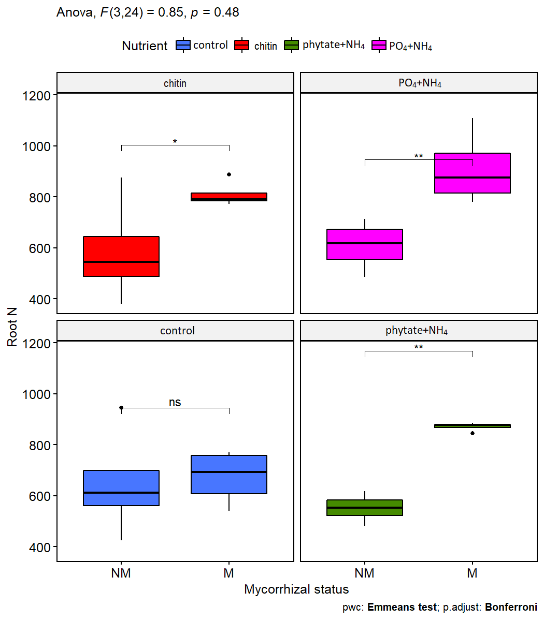** |
| i) Total N content (µmol) | **j) Shoot ^15^N transfer (% of supplied ^15^N)** |
| 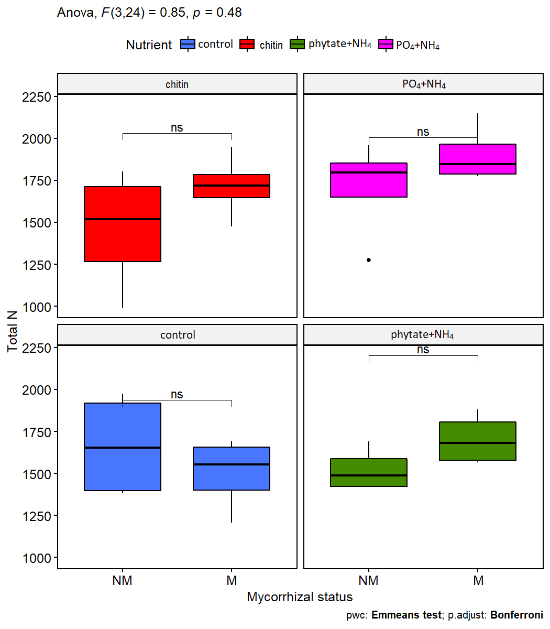 | **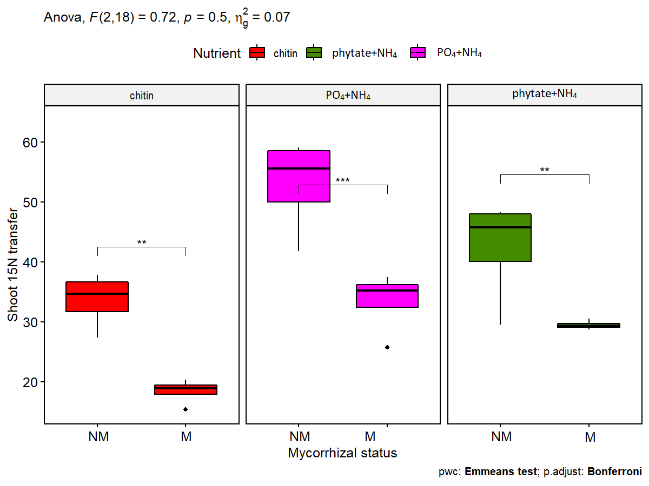** |
| k) Root ^15^N content (% of supplied ^15^N) | **l) Total ^15^N content (% of supplied ^15^N)** |
| 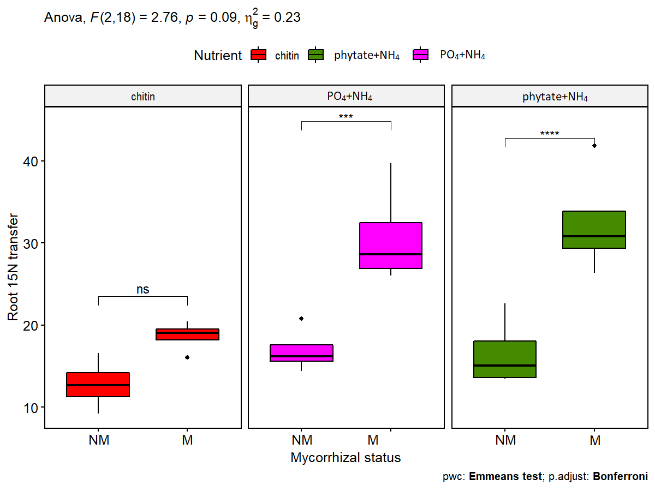 | **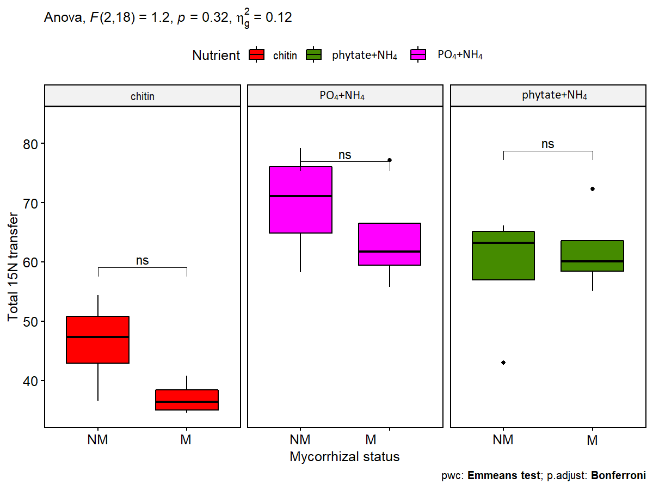** |

**Fig. S1** Two-way ANOVA of the effects of mycorrhizal inoculum, and nutrient supplements, including “control”, “chitin”, “phytate+NH_4_” and “PO_4_+NH_4_” on (a) shoot dry biomass, (b) root dry biomass, (c) total dry biomass, (e) shoot P, (f) root P, (g) total P, (h) shoot N, (i) root N, (j) total N, (j), shoot ^15^N transfer (k), root ^15^N transfer and (l) total ^15^N transfer. Post-hoc multiple pairwise comparisons between groups were performed using the estimated marginal means and P values were adjusted using the Bonferroni correction. The ‘’NM’’ and ‘’M’’ refer to the non-mycorrhizal and mycorrhizal (inoculated with *R.irregularis*) status of the plants, respectively. Asterisks indicate levels of significance; P ≤ 0.05 (*), P ≤ 0.01 (**), P ≤ 0.001 (***) and P ≤ 0.0001 (****).


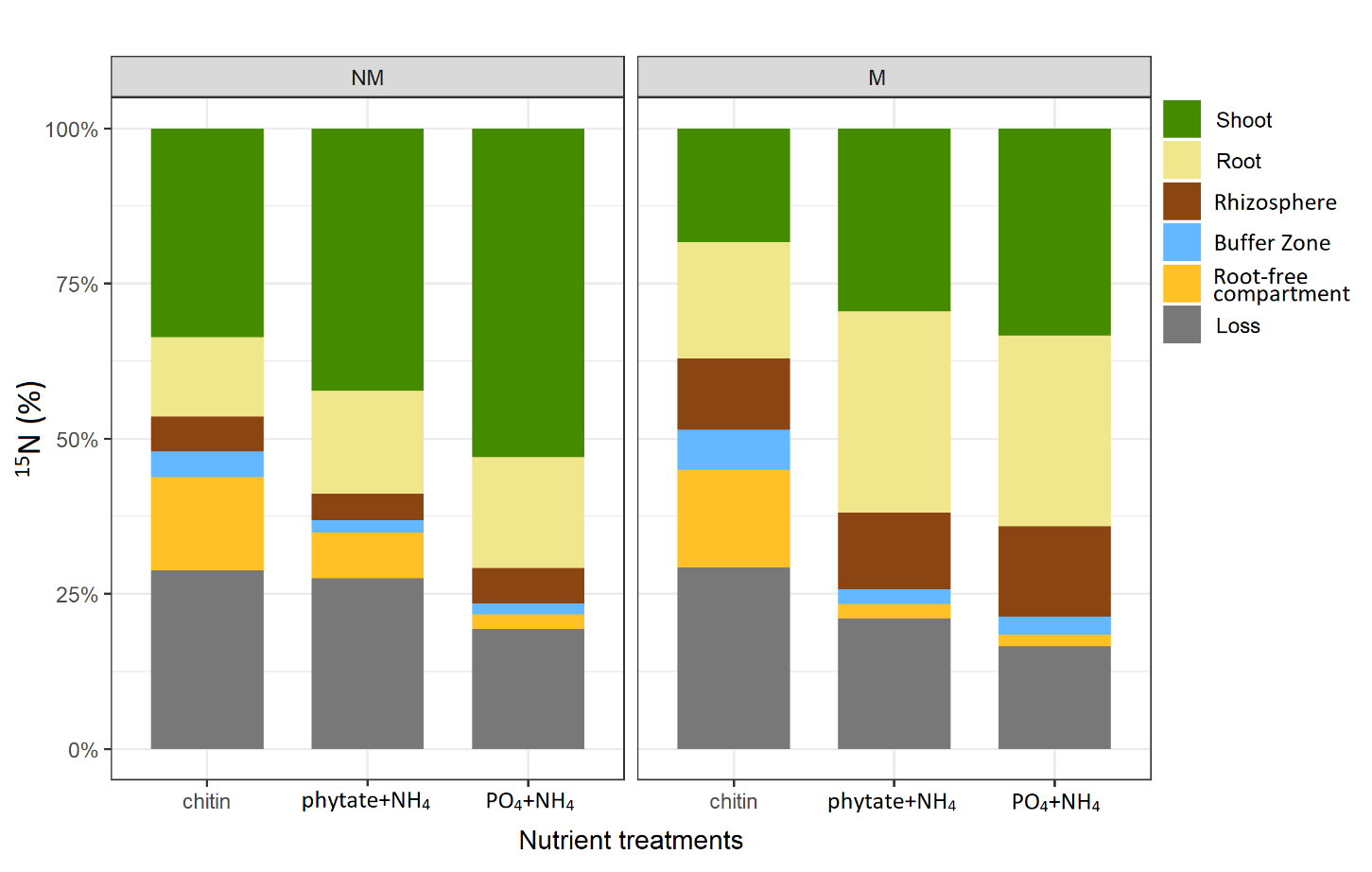


**Fig. S2** ^15^N budget in mycorrhizal and non-mycorrhizal treatments with different nutrient supplements in the root-free compartment. The ^15^N were measured in shoot, root, rhizosphere, buffer zone and root-free compartment. The rest was considered as loss. The ‘’NM’’ and ‘’M’’ refer to the non-mycorrhizal and mycorrhizal (inoculated with *R.irregularis*) status of the plants, respectively.

**Table S1** Results of one-way ANOVA on the effects of different nutrient supplements on mycorrhizal colonization measured by both microscopy and quantitative real-time PCR (qPCR). F and P values are indicated. Significant p values (≤ 0.05) are indicated in bold.

|  | **Nutrient supplements** |
| --- | --- |
| Root hyphal colonization (%) | 0.47 (0.709) |
| Root vesicules colonization (%) | 2.695 (0.093) |
| Root arbuscules colonization (%) | 0.511 (0.682) |
| AM fungal abundance (qPCR) in Root-free compartment (copies mg^-1^ substrate) | 4.014 **(0.034)** |
| AM fungal abundance (qPCR) in rhizosphere (copies mg^-1^ substrate) | 0.288 (0.833) |

**Table S2** Mycorrhizal colonization measured by both microscopy and quantitative real-time PCR (qPCR). Mean ± standard deviation is indicated.

|  | **Control** | **Chitin** | **Phytate+NH_4_** | **PO_4_+NH_4_** |
| --- | --- | --- | --- | --- |
| Root hyphal colonization (%) | 31± 8.41 | 31±11.8 | 32± 11.9 | 39± 12.5 |
| Root vesicules colonization (%) | 4.5± 3 | 2± 1.63 | 4.5± 3.42 | 12±9.38 |
| Root arbuscules colonization (%) | 11.5±4.43 | 13.5±9 | 17±10.9 | 20±15 |
| AM fungal abundance (qPCR) in Root-free compartment (copies mg^-1^ substrate) | 32437±13625 | 38662±28162 | 12326±10134 | 7182±5095 |

**Table S3** Results of two-way ANOVA of the effects of mycorrhizal inoculum and nutrient supplements on abundance of bacteria, ammonia oxidizing bacteria, protists and fungi assessed by quantitative PCR. F and P values are indicated. Significant *p* values (≤ 0.05) are indicated in bold.

|  | **Mycorrhizal**  **inoculation** | **Nutrient**  **supplement** | **Mycorrhizal inoculation × Nutrient supplement** |
| --- | --- | --- | --- |
| Root-free compartment | | | |
| Prokaryotes | 0.49 (0.489) | 6.72 **(0.002)** | 1.08 (0.378) |
| AOB | 10.5 **(0.004)** | 1.04 (0.395) | 27.1 **(0.000)** |
| Protists | 2.57 (0.122) | 5.88 **(0.004)** | 1.47 (0.248) |
| Fungi | 0.19 (0.666) | 11.8 **(0.000)** | 0.45 (0.72) |
| Rhizosphere | | | |
| Bacteria | 21.2 **(0.000)** | 0.27 (0.844) | 1.35 (0.281) |
| AOB | 10.3 **(0.004)** | 1.05 (0.387) | 3.16 **(0.043)** |
| Prokaryotes | 3.84 (0.062) | 0.69 (0.567) | 0.53 (0.669) |
| Fungi | 30.6 **(0.000)** | 1.54 (0.23) | 2.292 (0.104) |

Target sequences used for measurement of abundance of bacteria, ammonia oxidizing bacteria (AOB), protists and fungi were Eub (16S rRNA gene of bacteria), CTO (16S rRNA gene of ammonia-oxidizing bacteria), V4 (V4 region of 18S rRNA gene of protists), and H (Internal transcribed spacer 1 region within the rRNA operon of fungi), respectively.

| Rhizosphere |  |
| --- | --- |
| a) Prokaryotes | **b) Ammonia-oxidizing bacteria** |
| 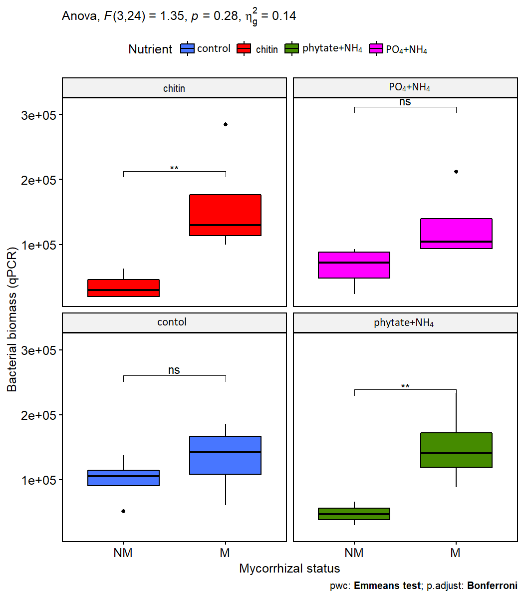 | **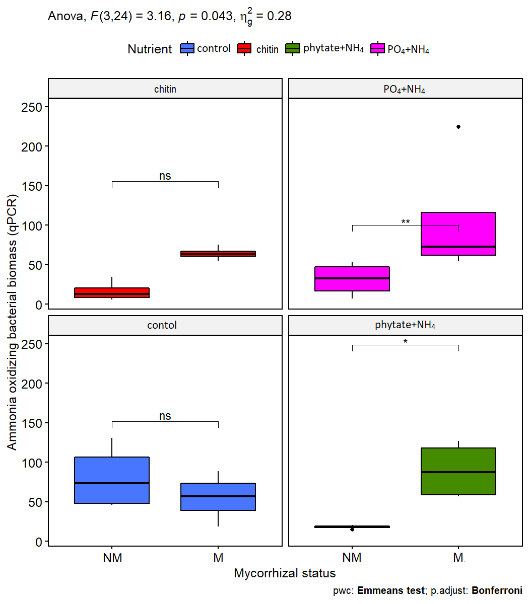** |
| c) Protists | **d) Fungi** |
| 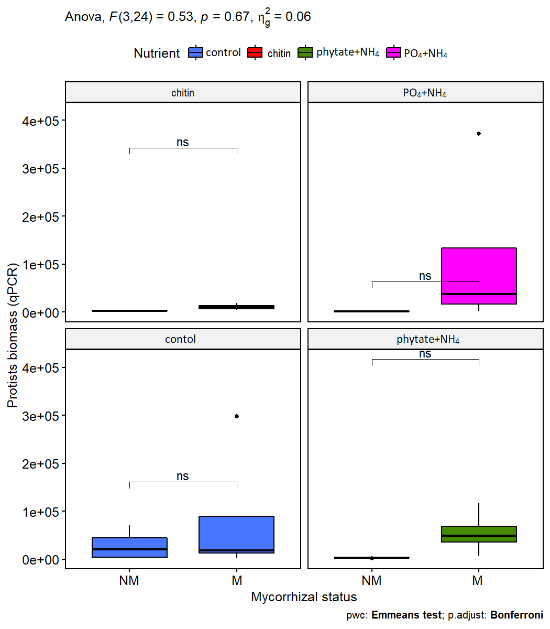 | **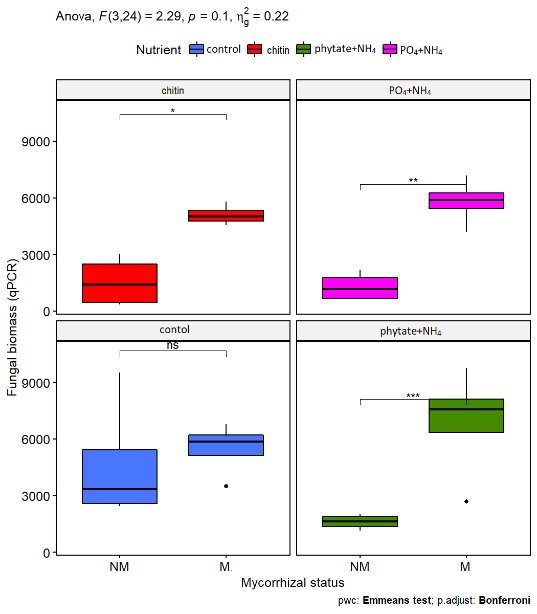** |

**Fig. S3** Results of two-way ANOVA of the effects of mycorrhizal inoculum and nutrient treatments on abundance of a) prokaryotes, b) ammonia-oxidizing bacteria, c) protists and d) fungi in the **rhizosphere** assessed by quantitative real-time PCR (qPCR). Post-hoc multiple pairwise comparisons between groups were performed using the estimated marginal means and P values were adjusted using the Bonferroni correction. The ‘’NM’’ and ‘’M’’ refer to the non-mycorrhizal and mycorrhizal (inoculated with *R. irregularis*) status of the plants, respectively. Asterisks indicate levels of significance; P ≤ 0.05 (*), P ≤ 0.01 (**), P ≤ 0.001 (***) and P ≤ 0.0001 (****).

| Root-free compartment |  |
| --- | --- |
| a) Bacteria | **b) Ammonia oxidizing bacteria** |
| 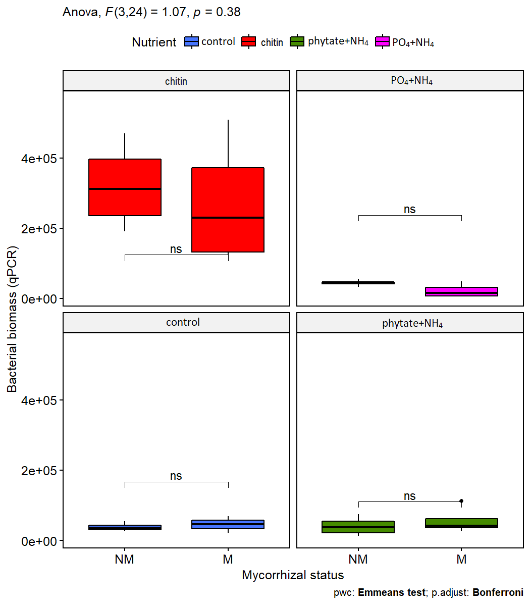 | **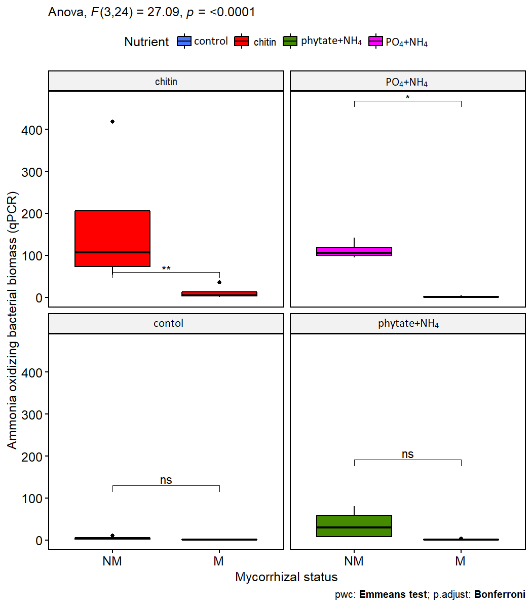** |
| c) Protists | **d) Fungi** |
| 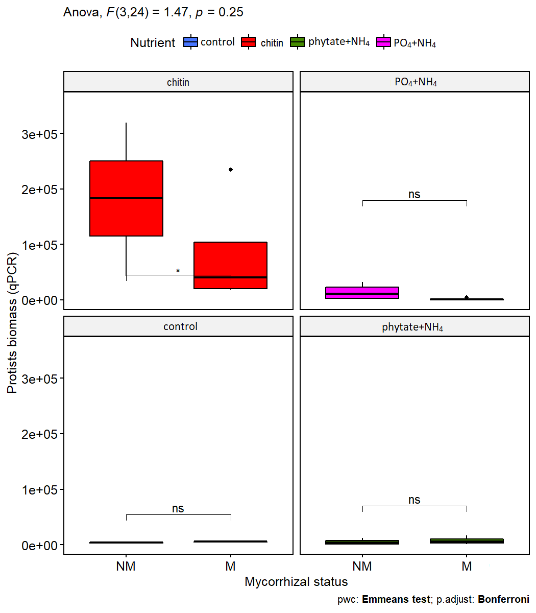 | **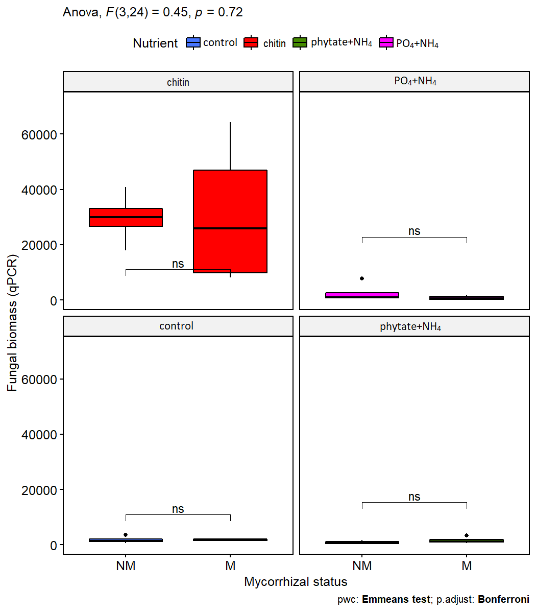** |

**Fig. S4** Results of two-way ANOVA of the effects of mycorrhizal inoculum and nutrient treatments on abundance of a) prokaryotes, b) ammonia oxidizing bacteria, c) protists and d) fungi in **root-free compartment** assessed by quantitative real-time PCR (qPCR). Post-hoc multiple pairwise comparisons between groups were performed using the estimated marginal means and P values were adjusted using the Bonferroni correction. The ‘’NM’’ and ‘’M’’ refer to the non-mycorrhizal and mycorrhizal (inoculated with *R. irregularis*) status of the plants, respectively. Asterisks indicate levels of significance; P ≤ 0.05 (*), P ≤ 0.01 (**), P ≤ 0.001 (***) and P ≤ 0.0001 (****).

**Table S4** Results of two-way ANOVA of the effects of mycorrhizal inoculum and nutrient treatments on Shannon diversity and richness (No. OTUs) of prokaryotes, protists and fungi. F and *p* values are indicated. Significant *p* values (≤ 0.05) are indicated in bold.

|  | **Mycorrhizae**  **Inoculation** | **Nutrient supplement** | | **Mycorrhizae inoculation × Nutrient supplement** |
| --- | --- | --- | --- | --- |
| Root-free compartment | | | | |
| Shannon diversity index of prokaryotes | 1.512 (0.231) | 132.46 **(0.000)** | | 5.660 **(0.004)** |
| Shannon diversity index of protists | 6.56 **(0.017)** | 21.57 **(0.000)** | | 0.620 (0.609) |
| Shannon diversity index of fungi | 0.119 (0.733) | 24.78 **(0.000)** | | 5.398 **(0.006)** |
| Richness (No. OTUs) of prokaryotes | 1.961 (0.174) | 43.06 **(0.000)** | | 1.321 (0.291) |
| Richness (No. OTUs) of protists | 4.562 **(0.043)** | 4.524 **(0.012)** | | 0.299 (0.825) |
| Richness (No. OTUs) of fungi | 0.234 (0.633) | 2.856 (0.058) | | 1.457 (0.251) |
| Rhizosphere | | | | |
| Shannon diversity index of prokaryotes | 1.166 (0.291) | | 2.082 (0.129) | 1.997 (0.141) |
| Shannon diversity index of protists | 10.76 **(0.003)** | | 0.974 (0.421) | 0.376 (0.771) |
| Shannon diversity index of fungi | 3.944 (0.059) | | 0.402 (0.753) | 1.038 (0.394) |
| Richness (No. OTUs) of prokaryotes | 35.86 **(0.000)** | | 0.924 (0.444) | 1.409 (0.264) |
| Richness (No. OTUs) of protists | 0.434 (0.516) | | 1.334 (0.287) | 1.295 (0.299) |
| Richness (No. OTUs) of fungi | 0.033 (0.857) | | 1.112 (0.364) | 2.301 (0.103) |

**Table S5** Shannon diversity and richness (No. OTUs) of prokaryotes, protists and fungi in mycorrhizal (M) and non-mycorrhizal (NM) treatments. Mean ± standard deviation is indicated.

|  | **Control** | **Chitin** | **Phytate+NH_4_** | **PO_4_+NH_4_** |
| --- | --- | --- | --- | --- |
| **Root-free compartment** | | | | |
| Shannon diversity index of prokaryotes | 4.93±0.069 (NM)  4.97±0.101 (M) | 3.3±0.306 (NM)  3.74±0.245 (M) | 4.8±0.193 (NM)  4.88±0.098 (M) | 5.07±0.106 (NM)  4.8±0.082 (M) |
| Shannon diversity index of protists | 2.44±0.399 (NM)  2.35±0.221 (M) | 1.82±0.18 (NM)  1.31±0.298 (M) | 2.95±0.22(NM)  2.73±0.188 (M) | 2.92±0.506 (NM)  2.48±0.577 (M) |
| Shannon diversity index of fungi | 2.98±0.089 (NM)  2.72±0.159 (M) | 2.10±0.207(NM)  2.23±0.19 (M) | 2.59±0.086 (NM)  2.96±0.233 (M) | 2.71±0.202 (NM)  2.56±0.17 (M) |
| Richness (No. OTUs) of prokaryotes | 1516±53 (NM)  1549±33.4 (M) | 1026±152(NM)  974±124 (M) | 1400±207 (NM)  1378±26.1 (M) | 1620±82.1 (NM)  1445±37.7 (M) |
| Richness (No. OTUs) of protists | 133±23.9 (NM)  128±21.9 (M) | 115±10.7 (NM)  92.2±28 (M) | 151± 44.3 (NM)  129±21.8 (M) | 160±18.2 (NM)  133±18.6 (M) |
| Richness (No. OTUs) of fungi | 293±21.1 (NM)  280±21.4 (M) | 262±7.27 (NM)  258±15.4 (M) | 272±35.4 (NM)  292±10.4 (M) | 288±11.2 (NM)  271±18 (M) |
| **Rhizosphere** | | | | |
| Shannon diversity index of prokaryotes | 6.18±0.108 (NM)  6.29±0.082 (M) | 6.17±0.075 (NM)  6.16±0.104 (M) | 5.97±0.232 (NM)  6.17±0.098 (M) | 6.19±0.118 (NM)  6.11±0.054 (M) |
| Shannon diversity index of protists | 3.46±0.43 (NM)  3.03±0.639 (M) | 3.16±0.343 (NM)  2.57±0.263 (M) | 3.17±0.534 (NM)  2.87±0.588 (M) | 3.41±0.336 (NM)  2.67±0.243 (M) |
| Shannon diversity index of fungi | 3.02±0.412 (NM)  3.06±0.273 (M) | 3.11±0.29 (NM)  2.91±0.189 (M) | 3.14±0.243 (NM)  2.64±0.532 (M) | 3.14±0.083 (NM)  2.93±0.221 (M) |
| Richness (No. OTUs) of prokaryotes | 2292±81.3 (NM)  2398±64.3 (M) | 2264±8.22 (NM)  2351±24.2 (M) | 2047±376 (NM)  2338±27.8 (M) | 2348±87.3 (NM)  2312±79.1 (M) |
| Richness (No. OTUs) of protists | 139±18.3 (NM)  134±18.9 (M) | 110±20.1 (NM)  128±14 (M) | 113±33.8 (NM)  131±18.4 (M) | 136±15.2 (NM)  122±8.35 (M) |
| Richness (No. OTUs) of fungi | 308±37.1 (NM)  350±22.9 (M) | 315±31.9 (NM)  336±27.2 (M) | 326±30.6 (NM)  282±61.6 (M) | 338±15.3 (NM)  328±34.3 (M) |

| Root-free compartment | |
| --- | --- |
| a) Prokaryotes (3192 Bacteria and 28 Archaea OTUs) | |
| 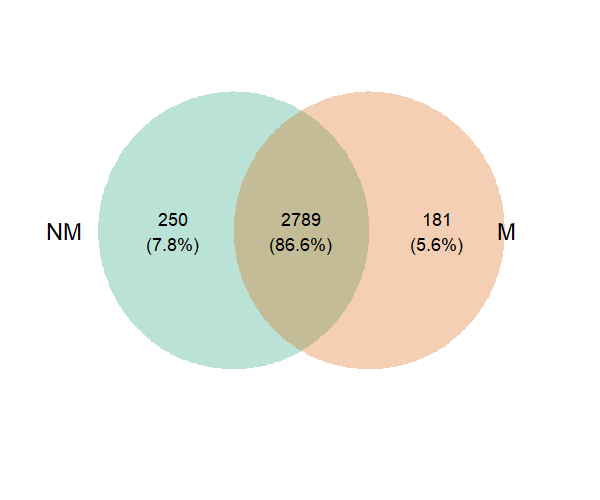 | 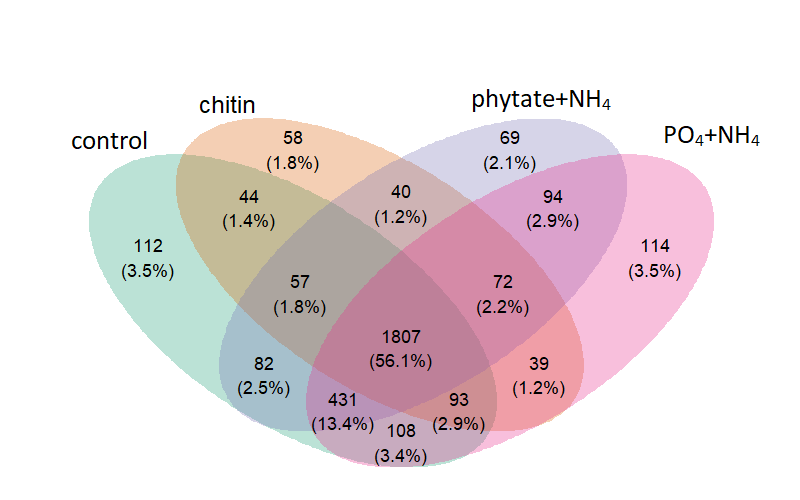 |
| b) Protists (553 OTUs) | |
| 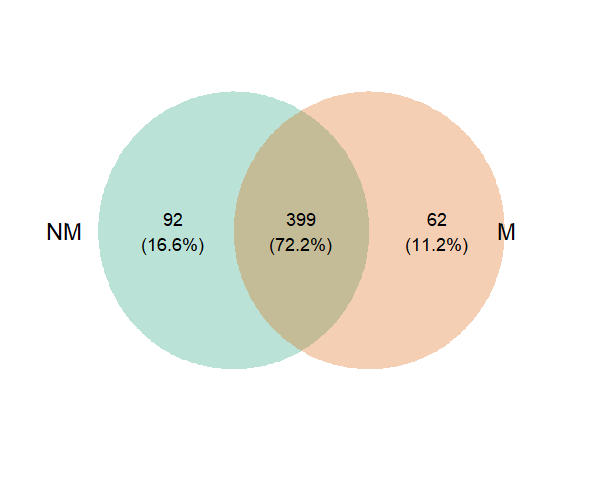 | 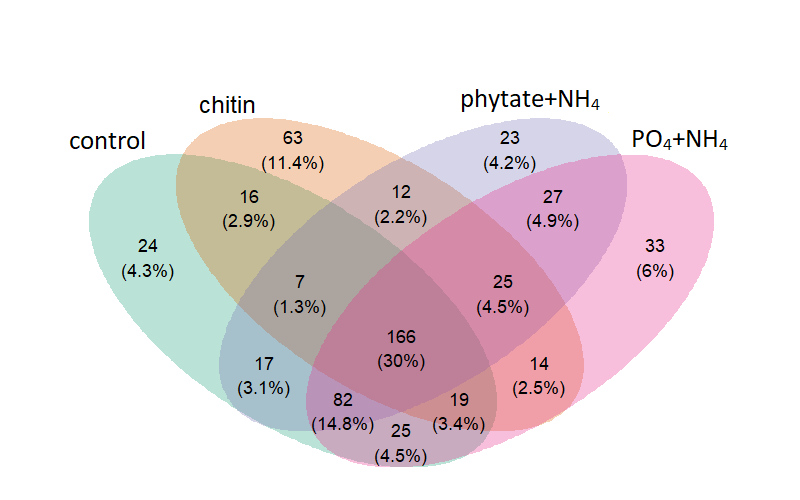 |
| c) Fungi (611 OTUs) | |
| 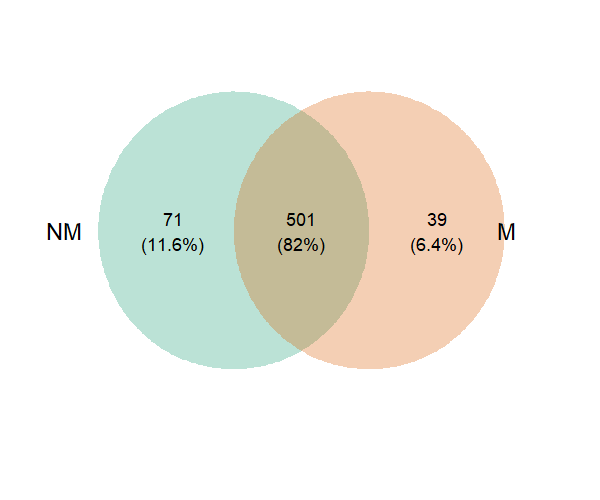 | 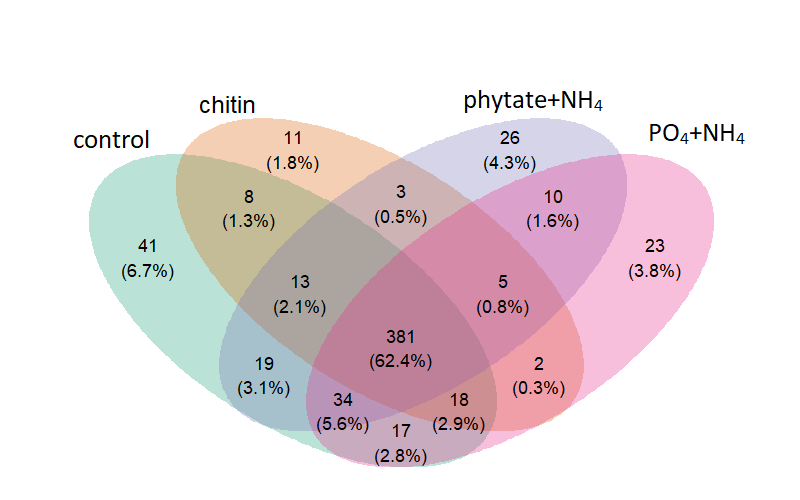 |

**Fig. S5** Venn diagram of shared and unique taxa of prokaryotes, protists and fungi in mycorrhizal and non-mycorrhizal (left panel) and Root-free compartment -compartment nutrient supplement (right panel) treatments. Values indicate the number of OTU's and the percent the proportion of total OTUs.

| **Root-free compartment** |
| --- |
| **a) prokaryotes** |
| 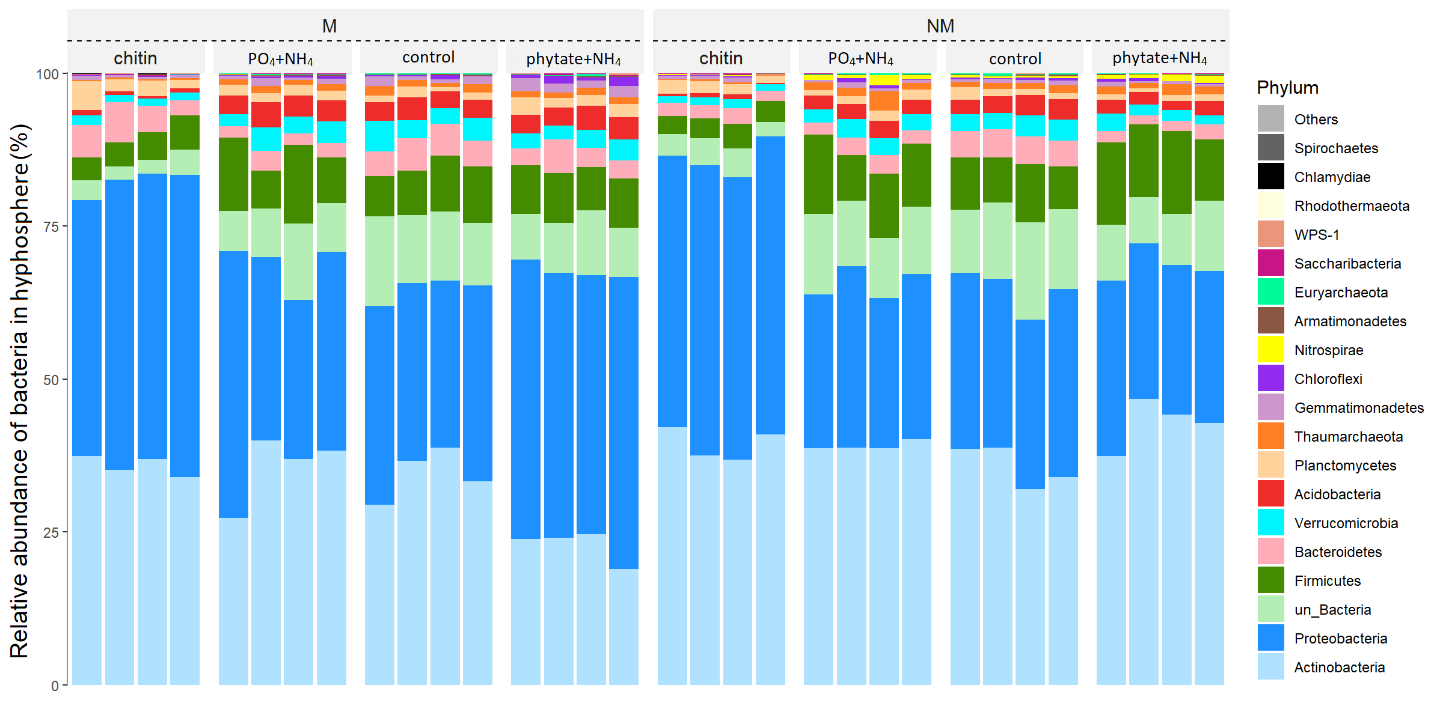 |
| **b) protist** |
| 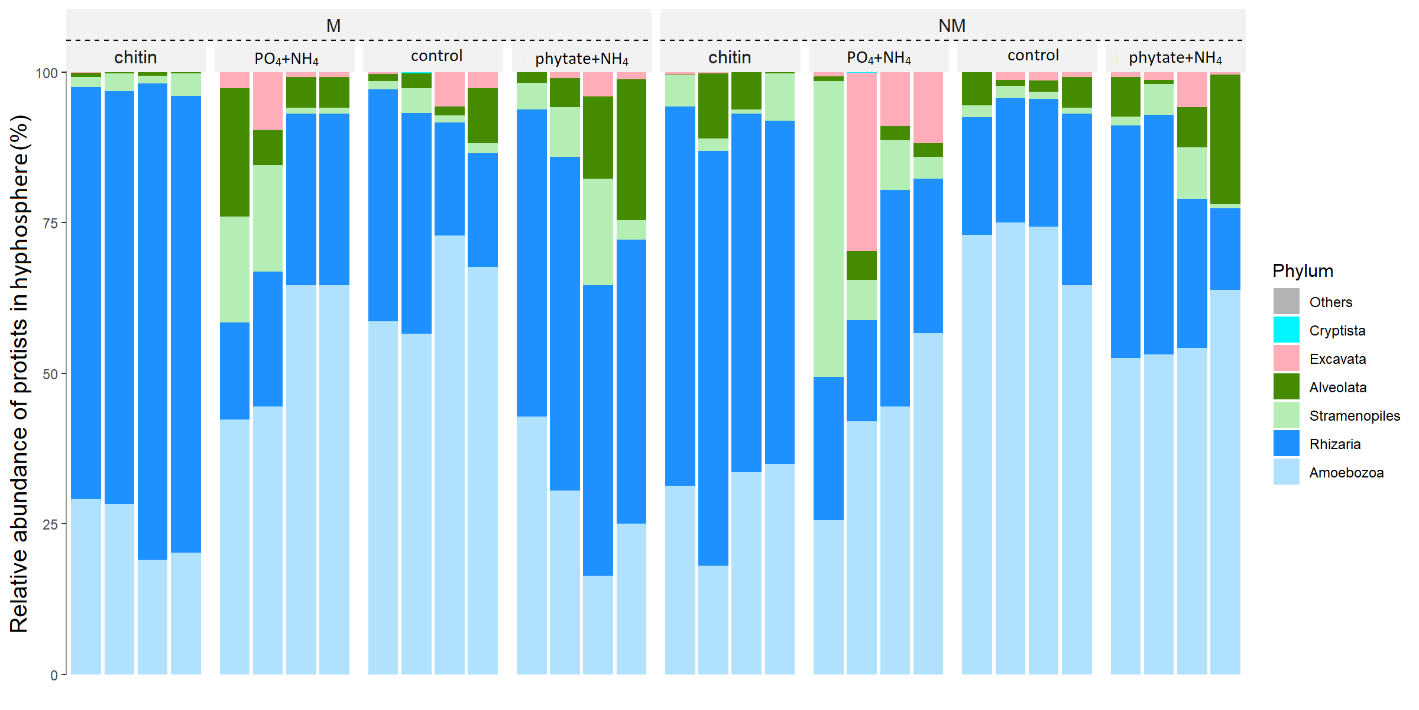 |
| **c) fungi** |
| 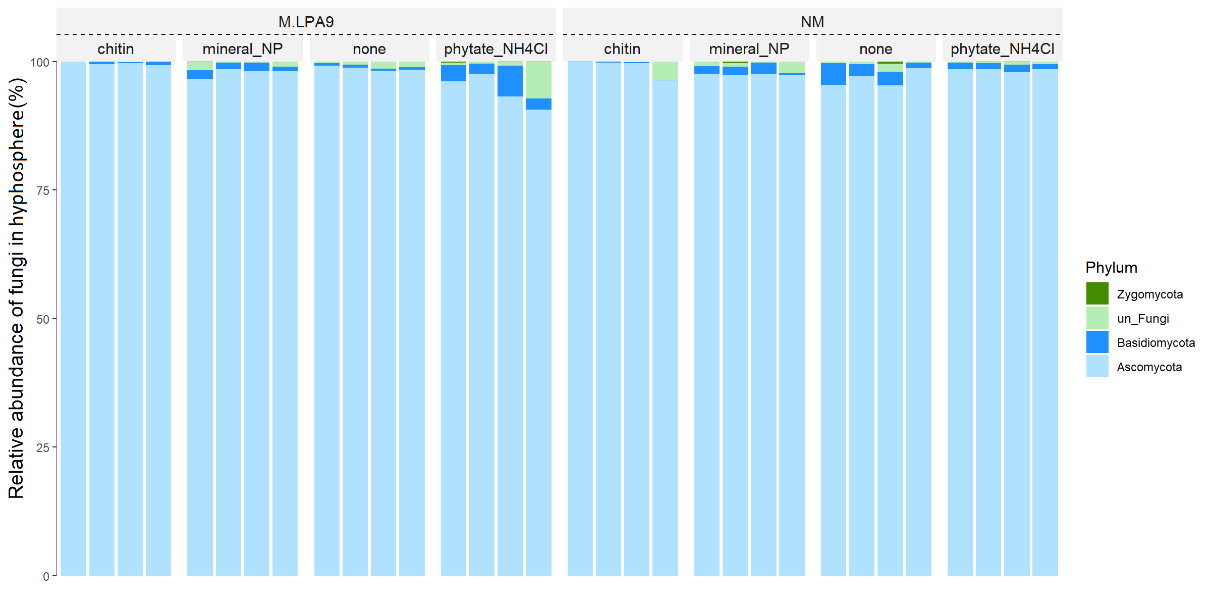 |

**Fig. S6** Relative abundance of prokaryotic (a), protist (b), and fungal (c) communities in the Root-free compartment. The legend lists only the top 20 most abundant taxa.

| Rhizosphere | |
| --- | --- |
| a) Bacteria and archaea (3496 and 28 OTUs in total, respectively) | |
| 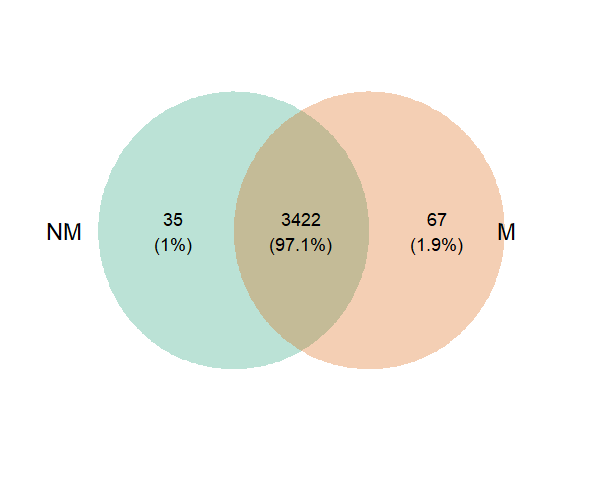 | 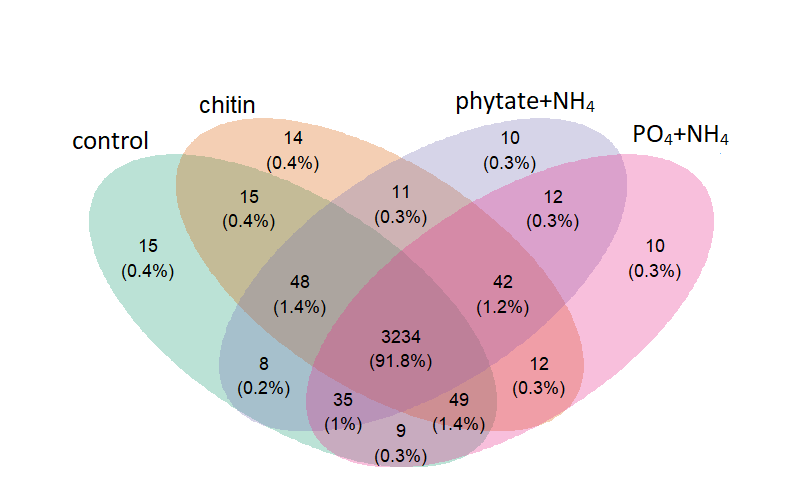 |
| b) Protists (526 OTUs in total) | |
| 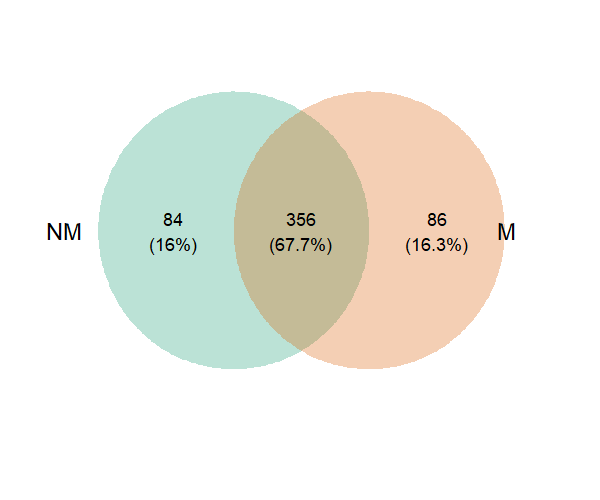 | 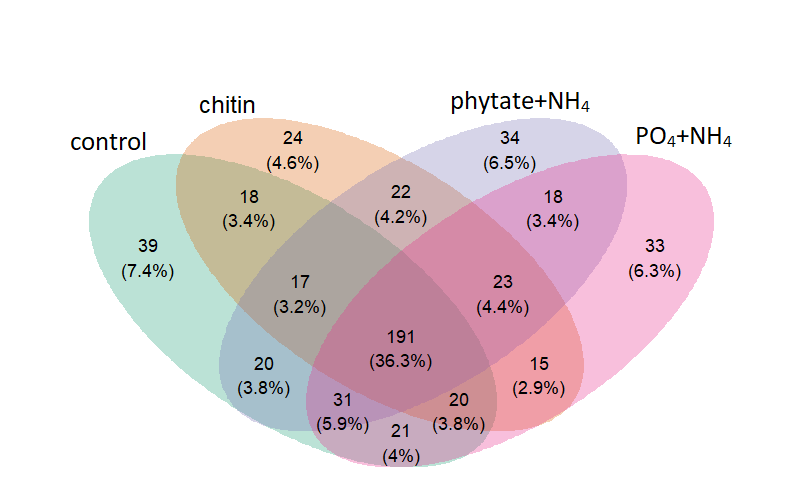 |
| c) Fungi (749 OTUs in total) | |
| 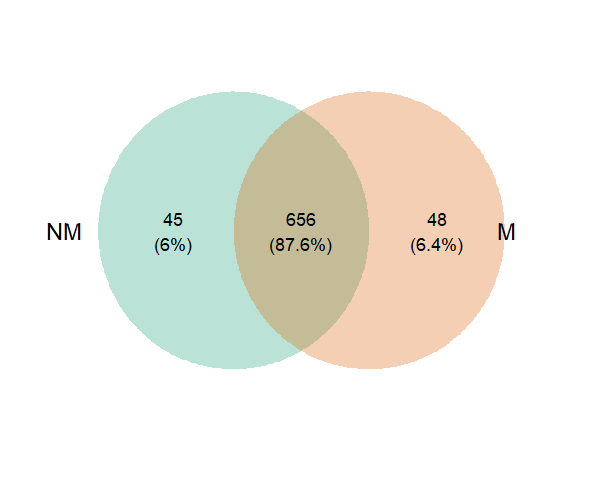 | 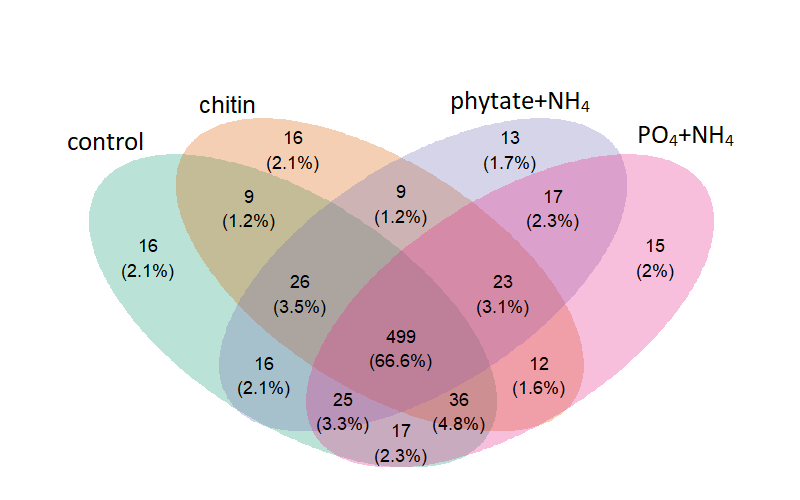 |

**Fig. S7** Venn diagram of shared and unique taxa of bacteria, protists and fungi in mycorrhizal and non-mycorrhizal (left panel) and different nutrient treatments (right pane) in the rhizosphere. Integers indicate the number of OUT’s and percent represents Number of OTUs/total OTUs.

| **Rhizosphere** |
| --- |
| **a) prokaryotes** |
| 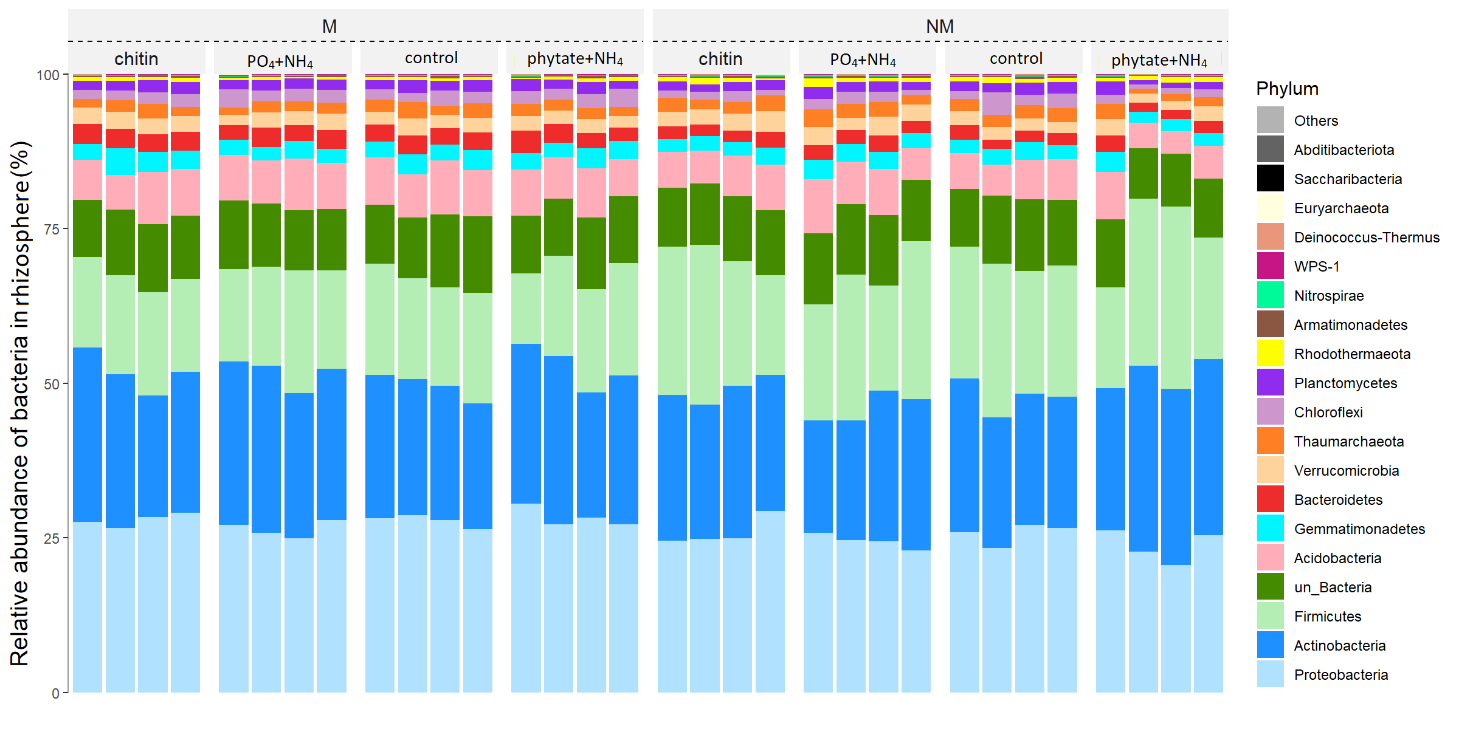 |
| **b) protist** |
| 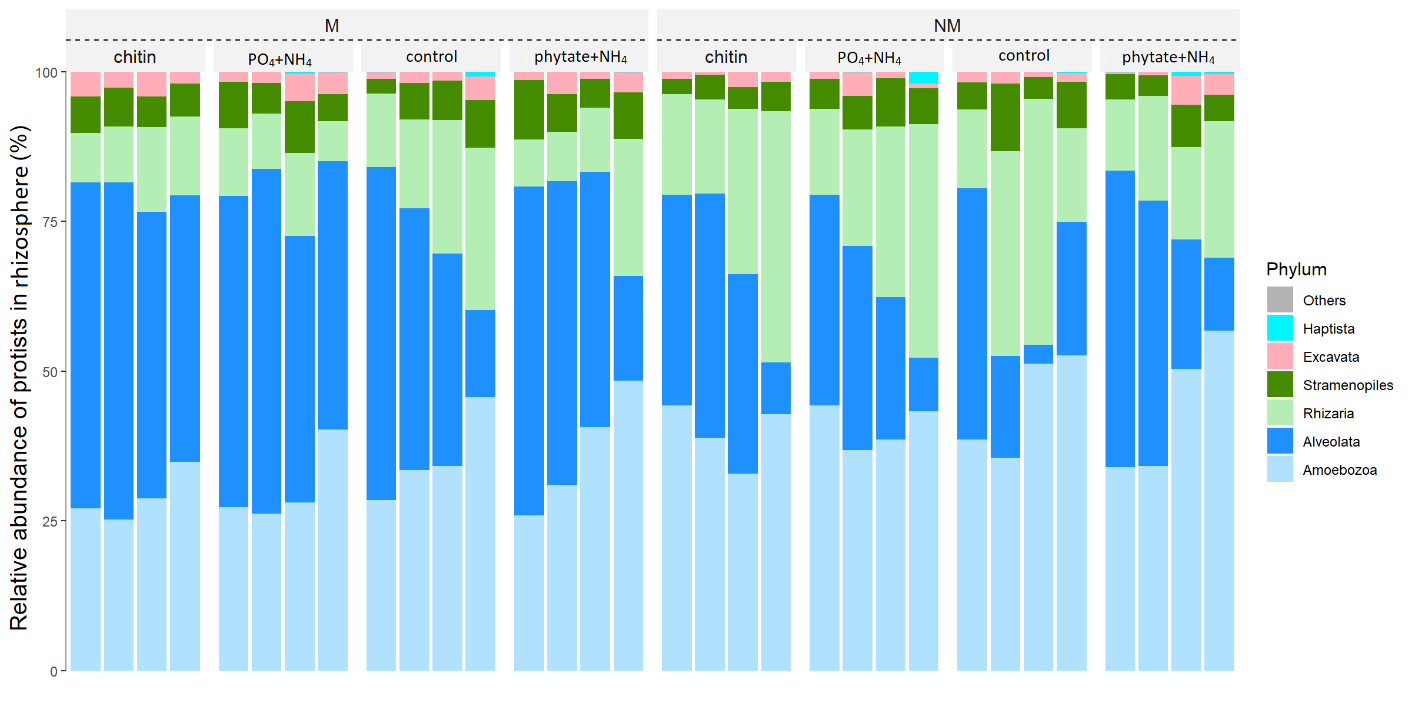 |
| **c) fungi** |
| 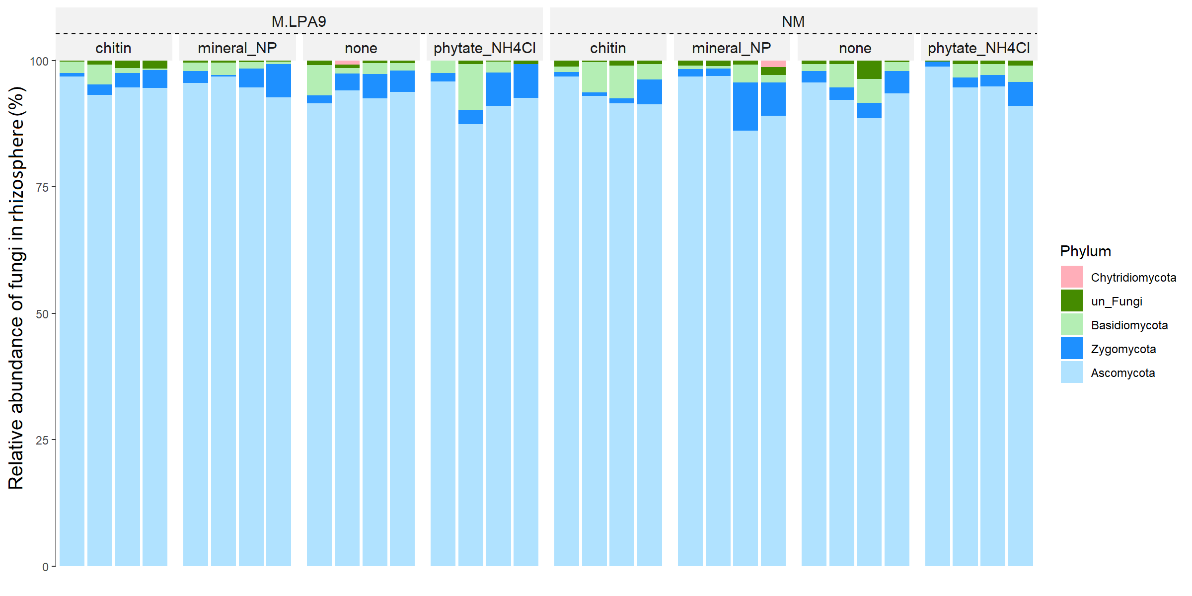 |

**Fig. S8** Relative abundance of prokaryotic (a), protist (b), and fungal (c) communities in the rhizosphere. The legend lists only the top 20 most abundant taxa.

**Table S6** Topological properties of microbial networks in the Root-free compartment of mycorrhizal (M) and nonmycorrhizal treatments in response to different nutrient amendments in the root-free compartment.

|  | **M** | | | | **NM** | | | |
| --- | --- | --- | --- | --- | --- | --- | --- | --- |
|  | **Control** | **Chitin** | **Phytate+NH_4_** | **PO_4_+NH_4_** | **Control** | **Chitin** | **Phytate+NH_4_** | **PO_4_+NH_4_** |
| **No. of nodes** | 72 | 45 | 95 | 93 | 69 | 54 | 72 | 86 |
| **No. of edges** | 73 | 34 | 175 | 133 | 63 | 63 | 84 | 103 |
| **Average degree** | 2.03 | 1.51 | 3.68 | 2.86 | 1.83 | 2.33 | 2.33 | 2.4 |
| **Average path length** | 5.43 | 2.68 | 3.03 | 4.06 | 4.62 | 3.44 | 5.02 | 4.8 |
| Clustering coefficient | 0.02 | 0.08 | 0.05 | 0.02 | 0.00 | 0.02 | 0.03 | 0.04 |
| Graph density | 0.03 | 0.03 | 0.04 | 0.03 | 0.03 | 0.04 | 0.03 | 0.03 |
| Heterogeneity | 0.61 | 0.62 | 0.63 | 0.49 | 0.61 | 0.72 | 0.58 | 0.55 |
| Centralization | 0.06 | 0.08 | 0.07 | 0.06 | 0.05 | 0.11 | 0.05 | 0.07 |
| **Average betweenness centrality** | 131 | 6 | 120 | 166 | 58 | 52 | 135 | 142 |
| **No. of modules** | 10 | 13 | 9 | 11 | 14 | 10 | 11 | 11 |
| **No. of connectors and hubs** | 2 | 0 | 24 | 18 | 2 | 3 | 9 | 5 |
| Average eigenvector centrality | 0.011 | 0.008 | 0.008 | 0.012 | 0.011 | 0.005 | 0.011 | 0.014 |
| Modularity | 0.74 | 0.83 | 0.51 | 0.61 | 0.77 | 0.61 | 0.67 | 0.67 |
| **Prokaryote nodes (%)** | 79 | 80 | 74 | 78 | 77 | 74 | 83 | 78 |
| **Fungal nodes (%)** | 8 | 11 | 14 | 11 | 14 | 11 | 13 | 10 |
| **Protists nodes (%)** | 13 | 9 | 13 | 11 | 9 | 15 | 4 | 12 |

Note: Nodes represent the OTU-level taxa, and the edge connecting two nodes indicates the co-occurrence relationship. The average network degree refers to the average number of edges per node in the graph. Betweenness centrality for each node is the number of shortest paths that pass through the node. The average path length is the average graph distance between all pairs of nodes. Connected nodes have graph distance 1. Network diameter is the shortest distance between the two farthest nodes in the network. The clustering coefficient quantifies the frequency of connected triangles in a network and measures how connected a node's neighbors are to each other. Graph density is a measure of how close the network is to being complete. A complete graph has all possible edges and the density is equal to 1. Heterogeneity calculates the diversity of connections between nodes and reflects how stable and robust a network is with respect to perturbations. Centralization (Freeman 1978) measures a graph-level centrality value based on the node-level centrality measure, indicating the extent to which a given network's connections are concentrated in a single or group of taxa. Eigenvector centrality is used to measure the level of influence of a node within a network. Modules or communities in a network are dense groups of nodes that are closely connected within the group and only loosely connected to the rest of the nodes in the network.

**Table S7** Key connectors and module hub in microbial networks in the root-free compartment of mycorrhizal (M) and non-mycorrhizal (NM) treatments in response to different nutrient amendments in the root-free compartment. Modulus hubs are indicated in bold.

| M | | | |
| --- | --- | --- | --- |
| Unamended control | **chitin** | **phytate+NH_4_** | **PO_4_+NH_4_** |
| Kribbella (Actinobacteria) |  | Kribbella (Actinobacteria) | un_Streptomycetaceae (Actinobacteria) |
| Ohtaekwangia (Bacteroidetes) |  | Rubrobacter (Actinobacteria) | Rubrobacter (Actinobacteria) |
|  |  | Streptomyces (Actinobacteria) | Lentzea (Actinobacteria) |
|  |  | un_Actinobacteria (Actinobacteria) | Noviherbaspirillum (Proteobacteria) |
|  |  | Promicromonospora (Actinobacteria) | un_Proteobacteria (Proteobacteria) |
|  |  | Phenylobacterium (Proteobacteria) | un_Betaproteobacteria (Proteobacteria) |
|  |  | un_Betaproteobacteria (Proteobacteria) | un_Bradyrhizobiaceae (Proteobacteria) |
|  |  | un_Phyllobacteriaceae (Proteobacteria) | un_Polyangiaceae (Proteobacteria) |
|  |  | Arenimonas (Proteobacteria) | Ramlibacter (Proteobacteria) |
|  |  | Mesorhizobium (Proteobacteria) | Sandaracinus (Proteobacteria) |
|  |  | un_Gammaproteobacteria (Proteobacteria) | Cupriavidus (Proteobacteria) |
|  |  | Azohydromonas (Proteobacteria) | **un_Gemmatimonadaceae (Gemmatimonadetes)** |
|  |  | un_Rhodospirillales (Proteobacteria) | un_Gemmatimonadetes (Gemmatimonadetes) |
|  |  | un_Gemmatimonadaceae (Gemmatimonadetes) | Fictibacillus (Firmicutes) |
|  |  | un_Anaerolineae (Chloroflexi) | Brevibacillus (Firmicutes) |
|  |  | Ohtaekwangia (Bacteroidetes) | Paenibacillus (Firmicutes) |
|  |  | un_Paenibacillaceae (Firmicutes) | Davidiella (Ascomycota) |
|  |  | un_Opitutaceae (Verrucomicrobia) | Amoebozoa (Amoebozoa) |
|  |  | Aspergillus (Ascomycota) |  |
|  |  | Amoebozoa (Amoebozoa) |  |
|  |  | Engyodontium (Ascomycota) |  |
|  |  | Leptosphaerulina (Ascomycota) |  |
|  |  | Pseudodendromonadales (Stramenopiles) |  |
|  |  | Limnofilidae (Rhizaria) |  |
| NM | | | |
| Unamended control | **Chitin** | **Phytate+NH_4_** | **PO_4_+NH_4_** |
| Promicromonospora (Actinobacteria) | Noviherbaspirillum (Proteobacteria) | Micromonospora (Actinobacteria) | Streptomyces (Actinobacteria) |
| Eupenicillium (Ascomycota) | Brevibacillus (Firmicutes) | un_Deltaproteobacteria (Proteobacteria) | Pseudonocardia (Actinobacteria) |
|  | Filamoeba (Amoebozoa) | **un_Erythrobacteraceae (Proteobacteria)** | **Phenylobacterium (Proteobacteria)** |
|  |  | Aquicella (Proteobacteria) | un_Rhizobiales (Proteobacteria) |
|  |  | un_Bacillales (Firmicutes) | Ammoniphilus (Firmicutes) |
|  |  | Cohnella (Firmicutes) |  |
|  |  | Engyodontium (Ascomycota) |  |
|  |  | Amoebozoa (Amoebozoa) |  |
|  |  | Malassezia (Basidiomycota) |  |

| 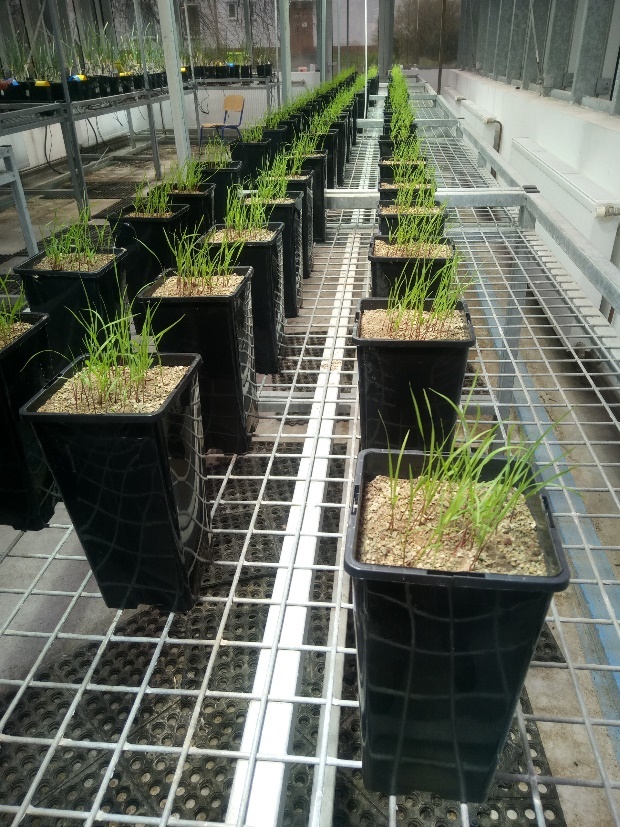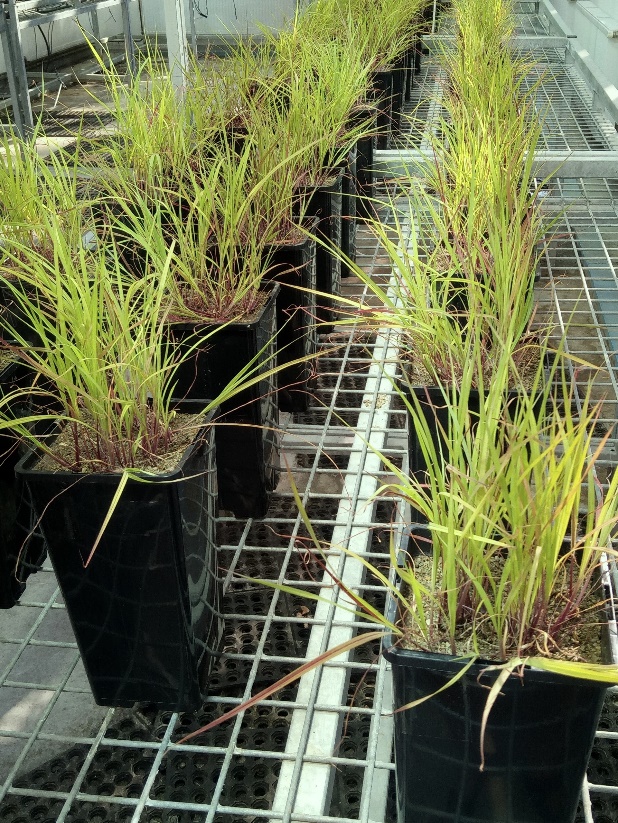 | 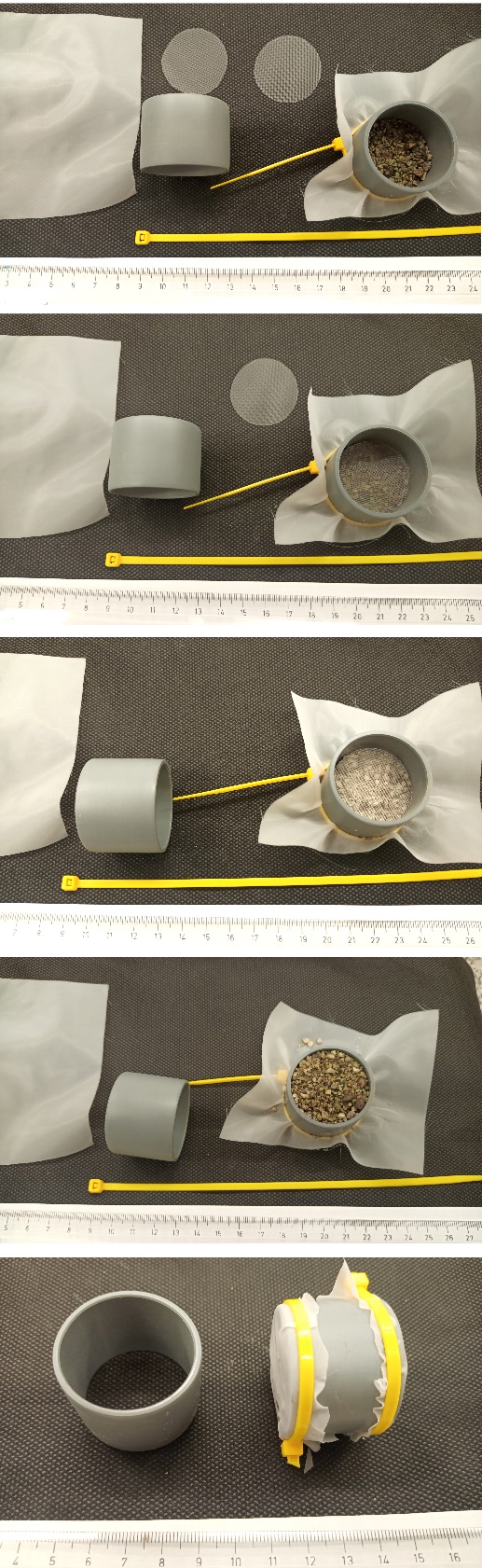 |
| --- | --- |

Fig. S9 Experimental pots with the *Andropogon gerardii* plant roots inoculation with AM fungus, *Rhizophagus irregularis* BEG 236. A small root-free compartment was made of PVC cylinder (3.6 cm diameter x 3 cm length) filled with 40 g potting substrate that was covered with a 40 µm nylon filter. In the middle of the root-free compartment, 4 g of potting substrate amended with various types of P and/or N sources was sandwiched between 200 µm nylon filters.
